# Uncovering an alternate pathway of antibiotic resistance in spore-forming bacteria

**DOI:** 10.1038/s41467-026-75594-5

**Published:** 2026-07-15

**Authors:** Yogitha N. Srikhanta, Clara E. Bate, Desirel Ng, Sarah A. Revitt-Mills, Georgia-Rose Gilmore, Galain C. Williams, Sophie L. Day, Stéphane Mesnage, Kamila Kochan, Shailab Shrestha, Aimee Shen, Daniel R. Knight, Korakrit Imwattana, Thomas V. Riley, Irene Alevizos, Kimberley Bourke, Milena M. Awad, Caroline A. Evans, Ghizal Siddiqui, Joel R. Steele, David L. Steer, Joshua P. Morrow, Darren J. Creek, Chaille Webb, Sheena McGowan, Dena Lyras

**Affiliations:** 1 Infection Program, Monash Biomedicine Discovery Institute and Department of Microbiology, Monash University, Clayton, VIC Australia; 2 Centre to Impact AMR, Monash University, Melbourne, VIC Australia; 3 Barwon Health University Hospital, Geelong, VIC Australia; 4 Molecular Microbiology, School of Biosciences, University of Sheffield, Sheffield, UK; 5 The Florey Institute of Infection, University of Sheffiel, Sheffield, UK; 6 School of Chemistry, Faculty of Science, Monash University, Clayton, VIC Australia; 7 Department of Molecular Biology and Microbiology, Tufts University School of Medicine, Boston, MA USA; 8 Program in Molecular Microbiology, Tufts University Graduate School of Biomedical Sciences, Boston, MA USA; 9 Department of Microbiology, PathWest Laboratory Medicine, Queen Elizabeth II Medical Centre, Perth, WA Australia; 10 School of Biomedical Sciences, The University of Western Australia, Perth, WA Australia; 11 Department of Microbiology, Faculty of Medicine Siriraj Hospital, Mahidol University, Bangkok, Thailand; 12 School of Chemical, Materials and Biological Engineering, University of Sheffield, Sheffield, UK; 13 Drug Delivery, Disposition and Dynamics, Monash Institute of Pharmaceutical Sciences, Monash University, Parkville, VIC Australia; 14 Monash Proteomics & Metabolomics Platform, Department of Biochemistry and Molecular Biology, Biomedicine Discovery Institute, Monash University, Clayton, VIC Australia

**Keywords:** Antimicrobial resistance, Bacteriology, Antibacterial drug resistance, Pathogens

## Abstract

Spore-forming bacteria produce two distinct cell types: vegetative cells and resilient spores. While antibiotic resistance is typically associated with vegetative cells, spores play a critical role in disseminating resistance genes due to their durability and transmissibility. We previously demonstrated that cephamycin antibiotics target the conserved spore-specific protein SpoVD, significantly reducing spore formation in pathogens including *Clostridioides difficile*. Here, we show that when *C. difficile* acquires *CdmecA*, a homologue of *Staphylococcus aureus mecA*, one of the most globally burdensome resistance genes, the anti-sporulation effect of cephamycins is bypassed. *Cd*MecA functionally replaces *Cd*SpoVD, restoring sporulation and producing phenotypically distinct spores. We further show that *mecA* is prevalent across *C. difficile* strains and other pathogenic, gut, and environmental spore-formers. Since SpoVD is conserved, MecA may broadly co-opt sporulation; we confirm this in *Clostridium perfringens*. This work reveals an unusual resistance mechanism with unexpected physiological consequences, reshaping our understanding of antibiotic resistance within the context of sporulation and microbial adaptation.

## Abstract (web summary)

Cephamycin antibiotics inhibit sporulation of the pathogenic bacterium *Clostridioides difficile* by targeting the spore protein SpoVD. Here, the authors show that isolates carrying the resistance gene *mecA* are able to form spores in the presence of cephamycins, with MecA functionally replacing SpoVD.

## Introduction

Bacteria have evolved remarkable strategies to survive extreme environments, one of the most effective being the formation of spores, which are dormant, highly resistant cells. In many pathogenic species, spores are not just survival tools; they are the infectious agents responsible for transmission, persistence and relapse. The opportunistic pathogen *Clostridioides difficile*^1^, the leading cause of hospital-associated diarrhoea worldwide^2^, exemplifies this threat. Beyond healthcare settings, there is an increasing problem of community-acquired *C. difficile* infections (CDI)^3^, likely driven by zoonotic and environmental sources^1^. In these contexts, spores are the primary vehicle for infection and reinfection, posing a growing risk to human and animal health. Understanding how spores contribute to CDI spread is critical in controlling this growing health challenge.

High antibiotic use is common across *C*. *difficile* reservoirs, driving the emergence, persistence, and spread of antimicrobial resistance (AMR)^1,4^. As a result, *C. difficile* is classified as an urgent AMR threat by the U.S. Centers for Disease Control and Prevention^5^. Resistance genes are known to protect vegetative *C*. *difficile* cells^6,7^, but their influence on spores, or the process of sporulation, remains unknown. This gap is critical, as spores are central to transmission and recurrence, especially in environments with heavy antibiotic exposure. Understanding how resistance genes interact with sporulation could reveal hidden mechanisms of persistence and reshape strategies for controlling *C*. *difficile* infections.

We previously demonstrated that cephamycins, a group of β-lactam antibiotics, bind to the essential sporulation-specific penicillin-binding protein (PBP) *Cd*SpoVD, blocking sporulation and preventing recurrent CDI^8^. Here we show that this anti-sporulation effect varies across *C. difficile* isolates due to the acquisition and activation of *Cd*MecA, which confers cephamycin resistance. *Cd*MecA functionally replaces *Cd*SpoVD, enabling sporulation to proceed in the presence of cephamycins, and produces spores that are more resilient to physical stress. Importantly, we found that dual therapy with cefotetan and ceftobiprole overcomes this resistance by simultaneously targeting *Cd*SpoVD and *Cd*MecA, effectively abolishing spore production. This unusual resistance mechanism reveals a direct link between sporulation and antibiotic resistance, challenging conventional views and highlighting a new dimension of microbial survival.

## Results

### *Cd*MecA confers resistance to cephamycin-mediated sporulation inhibition

**Fig. 1:**
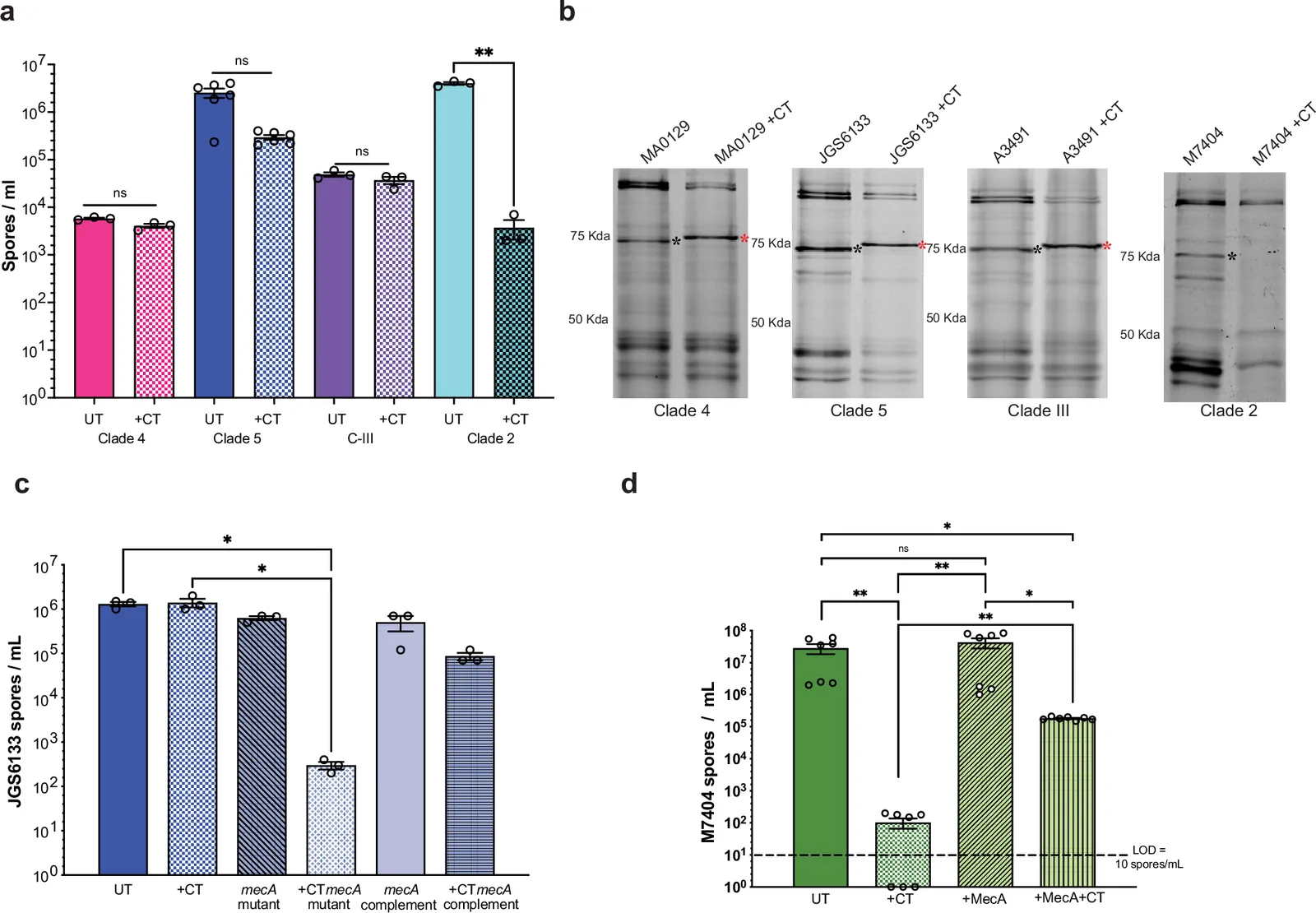
The identification and effect of accessory PBP *Cd*MecA on *C. difficile* sporulation. **a** Using sporulation assays, spore numbers (spores per ml) were determined for MA0129 (Clade 4), JGS6133 (Clade 5), and A3491 (Clade III) cultures, which were either untreated or treated with a sub-inhibitory concentration of cefotetan (MA0129 20 μg/mL, JGS6133 5 μg/mL and A33491 20 μg/mL cefotetan). M7404 (Clade 2) was used as a control. A significant difference was only seen between M7404 untreated and cefotetan-treated spore numbers (*P* = 0.0040). No statistical significance was observed between untreated and cefotetan-treated MA0129, JGS6133 and A3491 (*P* > 0.9999) spore numbers. **b** Sporulating MA0129, JGS6133, A3491 and M7404 cultures were left untreated or cefotetan-treated before isolation of membrane proteins. Isolated proteins were incubated with Boc-FL and separated by SDS–PAGE. Labelled PBPs were visualised using a FluorImager. Black asterisks indicate *Cd*SpoVD visualised in all untreated cultures but not visualised in cefotetan-treated cultures (*Cd*SpoVD bound to cefotetan and therefore cannot bind to Boc-FL); red asterisks indicate *Cd*MecA present in MA0129, JGS6133 and A3491 cefotetan-treated cultures (*Cd*MecA visualised as bound to Boc-FL) but not in untreated cultures (no cefotetan to induce *Cd*MecA expression). **c** JGS6133 spore numbers were determined from untreated and cefotetan-treated wild-type, untreated and cefotetan-treated *CdmecA* mutant, and cefotetan-treated *CdmecA* complemented cultures. A significant difference in spore numbers was observed for untreated wild-type and cefotetan-treated *CdmecA* mutant (*P* = 0.0357) and cefotetan-treated wild-type and cefotetan-treated *CdmecA* mutant (*P* = 0.0357). **d** M7404 spore numbers were determined between untreated or cefotetan-treated wild-type and MecA^+^ cultures. A significant difference was seen between untreated and cefotetan-treated wild-type (**P = 0.009), cefotetan-treated wild-type and untreated MecA^+^ (**P = 0.009), cefotetan-treated wild-type and cefotetan-treated MecA^+^ (**P = 0.008), untreated MecA^+^ and cefotetan-treated MecA^+^ (*P = 0.010), untreated and cefotetan-treated MecA^+^ (*P = 0.010) spore numbers. UT=untreated; +CT=cefotetan-treated; MecA^+^=*CdmecAIR*^*+*^. For sporulation assays, results are the average of three to seven independent biological replicates. Error bars represent s.e.m.; *P*-values were determined using a Kruskal–Wallis test with Dunn’s pairwise comparisons. Limits of detection (LOD) are depicted as a horizontal dotted line. Source data are provided as a Source Data file.

*C. difficile* strains are grouped into five genetically distinct Clades (1–5) and three cryptic Clades (CI-CIII)^9^. We previously showed that a cephamycin antibiotic, cefotetan, inhibits sporulation in Clades 1–3 and CI-II strains by binding and inhibiting the sporulation-specific PBP *Cd*SpoVD^8^. However, Clade 4 (MA0129), 5 (JGS6133) and C-III (A3491) strains were unaffected; sporulation remained high and comparable to untreated controls (Fig. 1a). Notably, vegetative cell counts were unchanged at the single-dose sub-inhibitory cefotetan dose used, confirming that the effect was specific to sporulation (ref. ^8^; Supplementary Table 1, Supplementary Fig. 1a). These findings suggest the presence of resistance mechanisms that bypass *Cd*SpoVD inhibition.

To investigate why cefotetan failed to inhibit sporulation in certain *C*. *difficile* strains, we compared their PBP profiles to M7404 (Clade 2), where sporulation is blocked (Fig. 1b^8^). Using an established method, spore membrane proteins from untreated or cefotetan-treated MA0129 (Clade 4), JGS6133 (Clade 5), and A3491 (Clade C-III) were labelled with BOCILLIN-FL (Boc-FL), a fluorescent penicillin derivative that binds active PBPs. PBPs already bound by cefotetan remain undetectable. These PBP profiles revealed the presence of an additional ~76 kDa PBP, only seen in cefotetan-treated samples but not in cefotetan-treated M7404 (Fig. 1b). Tandem mass spectrometry and Conserved Domain Architecture Retrieval Tool analysis identified this protein as MecA, containing both MecA and transpeptidase domains (Supplementary Fig. 2a), designated here as *Cd*MecA. *Cd*MecA resembles PBP2a (MecA) from the non-spore-forming pathogen *Staphylococcus aureus*; this protein is well known to confer vegetative cell resistance against β-lactam antibiotics, e.g., methicillin and cefoxitin^10^^,^^11^. Comparative analysis of AlphaFold2 models of *Cd*MecA with an X-ray crystal structure of *S. aureus* (PBP2a; PDB ID:1VQQ; 32.2% identity, 51.3% similarity) revealed similar folds between the two proteins (Supplementary Fig. 2b, Supplementary Table 2), supporting its role in antibiotic resistance and sporulation.

*Cd*SpoVD is essential for *C. difficile* spore peptidoglycan synthesis^12^. We hypothesised that *Cd*MecA compensates for cephamycin-mediated *Cd*SpoVD inactivation, enabling sporulation to continue in the presence of cephamycins. To examine this, we chose strain JGS6133 because it mirrored responses observed across multiple clades and had genetic tractability. Sporulation assays using a JGS6133 *CdmecA* mutant confirmed this: cefotetan effectively inhibited sporulation in the absence of *Cd*MecA (Fig. 1c; Supplementary Fig. 1b, c). This role was further validated by introducing *CdmecA* into the non-*CdmecA-*carrying strain, M7404, which is sensitive to cephamycin-induced sporulation inhibition^8^. In cefotetan-treated M7404 expressing *Cd*MecA, sporulation was partially restored (Fig. 1d, Supplementary Fig. 1d, e), revealing a novel resistance mechanism whereby *Cd*MecA bypasses *Cd*SpoVD inhibition and preserves sporulation under cephamycin pressure.

To assess whether *CdmecA* changed cefotetan resistance in vegetative cells, we performed minimum inhibitory concentration (MIC) assays. *Cd*MecA had no effect, with resistance levels remaining unchanged. This outcome is expected as *C. difficile* vegetative cells are intrinsically resistant to cephamycins (Supplementary Table 3^8^), confirming that *Cd*MecA’s role in the context of cephamycin exposure is specific to sporulation, not vegetative cell survival.

The inability of cefotetan to block sporulation in *Cd*MecA-expressing strains suggested that cephamycins fail to effectively bind *Cd*MecA. To test this, we produced a truncated recombinant form of *Cd*MecA (*Cd*MecA_∆1-34_), removing a transmembrane helix to produce a soluble form of the protein, and performed fluorescence polarisation assays. These measured cefotetan’s ability to compete with Boc-FL for binding at the PBP active site. Cefotetan failed to displace Boc-FL from *Cd*MecA _∆1-34_, even at concentrations up to 4000 μM, confirming that *Cd*MecA is not efficiently targeted by cephamycins (Supplementary Fig. 3a). In comparison, previously published data reported that cefotetan binds *Cd*SpoVD with high nanomolar affinity^8,13^. Binding of cefotetan to *Cd*SpoVD was assessed as a positive control and confirmed cefotetan to have nanomolar affinity for *Cd*SpoVD (IC_50_ 26.5 ± 10; Supplementary Fig. 3b).

Like *S. aureus mecA, CdmecA* is encoded in a *mec* gene complex comprised of *CdmecA* and convergently transcribed regulatory genes *CdmecR* (antibiotic sensor) and *CdmecI* (antibiotic repressor)^14^ (Supplementary Notes 1, Supplementary Fig. 2a). AlphaFold2 models suggest strong structural and functional similarity to their *S. aureus* counterparts (Supplementary Notes 1, Supplementary Fig. 2c, Supplementary Table 2). Functional assays confirmed that *CdmecR* is required for *Cd*MecA expression; its disruption blocked cefotetan-induced *Cd*MecA expression and abolished sporulation. In contrast, disruption of *CdmecI* had no effect on cefotetan-induced *Cd*MecA induction or sporulation (Supplementary Notes 1, Supplementary Fig. 2d–f). Consistent with this, digital PCR (Supplementary Fig. 2g) revealed that *CdmecA* transcription is induced by cefotetan and differentially regulated in mutant backgrounds. These findings demonstrate that *Cd*MecA is transcriptionally regulated by the *CdmecI-CdmecR* locus, enabling cefotetan-responsive sporulation and revealing a tightly coordinated resistance mechanism.

We screened a global collection of 10,330 *C. difficile* genomes across 270 multilocus sequence types (STs) spanning all Clades^6^ to assess the distribution of *CdmecA, CdmecR* and *CdmecI*. *CdmecA* was present in 9.8% of genomes and 17.0% of STs (Supplementary Notes 2, Supplementary Data 1, Supplementary Tables 4 and 5) with the highest prevalence in Clades 5 (55.5%), 4 (40.8%) and 1 (4.7%) (Supplementary Tables 4). Within these clades, the prevalence was the highest in STs 3 (46.9%), 4 (57.9%), 11 (56.2%), 37 (35.8%), and 81 (100.0%), all of which were known epidemic lineages in humans^15–18^. Notably, *CdmecI* and *CdmecR* were detected across all Clades, even in strains lacking *CdmecA* (86.8%; Supplementary Table 4, Supplementary Data 1, Supplementary Notes 2), suggesting a conserved, broad regulatory framework. These findings highlight the widespread presence of *CdmecA* and its regulators, underscoring their potential role in strain-specific resistance and sporulation dynamics.

### *CdmecA* resistance to cephamycin-mediated sporulation inhibition extends to other spore formers

All spore-forming bacteria encode a SpoVD homologue^19^, essential for spore peptidoglycan synthesis. We previously showed that cephamycins inhibit sporulation in four species by binding SpoVD^8^. Given *Cd*MecA’s ability to bypass SpoVD inhibition in *C*. *difficile*, we hypothesised that similar mechanisms may exist in other spore-formers carrying *mecA*. This is highly plausible due to the ecological overlap among environmental, gut, and clinical spore formers, ecosystems that facilitate horizontal gene transfer and the spread of resistance genes.

**Fig. 2:**
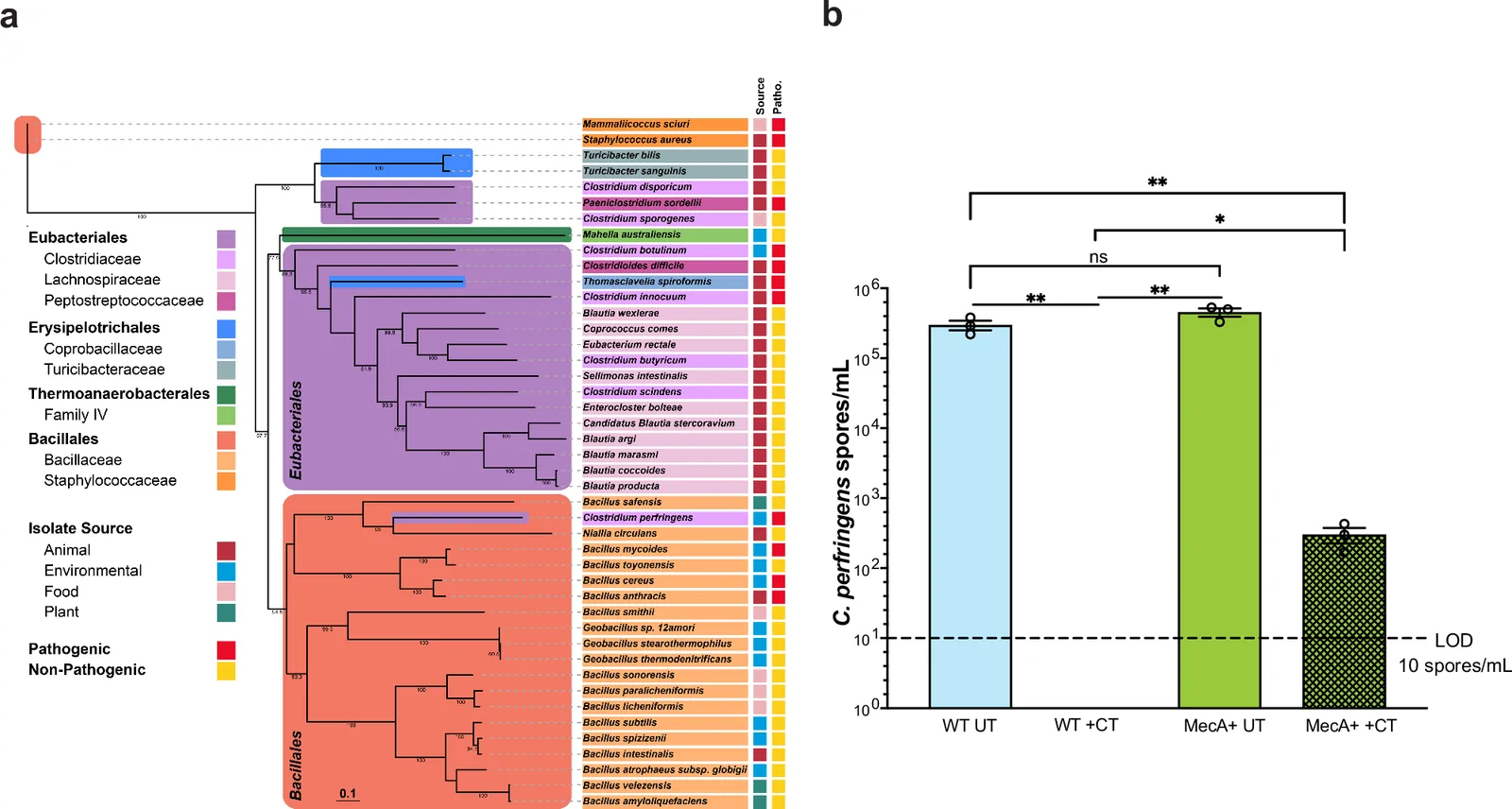
MecA distribution in spore-former pathogens, gut microbes, and environmental isolates and the effect of *Cd*MecA on cefotetan-mediated inhibition of *C. perfringens* sporulation. **a** On the left, the maximum likelihood phylogenetic relationship of *mecA* was inferred with the GTR+G4+I nucleotide substitution model. The tree was rooted against the non-spore-forming *M. sciuri*. The leftmost vertical bar represents isolate source, and the rightmost represents pathogenicity. Values above branches represent support levels from 1000 bootstraps. **b** *C. perfringens* DLL6642 spore numbers were determined from untreated (UT) and cefotetan-treated (+CT) wild-type and *Cd*MecA^*+*^ cultures. Results are the average of three independent biological replicates. Error bars represent s.e.m.; *P*-values were determined by a two-tailed Student’s *t*-test. **P = 0.0030, 0.0019, 0.0031, 0.0019, *P = 0.0162. Sporulation was abolished in the cefotetan-treated wild-type strain but was able to be partially restored in trans by *CdmecA*^*+*^. Limits of detection (LOD) are depicted as a horizontal dotted line. Source data are provided as a Source Data file.

To assess *mecA* prevalence among environmental and gut spore formers, we screened the NCBI database and identified 42 strains across 40 species with genes showing 26–35.6% nucleotide and 30.5–48.6% amino acid identity to *Cd*MecA. AlphaFold3 modelling of the 42 *mecA* genes revealed highly conserved MecA and transpeptidase domain folds across all models, with average RMSDs of 1.9 Å and 1.6 Å, respectively, supporting their classification as MecA homologues, with representative structures shown in Supplementary Fig. 4. The species span four spore-forming bacterial orders. A maximum likelihood phylogenetic tree, rooted against the non-spore-former *Mammaliicoccus sciuri* revealed widespread distribution of *mecA*, suggesting possible horizontal gene transfer between ecologically diverse species (Fig. 2a, Supplementary Fig. 5, Supplementary Notes 3) and highlighting the potential for MecA-mediated resistance to extend across environmental, gut, and clinical niches.

To test whether the *Cd*MecA resistance mechanism applies to other spore formers, we introduced *Cd*MecA into a non-MecA *C. perfringens*, an organism common in gut and environmental microbiota. Sporulation assays showed that cefotetan inhibited sporulation in the wild-type strain (Fig. 2b, Supplementary Fig. 1f). However, in the *Cd*MecA-expressing strain, sporulation was partially restored despite cefotetan treatment (Fig. 2b), confirming that *Cd*MecA can counteract cephamycin-induced sporulation inhibition. These findings suggest that *mecA*-driven resistance is not limited to *C*. *difficile* but may extend across diverse spore-forming pathogens, reshaping survival strategies under antibiotic pressure.

### Cefotetan-induced *Cd*MecA spores differ phenotypically compared to wild-type *Cd*SpoVD spores

**Fig. 3:**
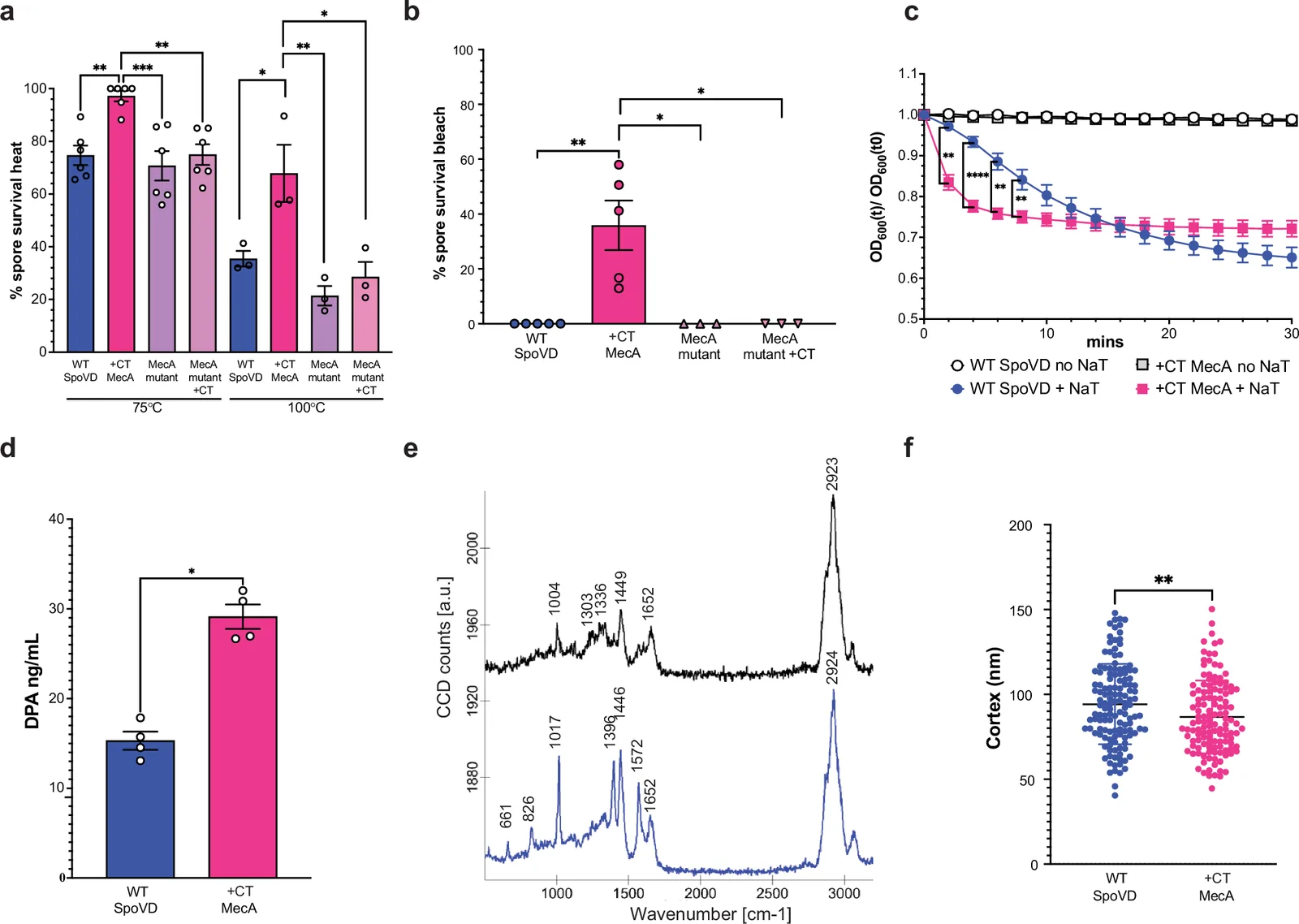
Cefotetan-induced *Cd*MecA spores have altered spore properties compared to wild-type *Cd*SpoVD spores. **a**, **b** WT *Cd*SpoVD or +CT *Cd*MecA spores were left untreated or treated with 75 °C or 100 °C heat, or bleach, after which spores were incubated on HIST agar to determine the percentage of spore survival. Results are the average of three to six independent *Cd*SpoVD and *Cd*MecA spore samples. Error bars represent s.e.m. *P*-values were determined by a one-way ANOVA test. **P = 0.0038, ***P = 0.0008, **P = 0.0043; *P = 0.039, **P = 0.0047, **P = 0.0124; *P = 0.00797, *P = 0.0357. **c** Germination rates of WT *Cd*SpoVD or +CT *Cd*MecA spores in the presence of 50 μM sodium taurocholate. Spores incubated in the absence of sodium taurocholate were included as a negative germination control. Data represents the ratio of the OD_600_ at each time point (OD_600_(t)) over the OD_600_ at time 0 (OD_600_(t0)) and is measured over time (min). Statistical analysis was determined using a 2-way Anova with Tukey’s multiple comparisons test and showed a significant difference within the first 8 min of germination (**P = 0.0018, ****P < 0.0001, **P = 0.0026, *P = 0.0496) and a difference in overall germinate rate (****P < 0.0001) between WT *Cd*SpoVD versus +CT *Cd*MecA spores in the presence of germinant on six independent *Cd*SpoVD and *Cd*MecA spore samples. **d** Quantification of DPA levels in WT *Cd*SpoVD and +CT *Cd*MecA spores. Purified spores were normalised to OD_600_ = 1.0 and boiled to release DPA. DPA in the supernatant was mixed with TbCl_3_ and detected by fluorimetry. Error bars represent s.e.m. *P*-value determined by two-tailed Student's *t*-test from four independent spore preps (*P = 0.0419). **e** Two distinct spectral profiles were found in Raman spectra of *Cd*MecA and *Cd*SpoVD spores: Ca-DPA-rich (blue line, typical for cefotetan-induced *Cd*MecA) and Ca-DPA-absent (black line, typical for WT *Cd*SpoVD). The Ca-DPA-rich profile was found in 43/52 *Cd*MecA spores. The Raman spectra are presented in the range of 400 –3200 cm^−1^ with prominent bands labelled. **f** Cortex size was measured between *Cd*SpoVD or *Cd*MecA spores. Statistical analysis was determined using a two-tailed Mann–Whitney test (*P* = 0.0078), rank-biserial correlation (*r*) ~ −0.191 corresponding to a small-to-medium size effect. Error bars represent s.e.m. Data are from six independent replicates of WT *Cd*SpoVD (*N* = 132) spores and six independent replicates of +CT *Cd*MecA (*N* = 128) spores. WT=wild-type, +CT=cefotetan-induced. Source data are provided as a Source Data file.

We hypothesised that spores formed via *Cd*SpoVD (in the absence of cephamycins) and those formed via *Cd*MecA (in the presence of cephamycins) may differ functionally. To test this, purified JGS6133 spores, either wild-type *Cd*SpoVD-generated or cefotetan-induced *Cd*MecA-generated, were exposed to heat and bleach stress, noting that hospital laundry is typically done at 65 °C for 10 min or 71 °C for 3 min. *Cd*MecA spores showed significantly greater resilience: 97% survived 75 °C for 30 min versus 75% of *Cd*SpoVD spores; at 100 °C, survival was 51% versus 22% (Fig. 3a; *P* = 0.0038, *P* = 0.039). Bleach treatment confirmed this, with 36% of *Cd*MecA spores surviving versus 0% of *Cd*SpoVD spores (Fig. 3b; *P* = 0.0079). *CdmecA* mutant spores, cefotetan-treated or untreated, resembled wild-type *Cd*SpoVD spores (Fig. 3a, b). Germination, a key function of spores and critical to infection, was also examined in the presence of the germinant taurocholate, revealing that *Cd*MecA spores germinated faster, showing a sharper OD drop within 8 min (Fig. 3c). These results suggest *Cd*MecA spores are more robust and environmentally persistent, with enhanced germination potential, key traits for transmission and infection.

Given the distinct resistance and germination profiles of *Cd*SpoVD and *Cd*MecA spores, we investigated differences in calcium dipicolinate (Ca-DPA), a key spore component linked to stress resistance and germination. In *C*. *difficile*, cortex hydrolysis precedes Ca-DPA release during germination^20^. DPA quantification assays showed significantly higher release from *Cd*MecA spores compared to *Cd*SpoVD spores (*P* = 0.0419; Fig. 3d). To validate this, Raman spectroscopy was used to assess single-spore composition. Ca-DPA-specific bands were detected in 43/52 *Cd*MecA spores, but only 1/52 *Cd*SpoVD spores (Fig. 3e, Supplementary Notes 4). Unlike the DPA assays, Raman spectra are acquired from individual spores and are intended as a qualitative comparison. These findings suggest that cefotetan-induced *Cd*MecA expression produces spores with elevated Ca-DPA levels, potentially enhancing resistance and accelerating germination.

### *Cd*MecA alters spore cortex size, peptidoglycan structure and proteome

*C. difficile* spores contain multiple layers, including a thick peptidoglycan-rich cortex. To determine whether the differences between wild-type *Cd*SpoVD and cefotetan-induced *Cd*MecA spores were linked to cortex structure, we performed TEM imaging and measured cortex thickness. *Cd*MecA spores showed a statistically significant 8% reduction in cortex size compared to *Cd*SpoVD spores (*P* = 0.0078; Fig. 3f, Supplementary Fig. 6). These structural changes may contribute to the enhanced phenotypes described above.

*Cd*SpoVD and *Cd*MecA are class B PBPs transpeptidases that catalyse peptide cross-linking during peptidoglycan synthesis. To compare their impact, we analysed spore peptidoglycan composition using LC-MS and PGFinder (Supplementary Data 2, 3)^21,22^. High-resolution profiling was carried out on spore peptidoglycan from cultures exposed to cefotetan or not. This treatment specifically inhibits *Cd*SpoVD and therefore reveals the specific contribution of *Cd*MecA to PG structure. Exposure to cefotetan revealed minimal structural differences, with similar cross-linking indices (3.85% ± 0.41% versus 3.90% ± 0.43% in the presence of cefotetan; *P* > 0.05; Supplementary Data 4, Supplementary Notes 5), indicating overall cross-linking and proportion of 4–3 crosslinks remains unchanged. Only one low-abundance trimeric muropeptide showed a five-fold change in abundance (Supplementary Fig. 7). While these differences may influence spore phenotype, they cannot be directly attributed to *Cd*MecA.

To examine if the spore proteome differed between *Cd*SpoVD and *Cd*MecA spores, quantitative proteomic analysis was performed. The analysis revealed significant differences in protein abundance (Supplementary Fig. 8 and Supplementary Data 5). *Cd*SpoVD spores showed elevated abundance of proteins linked to β-lactam resistance (MecI, 4.22-fold), undefined surface proteins (LytC_18; 2.35-fold; LytC_24; 3.15-fold), toxins (TcdA; 1.89-fold; TcdB; 1.76-fold), and the phosphotransferase system (PTS sugar transporter subunit IIB, 2.49-fold). In contrast, *Cd*MecA spores had proteins linked to β-lactam resistance protein *Cd*MecA (4.94-fold) and carbohydrate transporter PbuX (2.15-fold). The reciprocal enrichment of *Cd*MecI in* Cd*SpoVD spores and *Cd*MecA in *Cd*MecA spores independently supports their respective roles in cephamycin response and highlights distinct functional profiles between these spore populations.

### *Cd*SpoVE and *Cd*SpoVD are required for *Cd*MecA cephamycin-resistance activity

**Fig. 4:**
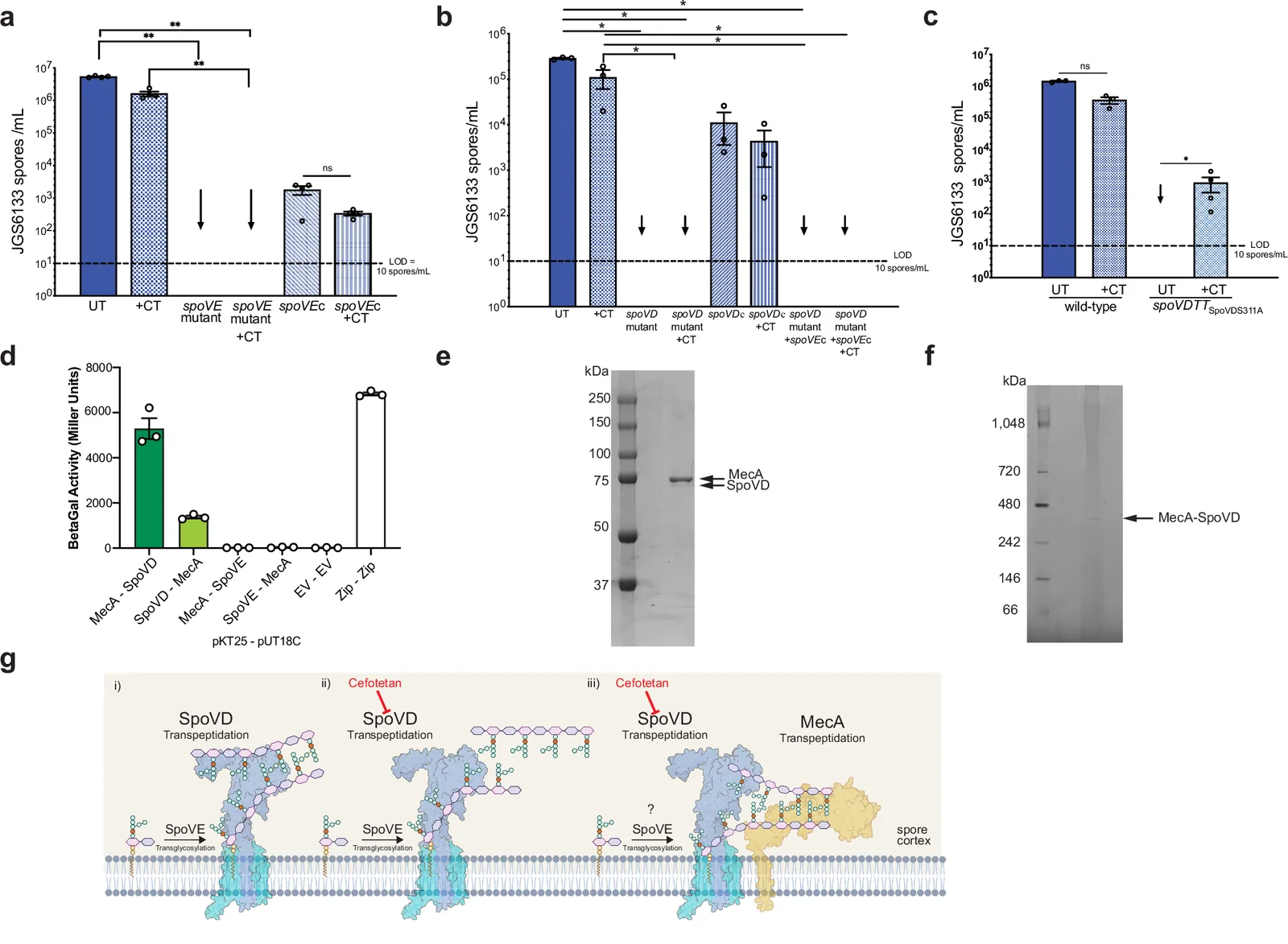
*Cd*SpoVE and *Cd*SpoVD contribute to *Cd*MecA’s role in continuing sporulation in the presence of cephamycins. **a** JGS6133 spore numbers were determined from untreated and cefotetan-treated wild-type, *CdspoVE* mutant and *CdspoVEc* cultures. Statistical significance was observed between untreated wild-type and untreated *spoVE* mutant (**P = 0.0045), untreated wild-type and cefotetan-treated *spoVE* mutant (**P = 0.0045) and cefotetan-treated wild-type and cefotetan-treated *spoVE* mutant (**P = 0.0043). No statistical significance was found between untreated and cefotetan-treated wild-type (*P* = 0.6132) and *spoVEc* (*P* = 0.6836). **b** JGS6133 spore numbers were determined from untreated and cefotetan-treated wild-type, *CdspoV*D mutant, *CdspoVDc* and *spoVD* mutant +*spoVE*c. Statistical significance observed between untreated wild-type and untreated *spoVD* mutant (*P = 0.0158), untreated *spoVD* mutant+ *spoVEc* (*P = 0.0158) cefotetan-treated *spoVD* mutant+ *spoVEc* (*P = 0.0158); cefotetan-treated wild-type and cefotetan-treated *spoVD* mutant, (*P = 0.0444) untreated *spoVD* mutant +*spoVEc* (*P = 0.0296) and cefotetan-treated *spoVD* mutant +*spoVEc* (*P = 0.0296). No statistical significance was observed between untreated and cefotetan-treated wild-type (*P* > 0.9999) and complemented *spoVD* mutant strains *(P* > 0.9999). **c** JGS6133 spore numbers were determined between untreated and cefotetan-treated wild-type and *Cd*SpoVDTT_SpoVDS311A_ cultures. Sporulation was partially restored in cefotetan-treated *Cd*SpoVDTT_SpoVDS311A_, with a statistically significant difference observed between untreated and cefotetan-treated cultures (*P = 0.0286). No statistical significance was observed between untreated and cefotetan-treated wild-type (*P* = 0.1000). **d** Bacterial two-hybrid analyses of interactions between *Cd*MecA-SpoVD and *Cd*MecA-*Cd*SpoVE. The β-galactosidase activity was normalised to the negative control. **e** HIS-tagged *Cd*SpoVD (70 kDa) co-elutes with Strep-II-tagged *Cd*MecA (75 kDa) when purified by Strep affinity chromatography. **f** Co-purified *Cd*SpoVD and *Cd*MecA resolve as a single band on blue native PAGE with a size shift greater than monomeric *Cd*SpoVD and *Cd*MecA. **g** Proposed model of *Cd*MecA mechanism of action. (i) In the absence of cephamycins, spore peptidoglycan synthesis proceeds with the *Cd*SpoVE (SEDS protein, glycosyltransferase)-*Cd*SpoVD (Class B PBP, transpeptidase) complex, enabling sporulation. (ii) Cephamycins target *Cd*SpoVD, disrupting the *Cd*SpoVE*-Cd*SpoVD complex, inhibiting spore peptidoglycan synthesis and thus sporulation. (iii) In a *Cd*MecA strain, when cephamycins are present, *Cd*MecA interacts with the *Cd*SpoVE-catalytically inactive *Cd*SpoVD complex, enabling spore peptidoglycan synthesis to continue, rescuing sporulation (created in BioRender (2026) https://BioRender.com/o06lrle). UT=untreated, +CT=cefotetan-treated, *spoVE*c=*spoVE* complemented, *spoVD*c=*spoVD* complemented, *Cd*SpoVDTT_SpoVDS311A_ =SpoVD mutant complemented in trans with catalytically inactive SpoVDS311A. Sporulation assay results are the average of three to four independent biological replicates. Error bars represent s.e.m.; *P*-values were determined using a Kruskal–Wallis test with Dunn’s pairwise comparisons (**a**–**c**) or a two-tailed Mann–Whitney test (**d**). Limit of detection (LOD) shown as a horizontal dotted line. Source data are provided as a Source Data file.

PBP2a (MecA) from *S. aureus* and *Cd*MecA are class B PBPs, and both function as transpeptidases in the presence of specific β-lactams. PBP2a in *S. aureus* requires the native class A bifunctional PBP2 glycosyltransferase to synthesise vegetative cell peptidoglycan under β-lactam pressure^23^. Similarly, *Cd*MecA depends on a glycosyltransferase partner to maintain spore peptidoglycan synthesis in the presence of cephamycins. One candidate is the SEDS protein SpoVE, which pairs with SpoVD to form the core peptidoglycan synthase complex^12,24,25^. We hypothesised that when *Cd*SpoVD is inhibited by cefotetan, *Cd*SpoVE cooperates with *Cd*MecA to sustain spore peptidoglycan production. Sporulation assays revealed that a *CdspoVE* mutant failed to sporulate, even with cefotetan, but sporulation was partially restored by in trans complementation (Fig. 4a, Supplementary Fig. 9a, b). These results indicate that *Cd*MecA activity during sporulation depends on *Cd*SpoVE.

In *B. subtilis*, SpoVE relies on SpoVD for activity and stability^26^, suggesting that *Cd*SpoVD may be essential for peptidoglycan synthesis during sporulation alongside SpoVE and *Cd*MecA under cephamycin pressure. Sporulation assays with cefotetan showed that a *CdspoVD* mutant was severely impaired in spore formation, which was partially rescued by in trans complementation (Fig. 4b, Supplementary Fig. 9c). PBP profiling confirmed *Cd*MecA induction in the cefotetan-treated *CdspoVD* mutant, indicating that *Cd*MecA expression is independent of *Cd*SpoVD (Supplementary Fig. 9d). However, *Cd*MecA alone was insufficient to restore sporulation, highlighting the requirement for *Cd*SpoVD in peptidoglycan synthesis mediated by *Cd*SpoVE and *Cd*MecA (Fig. 4b).

In non-spore formers like *S. aureus* and *Enterococcus faecalis*, FtsW (a SEDS homologue of SpoVE) remains active even when its cognate class B PBP is catalytically inactive^27,28^. Cephamycins inhibit transpeptidase PBPs by binding their active-site serine, blocking enzymatic function. To test whether a catalytically inactive *Cd*SpoVD could still support *Cd*MecA-mediated sporulation, we introduced a *Cd*SpoVD mutant (S311A), in which the active-site serine was replaced with alanine^29^, into the *CdspoVD* mutant (*CdspoVDTT*_SpoVDS311A_). Sporulation was partially restored in the cefotetan-treated mutant (Fig. 4c, Supplementary Fig. 9e), indicating that *Cd*SpoVD supports *Cd*MecA function independently of its catalytic activity. This was reinforced by bacterial two-hybrid assays showing strong interaction between *Cd*SpoVD and *Cd*MecA (high β -galactosidase activity), but weak interaction between *Cd*SpoVE and *Cd*MecA (Fig. 4d).

Together, these findings suggest *Cd*SpoVD serves as a structural scaffold for *Cd*SpoVE activity by interacting with *Cd*MecA. Affinity chromatography showed that *Cd*MecA and *Cd*SpoVD co-elute when co-expressed via the *Cd*MecA Strep-II tag (Fig. 4e, Supplementary Fig. 9f, g, Supplementary Notes 6), indicating a direct protein-protein interaction. Blue native PAGE of the co-purified complex revealed a single band (Fig. 4f), and mass spectrometry confirmed the presence of both *Cd*SpoVD and *Cd*MecA (Supplementary Data 6, 7). Collectively, these data demonstrate a direct interaction between *Cd*MecA and *Cd*SpoVD and suggest that *Cd*MecA requires both *Cd*SpoVE and *Cd*SpoVD to sustain sporulation under cephamycin pressure. We propose that cephamycins inhibit *Cd*SpoVD’s transpeptidase activity, while *Cd*SpoVE retains glycosyltransferase function (Fig. 4gi-ii). In *C. difficile* strains carrying *CdmecA*, *Cd*MecA interacts directly with catalytically inactive *Cd*SpoVD to restore transpeptidase activity within the SpoVD/SpoVE complex (Fig. 4g iii). However, further experimental validation is required to substantiate this model.

### Ceftobiprole targets *Cd*MecA, reducing sporulation in co-treatment with cefotetan

We previously showed that combining cefotetan with vancomycin prevented recurrent CDI with strains lacking *Cd*MecA^8^. However, cefotetan alone is ineffective against *Cd*MecA-carrying strains. To broaden anti-sporulation strategies, we explored a dual therapy: cefotetan, which targets *Cd*SpoVD^8^, and ceftobiprole and ceftaroline, fifth-generation cephalosporins that bind to the active sites of *mecA*-encoded PBPs, bypassing β-lactam resistance^30,31^.

**Fig. 5:**
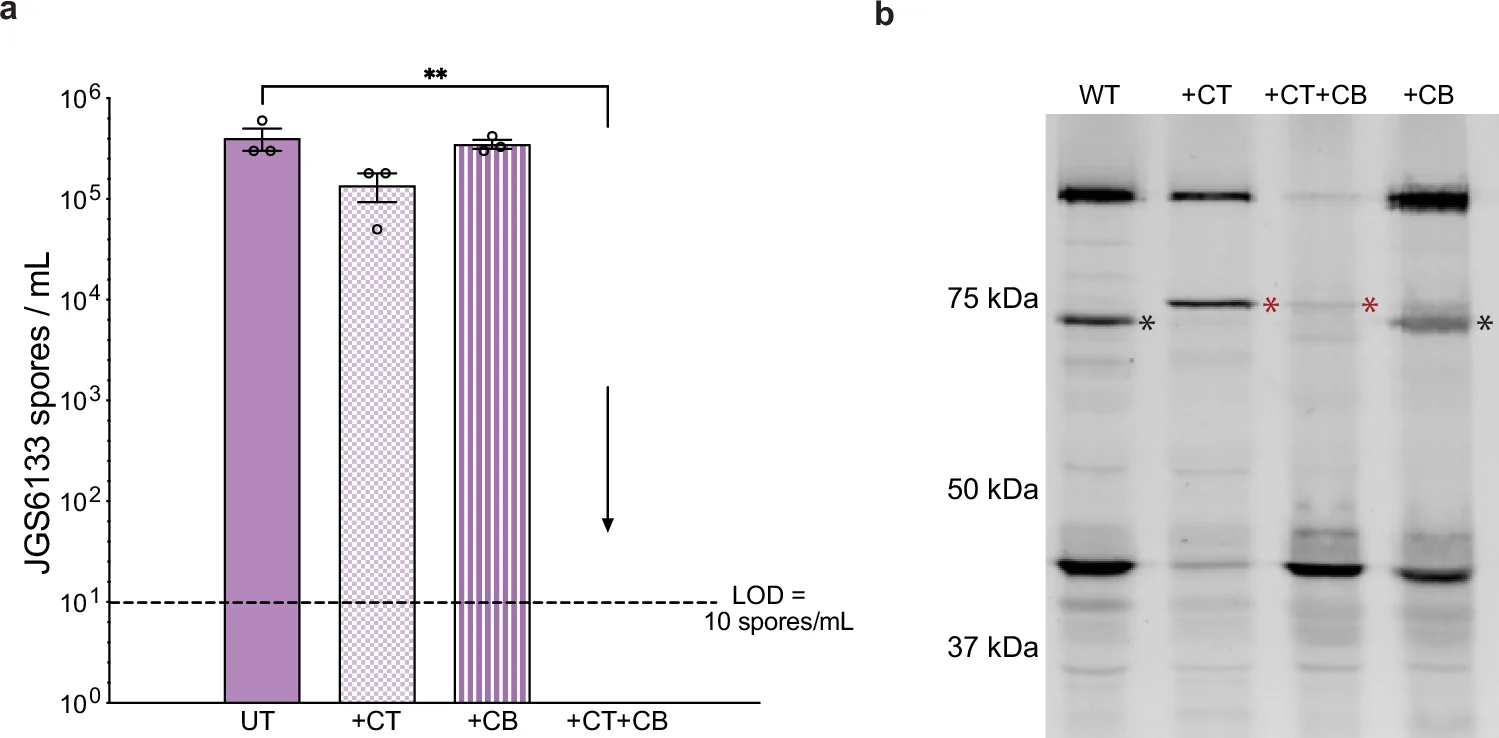
Co-treatment with cefotetan and ceftobiprole abolishes sporulation in *C. difficile* JGS6133 and targets cefotetan-induced *Cd*MecA. **a** Spore numbers were determined from three independent cultures of strain JGS6133: untreated and cefotetan-treated wild-type, ceftobiprole-treated wild-type and wild-type co-treated with cefotetan and ceftobiprole. Results are the average of three independent biological replicates. Error bars represent s.e.m.; *P*-values were determined using a Kruskal–Wallis test with Dunn’s pairwise comparisons. Co-administration of cefotetan and ceftobiprole significantly reduced spore numbers compared to untreated cultures (**P = 0.0066). No significant differences in spore numbers were seen on any day between untreated cultures and cultures treated with cefotetan only (*P* = 0.636) and ceftobiprole only (*P* = 0.2087). Limit of detection (LOD) shown as a horizontal dotted line. **b** Membrane proteins from sporulating wild-type JGS6133 untreated cultures, cefotetan-treated cultures, cultures co-treated with cefotetan and ceftobiprole or treated with ceftobiprole only were isolated, incubated with Boc-FL and separated by SDS–PAGE. Labelled PBPs were visualised using a FluorImager. The black asterisks indicate *Cd*SpoVD (73 kDa), and the red asterisks indicate *Cd*MecA (76 kDa). UT=untreated, +CT=cefotetan, +CT+CB=cefotetan and ceftobiprole; +CB=ceftobiprole. Source data are provided as a Source Data file.

JGS6133 cultures were treated with a single sub-MIC dose of cefotetan, ceftobiprole, ceftaroline or co-treatment of each cephalosporin with cefotetan. Individually, neither antibiotic significantly reduced spore numbers. However, co-treatment completely abolished sporulation, suggesting a synergistic effect (Fig. 5a, Supplementary Fig. 11a). Vegetative cell counts remained unchanged, confirming that the effect was specific to sporulation (Supplementary Figs. 10 and 11b). PBP profiling confirmed the absence of *Cd*SpoVD and weak *Cd*MecA presence under combination treatment of ceftobiprole with cefotetan (Fig. 5b) or ceftaroline with cefotetan (Supplementary Fig. 11c), indicating that cefotetan targets *Cd*SpoVD^8^, while ceftobiprole and ceftaroline effectively inhibit *Cd*MecA.

## Discussion

Spore-forming bacteria are key contributors to the evolution, transmission, and persistence of antibiotic resistance across One-Health ecosystems^32^. This study identifies a new resistance pathway associated with sporulation, where *C. difficile* isolates acquire the accessory PBP *Cd*MecA, compensating for the functional loss of the core sporulation protein *Cd*SpoVD under cefotetan stress. This mechanism allows an antibiotic resistance gene to bypass sporulation-targeting antibiotics. Notably, this pathway also appears in *C*. *perfringens*, highlighting its potential conservation across spore-forming pathogens.

*Cd*MecA is the first known antibiotic resistance gene to confer resistance to both spores and, as shown in a separate study^33^, vegetative cells. While *Cd*MecA did not enhance cephamycin resistance in *C. difficile* vegetative cells, likely due to the species’ intrinsic resistance, it was previously linked to carbapenem resistance in RT017 clinical isolates carrying PBP5 (*Cd*MecA) via mutations in *pbp1* and *pbp3*^33^. These findings suggest that *CdmecA* provides a survival advantage to both cell types under β-lactam exposure. The work detailed here represents a new resistance pathway associated with sporulation. Notably, *Cd*MecA was found in ~10% of surveyed *C. difficile* genomes, predominantly in Clades 4 and 5, especially STs 11, 37, and 81^16,17,34^. These findings suggest that although *Cd*MecA is not currently associated with increased disease burden, its presence in emerging and environmentally prevalent clades supports the need for continued genomic surveillance. Given that over 50% of gut microbiota are spore formers^35^, spores may act as reservoirs for resistance genes, facilitating their spread to other commensals and pathogens^36,37^. The global rise of *S. aureus* PBP2a-associated methicillin resistance, representing the largest increase in attributable AMR burden globally^38,39^, underscores the potential impact of such mechanisms across ecosystems.

Acquisition of *CdmecA* may enhance the persistence and survival of *C. difficile* spores in both host environments and external settings^40^. *Cd*MecA-derived spores exhibited greater resilience than wild-type *Cd*SpoVD spores, potentially due to altered Ca-DPA levels, which are known to contribute to spore resistance^41^. These spores also had a thinner cortex, likely enabling faster germination by reducing the amount of cortex material requiring hydrolysis. Together, these findings show that *Cd*MecA-driven spores possess distinct functional traits compared to SpoVD-derived spores. This altered phenotype may influence spore-host interactions and disease outcomes, further supporting the importance of *Cd*MecA’s role in sporulation under antibiotic pressure. Peptidoglycan analysis revealed no significant differences in cross-linking between *Cd*MecA and wild-type *Cd*SpoVD spores, indicating that *Cd*MecA can fully substitute for *Cd*SpoVD function without compromising sporulation. These findings support the idea that an acquired antibiotic resistance gene, *CdmecA*, can replace a core sporulation-PBP when inactivated by antibiotics, allowing sporulation to proceed under stress.

Our data show that *Cd*MecA-mediated resistance to cephamycin-induced sporulation inhibition depends on *Cd*SpoVE and *Cd*SpoVD. *Cd*SpoVD likely acts as a structural scaffold, enabling *Cd*SpoVE to supply substrate for *Cd*MecA transpeptidase activity. This interaction was validated by bacterial two-hybrid assays, co-purification, and mass spectrometry, confirming direct binding between *Cd*MecA and *Cd*SpoVD. This mechanism parallels a model in the non-spore-former *E. faecalis* where Pbp4, despite low cephalosporin affinity, cooperates with a SEDS-class b PBP complex to maintain vegetative cell peptidoglycan synthesis under antibiotic stress^27^.

Sporulation assays showed that co-treatment with cefotetan and ceftobiprole abolished sporulation in a *Cd*MecA strain by disrupting both *Cd*SpoVD and *Cd*MecA function. Importantly, ceftobiprole has minimal impact on gut microbiota^42^ and does not induce *C. difficile* toxin production^43^, making it a promising candidate for CDI therapy. These findings provide proof-of-principle that targeted inhibition of PBP activity could form the basis for rational anti-sporulation strategies, with ceftobiprole offering a viable path forward.

In conclusion, we uncover a critical link between sporulation and antibiotic resistance, showing that resistance genes, previously thought to affect only vegetative cells, can also modulate sporulation and alter spore function. Our findings reveal a new resistance pathway associated with sporulation, highlighting how antibiotic overuse may unintentionally reshape spore biology and drive resistance in spore-forming bacteria. This has far-reaching implications for human and animal health, emphasising the need for targeted strategies to combat resistance in both cell types.

## Methods

### Bacterial strains and growth conditions

The characteristics and origins of recombinant strains and plasmids are shown in Supplementary Tables 1, 6 and 7. Bacteriological culture media were obtained from Oxoid. *C. difficile* strains were cultured in heart infusion supplemented with yeast extract, L-cysteine, and glucose (HIS) or TY medium unless otherwise stated, in an atmosphere of 10% H_2_, 10% CO_2_, and 80% N_2_ at 37 °C in a Coy chamber. *C. perfringens s*trains were cultured anaerobically as above in heart infusion (HI) supplemented with glucose, nutrient agar, fluid thioglycolate or Duncan and Strong Raffinose medium unless otherwise stated. *Escherichia coli* strains were cultured in 2×YT medium aerobically at 37 °C, with shaking for broth cultures. Antibiotics (Sigma-Aldrich or Focus Bioscience) were used at the following concentrations: D-cycloserine (250 µg/mL), cefotetan (5 µg/mL), ceftobiprole (5 µg/mL), thiamphenicol (10 µg/mL), 25 µg/mL chloramphenicol (*E. coli*), erythromycin (10 µg/mL), and lincomycin (50 µg/mL).

### *Clostridioides difficile* and *Clostridium perfringens* sporulation assays

*C. difficile* strains were grown overnight and adjusted to an OD_600_ of 0.1 in pre-reduced HIS broth and incubated until mid-exponential phase (OD_600_ 0.45–0.5). Once mid-exponential, 50 μL of OD_600_ 0.45–0.5 was added to 15 mL pre-reduced TY broth cultures. For cefotetan-treated cultures or ceftobiprole-treated cultures, or ceftoroline-treated cultures, sub-MIC concentrations (Supplementary Table 1) were added to TY broth cultures. Antibiotic concentrations were determined by minimum inhibitory concentration assays. *C. difficile* strains were analysed for growth against antibiotic concentrations ranging from 5 μg/mL to 100 μg/mL. The sub-inhibitory concentration was defined as the first well in which growth was observed (Supplementary Table 1). This ensured that only the impact of cefotetan or ceftobiprole on sporulation, and not vegetative cell growth, was tested. A 1 mL aliquot was used to determine vegetative cell and spore numbers. Half was used to perform 10-fold serial dilutions, with samples plated onto HIS agar containing 1% (w/v) sodium taurocholate (HIST, New Zealand Pharmaceuticals) to determine total viable cell counts (vegetative cells and spores). The other half of the 1 mL aliquot was heat-shocked at 65 °C for 30 min, 10-fold serial dilutions were performed, and samples were plated onto HIST to obtain spore numbers. Plates were incubated O/N anaerobically, at 37 °C. Vegetative cell numbers were obtained by subtracting spore counts from total cell counts. This was repeated for day 1. *C. perfringens* sporulation assays were performed exactly the same except for the following changes. *C. perfringens* DLL6642 was cultured anaerobically overnight in fluid thioglycolate medium. Pre-reduced Duncan and Strong Raffinose (DSR) broths supplemented with thiamphenicol (to maintain the MecIRA plasmid) were inoculated with 200 μL of overnight culture. For cefotetan-treated cultures, a sub-MIC concentration was added. Vegetative and spore numbers were determined by plating onto HI agar, with spores derived after treatment at 65 °C for 40 min.

At least three to six independent biological replicates were tested for each strain. Data were analysed using GraphPad Prism 10 and statistical significance assessed using the Kruskal–Wallis test with Dunn’s pairwise comparisons, the Mann–Whitney U test or two-tailed Student’s *t*-test. Note that biological replicates were performed on the same day.

### Labelling of *C. difficile* penicillin-binding proteins with Bocillin-FL for gel-based analysis

Bocillin-FL labelling and PBP analysis of *C. difficile* cells was performed as detailed in ref. ^8^. In brief, 1 ml of untreated or cephamycin-treated culture from the sporulation assay was collected by centrifugation (16,100 × *g* for 1 min at room temperature) and washed with 1 ml PBS pH 7.4. Cell pellets were resuspended in 50 μl PBS containing 5 μg ml^−1^ of Boc-FL (Thermo Fisher Scientific). After incubation for 10 min with Boc-FL at room temperature, the cells were washed and resuspended in 500 μl PBS containing 1 mg ml^−1^ of lysozyme and were incubated for 30 min at 37 °C. The cells were lysed using a Branson Sonifier 250 (power setting 3, 30% duty cycle for 5 × 6 s intervals), and the membrane proteome was isolated by centrifugation at 16,000 × *g* for 25 min at 4 °C. Membrane proteome was resuspended in 100 μl PBS, and protein concentration was determined using a NanoDrop 1000 Spectrophotometer (Thermo Scientific). Proteome samples were diluted to 2.5 mg ml^−1^ in PBS. Following the addition of 4× SDS–PAGE loading buffer to 40 μl of proteome sample, the sample was heated for 5 min at 90 °C, cooled to room temperature and run on a 10% SDS–PAGE gel. Labelled proteins were visualised in gel using a Typhoon Trio 76G58 gel scanner (Amersham Biosciences). The parameters used for scanning were 525 PMT, an Alexafluor 488 laser, with 100 μm resolution.

### Penicillin-binding protein identification

Bands of interest were separated by SDS–PAGE gel, excised, trypsin-digested, and subjected to tandem mass spectrometry at the Australian Proteome Analysis Facility, Macquarie University, Sydney, or the Monash Biomedical Proteomics Facility, Monash University, Melbourne. The corresponding penicillin-binding protein bands were determined by their presence or absence in untreated samples or cefotetan-treated samples.

### Screening for *CdmecA* in > 10,000 *C. difficile* genomes collection across 8 evolutionary Clades

To determine the distribution of *CdmecA* across the *C. difficile* species, SRST2 v0.2.0 [https://github.com/katholt/srst2] was used to screen for the presence of *CdmecA* in the paired-end reads of 10,330 publicly available *C. difficile* genomes encompassing 270 multilocus sequence types spanning *C. difficile* Clades 1–5 and cryptic Clades C-I, C-II, and C-III^6^. All *CdmecA-*positive genomes were assembled with SPAdes (v3.15.5) [https://github.com/ablab/spades] and annotated with Prokka v1.14.5 [https://github.com/tseemann/prokka]. To verify the presence and distribution of *CdmecA*, pan-genome analysis was performed on all assembled genomes using Panaroo (v1.1.0) [https://github.com/gtonkinhill/Panaroo] with default settings and *CdmecA* alleles from *CdmecA*-positive genomes were extracted and aligned to the reference *C. difficile CdmecA* strain JGS6133.

### Phylogenetic analysis of *mecA*

The *mecA* nucleotide sequences from 42 species were aligned using MUSCLE as implemented in the MSA R package (v1.32.0)^44^. The maximum likelihood tree was constructed with the Phangorn R package (v2.11.1). Model testing was performed in Phangorn, and the resulting GTR4+G4+I model was chosen according to the Bayesian information criterion (BIC) value. Bootstrap testing was performed with the standard Phangorn implementation based on 1000 replications and was rooted against the non-spore-forming organism *M. scirui*. The final tree was prepared with iTOL (v6)^45^ and Adobe Illustrator. MecA amino acid multiple sequence alignments were performed using Clustal Omega as implemented in the MSA R Package (v1.32.0) and visualised in iTOL (v6) with the chemistry colour scheme from the R ggmsa package^46^.

### AlphaFold modelling

*C. difficile* JGS6133 MecA_38-692_, MecR_246-599_ and MecI protein structures were predicted using AlphaFold2 via ColabFold v1.5.5 online server^47^ using default settings (Supplementary Fig. 12). Predicted structures with the highest confidence scores (Supplementary Table 2) were selected for further use (Supplementary Data 8). Resulting AlphaFold2 structure files were imported into ChimeraX^48^ along with published crystal structures of *S. aureus* MecA (PDB ID:1VQQ^49^), MecR (PDB ID: 6O9S^50^) and MecI (PDB ID: 1SD6^51^) taken from the Protein Data Bank. Structures were superimposed using the MatchMaker tool with default settings.

AlphaFold3^52^ was used to generate full-length models of representative MecA proteins from spore-former pathogens, gut microbes, and environmental isolates (Supplementary Fig. 12). Five models of each homologue were generated. Highest ranked models were assessed for their structural similarity to a *Cd*MecA model. MecA proteins were visualised and structurally aligned by their transpeptidase domain and MecA domain using ChimeraX^48^ MatchMaker.

### Construction of TargeTron mutants and in trans complementation strains

Gene target sequences of *CdmecA* (Cd_AI35_00437), *CdmecR* (Cd_AI35_00438), *CdmecI* (Cd_AI35_00439) and *CdspoVE* (Cd_AI35_02826) were identified by searching the annotated genome of *C. difficile* strain JGS6133. ClosTron.com was used to find TargeTron insertion sites and to design gene block fragments of 350 bps containing retargeted regions. TargeTron plasmids were constructed (Supplementary Table 6-7^53^), with some modifications. Fragments were digested with *Hin*dIII and *Bsr*GI, and the digested products were ligated into the pDLL46 vector backbone. The resulting plasmids, pDLL46-*CdMecA*, pDLL46-*CdMecR*, pDLL46-*CdMecI* and pDLL46-*CdspoVE* were introduced into the *E. coli* conjugative donor strain HB101. The resulting HB101 strains were used as the donor for the conjugative transfer of plasmid DNA into *C*. *difficile* strain JGS6133 and mutants were isolated as previously described^53^. The *CdspoVD* targetron mutant was previously constructed as detailed in ref. ^8^. Insertion of the intron into *C. difficile* JGS6133 *CdmecA, CdspoVE* and *CdspoVD* genes was confirmed by PCR using primer pairs *CdmecA*F1/*CdmecA*R1, *CdmecR*F1/*CdmecR*R1, *CdmecI*F1/*CdmecI*R1, *CdspoVE*F1/*CdspoVE*R1 and *CdspoVD*F1/ *CdspoVD*R1 (Supplementary Table 7).

The *CdmecA* and *CdspoVE* complementation plasmids were constructed by PCR amplifying the *CdmecAIR* and *CdspoVE* genes from JGS6133 using primer pairs *CdmecI*compF_*Sph*I/*CdmecA*compR_*Sac*I (*CdmecA*) and *CdspoVE*compF_ *Aat*II/*CdspoVE*compR_ *Sac*I (*CdspoVE*) (Supplementary Table 7). The resulting fragments were digested with *Sph*I or *Aat*II and *Sac*I and cloned into pMTL9361, resulting in the *CdmecAIR* or *CdspoVE* complementation plasmids. For *CdmecI* and *CdmecR* mutant complementation, PCR fragments containing the wild-type *CdmecI* and *CdmecR* genes were amplified by PCR using primer pairs *CdmecI*_comp_F_BamHI / *CdmecI*_comp_R _SacI and *CdmecR*_comp_F_BamHI/ *CdmecR*_comp_R_SacI, respectively (Supplementary Table 7). PCR fragments were digested using BamHI and SacI and cloned into the *E. coli-C. difficile* shuttle vector pRPF185^54^, where their expression is under the control of a tetracycline-inducible promoter. Generating *CdmecI* or *CdmecR* complementation plasmids. All complementation vectors were confirmed by nanopore sequencing (Plasmidsarus). *Intrans* complementation of the *CdmecA*, *CdmecI*, *CdmecR* and *Cd*s*poVE* mutants was achieved as detailed^53^. The *S311A* plasmid^29^ was introduced into the *C. difficile spoVD*TT mutant, and the *CdmecAIR* plasmid was introduced into *C. perfringens* DLL6642 by conjugative transfer from HB101 as detailed in ref. ^53^.

### Digital PCR

20 ng of cDNA was added to digital PCR (dPCR) master mix containing 2× SYBR green I dye, 4× Absolute Q DNA Digital PCR Master Mix (Applied Biosystems), appropriate primers and made up to 10 μL with DEPEC water. Nine microliters of the dPCR solution was loaded onto QuantStudio™ Absolute Q™ MAP16 Plates (Applied Biosystems) and topped with 15 μL QuantStudio™ Absolute Q™ Isolation Buffer (Applied Biosystems). The samples underwent digital PCR using a QuantStudio^TM^ Absolute Q^TM^ Digital PCR System (Applied Biosystems) with the following conditions: Preheating for 10 min at 96 °C followed by 40 cycles of denaturing for 5 s at 96 °C and annealing/extension for 15 s at 62 °C. As SYBR Green I dye was used, the FAM channel was selected for data collection. dPCR data was standardised to the amount of RNA added to each reaction, and gene expression was calculated as cDNA copies/ng.

### Recombinant protein expression and purification

The coding region corresponding to *Cd*SpoVD (residues 34–659) was PCR-amplified from M7404 genomic DNA and cloned into a pET22b vector with an in-frame C-terminal hexahistidine tag to generate *Cd*SpoVD_Δ1-34_^8^. *Cd*SpoVD_Δ1-34_ was transformed into *E. coli* C41 (DE3), transformed cells were grown overnight at 28 °C in autoinduction media supplemented with ampicillin. Codon-optimised *CdmecA*_*∆1-34*_; *CdmecA* and *CdspoVD* were synthesised by GenScript. *CdmecA*_*∆1-34*_ was provided in pET-24(+) (NcoI/XhoI). *CdspoVD* was cloned *Eco*RI/*Sal*I into pET-28a with a C-terminal hexahistidine tag, and *CdmecA* was cloned *Nco*I/*Xho*I into pBAD-30 to generate *Cd*SpoVD^His-6^ and *Cd*MecA^Strep-II^ constructs, respectively. Recombinant proteins were produced in *E. coli* BL21 (DE3) and grown at 37 °C with shaking in 2YT broth supplemented with kanamycin (*Cd*MecA_∆1-34_) or kanamycin and ampicillin for co-expression (*Cd*SpoVD^His-6^ and *Cd*MecA^Strep-II^). Protein production was induced when OD_600_ reached 0.6 by addition of 1 mM IPTG (*Cd*MecA_∆1-34_ and *Cd*SpoVD^His-6^) or 0.1% arabinose (*Cd*MecA^Strep-II^) and grown overnight at 30 °C with shaking (*Cd*MecA_∆1-34_) or 16 °C (*Cd*SpoVD^His-6^ and *Cd*MecA^Strep-II^).

Cell pellets containing *Cd*SpoVD_∆1-34_ or *Cd*MecA_∆1-34_ were resuspended in lysis buffer (1× PBS, 300 mM NaCl, 5% glycerol, 0.01% Triton X-100, 20 mM imidazole and ½ EDTA-free protease inhibitor tablet) and lysed by sonication. Proteins were purified via Ni-NTA-agarose chromatography (Wash buffer: 1× PBS, 300 mM NaCl, 5% glycerol, 20 mM imidazole; elution buffer: 1× PBS, 300 mM NaCl, 5% glycerol, 250 mM imidazole). Following Ni-NTA-agarose chromatography, elution fractions of interest were pooled and size exclusion chromatography was conducted using a Superdex 200 16/60 gel filtration column pre-equilibrated in 50 mM HEPES, 300 mM NaCl, 5% glycerol (*Cd*MecA_Δ1-34_) or 20 mM Tris-HCl pH 7.5, 0.15 M NaCl 5% glycerol (*Cd*SpoVD_Δ1-34_) using an Äkta Go FPLC (Cytiva). Fractions were analysed by SDS-PAGE, and fractions containing proteins of interest were pooled and concentrated to 5–15 mg mL^−1^.

Cell pellets containing co-expressed *Cd*SpoVD^His-6^ and *Cd*MecA^Strep-II^ proteins were resuspended in lysis buffer (20 mM HEPES pH 8, 200 mM NaCl, 20 mM MgCl2, 10 µg/mL DNase, 1 mM TCEP and ½ EDTA-free protease inhibitor tablet) and homogenised using a glass homogeniser. Cells were lysed by using a cell disrupter (Constant Systems). The lysate was then ultracentrifuged at 135,000 × *g* for 30 min at 4 °C, and the supernatant was removed. Membrane pellets were resuspended (20 mM HEPES pH 8, 500 mM NaCl, 10 µg/mL DNase, 1 mM TCEP) and ultracentrifuged at 135,000 × *g* for 30 min at 4 °C. Supernatant was removed, and membrane pellets resuspended in a solubilisation buffer (20 mM HEPES pH 8, 500 mM NaCl, 10 µg/mL DNase, 1 mM TCEP, 20% glycerol and 1% DDM). Membranes were incubated overnight at 4 °C with rotation, and solubilised membranes were centrifuged at 20,000 × *g* for 30 min at 4 °C. Lysate was applied to a 5 mL HisTrap (Cytiva) (Wash buffer: 20 mM HEPES pH 8, 500 mM NaCl, 10 µg/mL DNase 1 mM TCEP, 20% glycerol, 0.1% DDM and 20 mM imidazole, elution buffer: 20 mM HEPES pH 8, 500 mM NaCl, 10 µg/mL DNase 1 mM TCEP, 20% glycerol, 0.1% DDM and 250 mM imidazole) or loose Strep-Tactin Superflow resin (IBA Technology) (Wash buffer: 20 mM HEPES pH 8, 500 mM NaCl, 10 µg/mL DNase 1 mM TCEP, 20% glycerol, 0.1% DDM, elution buffer: 20 mM HEPES pH 8, 500 mM NaCl, 10 µg/mL DNase 1 mM TCEP, 20% glycerol, 0.1% DDM and 2.5 mM desthiobiotin). Elution fractions were assessed by SDS-PAGE, and fractions with protein of interest were pooled, concentrated, and visualised by SDS-PAGE, Western blot and blue native PAGE.

Blue native PAGE was performed using Native PAGE Novax Bis-Tris Gel System (Invitrogen). Native PAGE sample buffer (5×) and 5% G250 were added to samples already containing detergent (0.1% DDM) and loaded onto precast 4–16% Bis-Tris gels (Invitrogen). NativeMark (Invitrogen) was used for molecular weight standards. Gels were run using Native PAGE running buffer (1×) for the anode buffer and dark cathode buffer (1×) at 150 V for 2 h. Gels were fixed, stained with Coomassie R-250 and destained as per the manufacturer’s instructions.

### Fluorescence polarisation assays

The ability of cephamycin antibiotics to outcompete fluorescently labelled penicillin (Boc-FL) for binding to *Cd*MecA and *Cd*SpoVD was assessed by fluorescence polarisation assays using a spectrofluorimeter (PHERAstar FSX, BMG). The reactions were carried out in black 384–well plates, 50 μL total volume, in PBS pH 7.4, 0.01% Triton X-100 at ambient temperature. Assays contained 10 µM *Cd*MecA or 60 nM *Cd*SpoVD, 30 nM Boc-FL, and appropriate concentrations of cephamycins (established in 1:2 dilution series). A PBP was added to the cephamycins and incubated for 30 min. Boc-FL was then added, followed by an additional 30 min incubation prior to measuring fluorescence polarisation. Excitation and emission wavelengths of 490 and 520 nm respectively, and a focal height of 6.5 mm were used. The gains for the emission channels were adjusted on the no-antibiotic control wells of each PBP, so initial polarisation values were 7% of the detection limit. Fluorescence polarisation was calculated from parallel and perpendicular fluorescence intensities measured simultaneously; an average was calculated from five consecutive readings for each well. Each assay was performed in biological and technical duplicate. IC_50_ values for each of the cephamycin antibiotics were calculated using PRISM software (one-site binding) by plotting the relative fluorescence intensity against antibiotic concentration and presented ± the standard error.

### *Cd*MecA mechanism of action model

Full-length sequences of *Cd*SpoVD and *Cd*SpoVE were used to model a *Cd*SpoVD:*Cd*SpoVE complex with AlphaFold3 (Supplementary Fig. 12). Five models of the complex were generated using the AlphaFold3 server to predict the structure and interactions of *Cd*SpoVD and *Cd*SpoVE, predictions of the *Cd*SpoVD:*Cd*SpoVE complex were highly confident. *Cd*SpoVE is predicted to be made up of 10 TM helices, and the TM helix of *Cd*SpoVD is predicted to lie against TM helices 8 and 9 of *Cd*SpoVE. Based on the high confidence scores and the structural similarity to *E. coli* RodA-PBP2^55^, this modelled complex and interface are likely accurate. Full-length *Cd*MecA was also modelled using the AlphaFold3 server, and five models were generated. All models were evaluated based on pLDDT, pTM, ipTM, and PAE scores and compared to other known bPBP-SEDS structures.

### Spore purification

*C. difficile* spores were prepared by plating OD_600_ = 0.5 cultures onto TY agar plates supplemented with sodium thioglycolate and incubating them for 10 days at 37 °C in a Coy anaerobic chamber. Each plate was scraped, and growth was resuspended in 1 mL of H_2_0 and left at 4 °C for 2 days. Spores were harvested by centrifugation at 5000 × *g* for 5 min, supernatant discarded, and the pellet resuspended in 1 mL of Milli-Q water. This was repeated four times. Preparations were then combined into a 3 mL volume and layered on top of 10 mL 50% (w/v) Sucrose and centrifuged at 3400 × *g* for 20 min. Sucrose was removed, and the spore pellet was resuspended in 1 mL of H_2_0. The pellet was washed 4 times as detailed above and resuspended in 1 mL of H_2_0. Spores were visualised with a Gram stain. The colony-forming efficiency of the spores was compared by plating a standardised number of purified spores, determined by counting the number of germinated spores on HIST media, followed by growth at 37 °C. No differences in colony-forming efficiencies were observed between the strains.

### Spore resistance & germination

To determine spore resistance, 3–6 independent samples of 1 × 10^3^–1 × 10^6^ spores were treated with bleach (2.5% v/v) at room temperature for 10 min or heat at 75 °C for 30 min or 100 °C for 10 min. Percentage spore viability (the ability of spores to form colonies) following treatment was calculated as follows: (log_10_ CFU mL^−1^ after plating spores on HIST agar post-treatment/log_10_ CFU mL^−1^ after plating untreated spores on HIST agar) × 100.

Germination assays were performed as before^56^, comparing 2 × 10^7^ JGS6133 untreated spores (*Cd*SpoVD) or cefotetan-treated spores (*Cd*MecA). Data represent the mean of at least three biological replicates. Statistical analysis was performed with a 2-way analysis of variance (ANOVA) with Tukey’s multiple comparisons test.

### Raman spectroscopy

Spore samples of three biological replicates of *Cd*SpoVD and of *Cd*MecA spores were washed with Milli-Q water six times to ensure complete removal of residual media. Subsequently, the samples were diluted in 1 mL of Milli-Q water, mixed, and 1 µL of the solution was deposited on a Raman-grade CaF_2_ window and air-dried, resulting in individual distribution of spore cells. Raman spectra were collected using the WITec confocal CRM alpha 300 Raman microscope, with an air-cooled solid-state laser operating at 532 nm, 600 grooves per mm grating and a CCD detector, cooled to −60 °C. All spectra were recorded using a dry Olympus MPLAN (100×/0.90 NA) objective. The monochromator of the spectrometer was calibrated prior to measurements using the Raman scattering line produced by a silicon plate (520.5 cm^−1^). The laser power was set to 7 mW. Each spectrum was recorded with a spectral resolution of 3 cm^−1^ and in the spectral range of 0–3725 cm^−1^, with an integration time of 1 s and 50 accumulations per spectrum. Each spectrum was collected from a different single cell. A total of 43 spectra for *Cd*SpoVD and 53 for *Cd*MecA spores were collected.

### DPA quantification

The method was based on that described by Francis and Sorg^57^. Purified spores were normalised to an OD_600_ of 1 in 1 mL of ddH_2_O, and spore suspensions were incubated for 30 min at 100 °C to release DPA. After centrifugation at 10,000 × *g* for 5 min, 300 µL of supernatant was collected and mixed with 300 µL of 100 µM TbCl_3_. A volume of 150 µL of the mixture was transferred to a black, flat-bottom, 96-well plate, and the fluorescence signal was measured at 545 nm with excitation set at 272 nm using an Infinite M Plex plate reader (Tecan). Four independent samples were analysed in technical triplicate and compared with a standard curve to determine the DPA concentration.

### Transmission electron microscopy imaging and spore cortex size measurement

Bacterial spores were pelleted in 1.5 mL Eppendorf tubes at 12,000 rpm for three min and placed into primary fixative, consisting of 2.5% glutaraldehyde in 0.1 M sodium cacodylate buffer, for 2 h at RT. The cells were rinsed in fresh sodium cacodylate buffer three times for 15 min each. Secondary fixation was performed using 1% osmium tetroxide and 1.5% potassium ferricyanide in cacodylate buffer for 1 h at RT. The cells were rinsed in three washes of Milli-Q water for 15 min each. The fixed cell pellets were dehydrated by incubating in increasing concentrations of ethanol for 15 min, consisting of 30, 50, and 70% ethanol in distilled water. Cells were spun down, and the ethanol was replaced with two drops of 4% agarose and dispersed using a vortex mixer. The cells in agarose were allowed to cool for two hours to form a solidified agarose block. The agarose blocks were removed from the Eppendorf tubes and were dissected into cubes of 1 mm^3^. The cubes were then placed back into 70% ethanol for 1 h. Dehydration of the cubes was continued in 90% and 2 × 100% ethanol for 1 h each. The ethanol was removed and replaced with 100% propylene oxide for 1 h. Dehydrated cell cubes were incubated in a mixture of Epon resin and propylene oxide at a ratio of 1:1 for 6 h at room temperature, followed by a 2:1 Epon/propylene oxide mixture overnight. Cell cubes were incubated in 100% freshly made Epon resin for 6 h, followed by another 100% resin change overnight. The cell cubes were then placed into Beem capsules in 100% resin, and the resin polymerised for 48 h in an oven at 60 °C. Resin-embedded cell cubes were sectioned with a Diatome diamond knife using a Leica UCS ultramicrotome. Sections of thickness 70–90 nm were collected onto 100 mesh copper grids and stained sequentially with 1% uranyl acetate for 10 min and lead citrate for 5 min. The sections were imaged in a JEOL 1400+ transmission electron microscope at 80 kV, and images were captured with a digital camera at a resolution of 2 K × 2 K.

### Spore peptidoglycan extraction, digestion, and reduction

Peptidoglycan was extracted from purified spores using the following method^58^ in 1 mL volumes in microfuge tubes. All centrifugation steps were for 30 sec at 13,000 × *g*. Samples were prepared from four biological replicates. Spores were first decoated for 1 h at 37 °C in a solution containing 50 mM Tris-HCl [pH 8.0], 8 M urea, 1% SDS and 50 mM DTT. After five washes in water, spores were incubated in 5% (m/v) trichloroacetic acid at 95 °C for 6 min. Extracted spores were boiled in a solution containing 50 mM Tris-HCl (pH 8.0), 3% SDS, and 50 mM DTT for 20 min. Following four washes in Milli-Q water, the cortex peptidoglycan was resuspended in 245 µL of sodium phosphate buffer (pH 5.5) and digested with 250 U of mutanolysin for 16 h at 37 °C.

Muropeptides were recovered in supernatants following centrifugation and reduced after addition of 250 µL of 200 mM borate buffer (pH 9.0) and sodium borohydride (1 mg/mL). After 20 min at room temperature, the pH was adjusted to 5.0 with phosphoric acid.

### UHPLC Tandem mass spectrometry

An Ultimate 3000 Ultra High-Performance Chromatography (UHPLC; Dionex/Thermo Fisher Scientific) system coupled with a high-resolution Q Exactive Focus mass spectrometer (Thermo Fisher Scientific) was used for LC/HRMS analysis. Muropeptides were separated using a C18 analytical column (Hypersil Gold aQ, 1.9 µm particles, 150 × 2.1 mm; Thermo Fisher Scientific), equilibrated at a temperature of 50 °C. Muropeptide elution was performed by applying a mixture of solvent A (water, 0.1% [v/v] formic acid) and solvent B (acetonitrile, 0.1% [v/v] formic acid). LC conditions were acquired 0–15% B for 18 min, at a flow rate of 0.25 mL/min. The column was re-equilibrated for 10 min under the initial conditions.

The Orbitrap Exploris 240 was operated under electrospray ionisation (H-ESI high flow) in positive mode. Full scan (m/z 150–2250) at resolution 120,000 (FWHM) at *m/z* 200, with normalised AGC Target 100%, and automated maximum ion injection time (IT). Data-dependent MS/MS were acquired in a ‘Top 5’ data-dependent mode using the following parameters: resolution 30,000; AGC 100%, automated IT, with stepped collision energy 10.30%.

### Peptidoglycan structural analysis

LC-MS datasets were deconvoluted with the Byos® software v3.11 (Protein Metrics). Two sequential searches were carried out with PGFinder v1.4.0, with default settings (10 ppm tolerance, 0.5 min cleanup window) using the databases described in Supplementary Tables 2 and 3. Data from individual matching output was consolidated as previously described to calculate average intensities, retention times, observed monoisotopic masses, and ppm differences. Individual intensities for each replicate were compared using the open-source LFQ-Analyst software^59^. The files generated for the analysis (Protein_Groups.txt and Experimental_Design_Matrix.txt) are provided in Source Data. The analysis was performed using an adjusted *P*-value cutoff of 0.05, Benjamin–Hochberg FDR correction and single peptide identifications. Mass spectrum datasets available in the Glycopost repository (Accession number GPST000371).

### Proteomics sample preparation

The proteomics sample preparation utilised S-TRAP mini columns (Protifi: CO2-mini-80) as per the manufacturer’s instructions. 65 µg of *Cd*SpoVD and *Cd*MecA spores (three to four biological replicates) were solubilised in solubilisation buffer (5% SDS, 50 mM TEAB), prior to reduction and alkylation with 10 mM TCEP (ThermoFisher: catalogue# 77720) and 40 mM 2-Chloroacetamide (Sigma-Aldrich: catalogue# 79-07-2), respectively, at 55 °C for 15 min. Following reduction and alkylation, samples were acidified by adding phosphoric acid to a final concentration of 1.2% v/v. The samples were loaded onto S-TRAP columns, washed four times with 90% MeOH/ 10% 1 M triethylammonium bicarbonate (TEAB, catalogue: T7408-100ML), pH 7.55 and subsequently digested with mass spectrometry grade trypsin gold (Promega, catalogue: V5280) for 16 h, at 37 °C, at a ratio of 1:50 (trypsin:protein). Following digestion, peptides were eluted via stepwise elution with 80 µL 50 mM TEAB, followed by 0.2% formic acid (FA) and finally 50% ACN, 0.2% FA. Peptides were dried via speed-vacuum before being resuspended in 50 µL 2% ACN, 0.1% FA. Samples were acidified with 10 µL 10% FA, and a second desalting step was performed using in-house-generated C18 Stage-Tips^60^. Samples were dried and resuspended in 12 µL 2%ACN, 0.1% FA (v/v) containing iRT peptides (Biognosys, 1:100 ratio) for LC-MS/MS analysis.

### Mass spectrometry analysis

Samples were analysed with a Vanquish Neo HPLC coupled to an Orbitrap Astral mass spectrometer (Thermo Scientific) using a NanoSpray Flex source (Thermo Scientific) with a 50 cm µPAC^TM^ HPLC column (Thermo Scientific). Mobile phases A and B were 0.1% formic acid in water (Fisher Scientific, Optima LC-MS grade) and 0.1% formic acid/80% acetonitrile (Fisher Scientific, Optima LC-MS grade), respectively. The column was heated to 50 °C, and the flow rate was set to fast load (dependent on the pressure limitation of the column (700 nL/min and 450 bar)) at the start of the method to decrease delay time and turned to 350 nL/min at the start of the active gradient. Initial conditions of 4%B were ramped to 30%B from 0 to 45 min, then to 45%B for another 15 min. The column was washed for 5 min at 97.5%B and 700 nL/min at the end of the gradient, followed by fast equilibration on the Vanquish Neo LC with an upper pressure limit of 450 bar. For data-independent acquisition experiments on the Orbitrap Astral MS, MS1 spectra were collected at a resolving power of 240,000 over *m/z* 380–980. The MS1 normalised AGC target was set to 500% with a maximum injection time of 5 ms. DIA MS2 scans were acquired in the Astral analyser over a 380–980 m/z range with a normalised AGC target of 800%, a maximum injection time of 3 ms and a HCD collision energy setting of 25%, and a default charge state of +2. Window placement optimisation was turned on. Isolation widths of 2 Th and active gradient lengths of 60 min.

### Proteomics data analysis

Raw files were processed with Spectronaut v19.0 (240606.62635)^61^, using DirectDIA. Spectra were searched against the proteome assembly of JGS6133 (3644 entries) and the built-in universal contaminants (381 entries) and iRT peptides (1 entry). The assembled protein FASTA file for JGS6133 was mapped for homology to the UniProt reference proteome *C. difficile* strain 630 (*UP000001978, 3762 entries, accessed on 22/07/2024*) using pHMMER (HMMER v3.4)^62^ to enable gene-level analysis. Quantification was performed at the fragment level with channel interference removal enabled, a 1% false discovery rate was used for protein, peptide and peptide spectral match level identification and inbuilt normalisation was applied with a minimum of three fragment ions used for quantification. Tryptic digestion was used with specific cleavage rules and a missed cleavage tolerance of two. Carbamidomethylation of cysteines was set to a fixed modification while acetylation of n-termini and oxidation of methionine were set as variable modifications. Protein level abundance values were imported into the Monash Proteomics Analyst Suites *DIA-Analyst* tool (v.0.8.5)^59,63^ for statistical analysis and visualisation. Differential proteins were determined using an adjusted *p*-value of ≤0.05 and a log_2_ fold change of 0.75.

### Bacterial two-hybrid analyses

Bacterial adenylate cyclase two-hybrid (BACTH) assays were performed based on the system first described by Karimova et al.^64^. BTH101 *E. coli* cells were co-transformed with 100 ng of each BACTH assay vector and plated on LB agar plates supplemented with 50 µg/mL kanamycin, 100 µg/mL Ampicillin, and 0.5 mM isopropyl β-D-thiogalactopyranoside (IPTG). Plates were incubated at 30 °C for 62–64 h, and β-galactosidase activity was quantified as detailed in ref. ^65^.

### Reporting summary

Further information on research design is available in the Nature Portfolio Reporting Summary linked to this article.

## Supplementary information

Supporting Information

Description of Additional Supplementary Files

Supplementary Data 1

Supplementary Data 2

Supplementary Data 3

Supplementary Data 4

Supplementary Data 5

Supplementary Data 6

Supplementary Data 7

Supplementary Data 8

Reporting Summary

Transparent Peer Review file

## Source data

Source Data

## Data Availability

The data that support the findings of this study are available from the corresponding author upon request. Raw proteomics data (DIA-MS) files generated in this study for quantitative analysis and their Spectronaut-derived protein intensities have been deposited in the ProteomeXchange Consortium through the PRIDE partner repository with identifier PXD054316^66^. Source data are provided with this paper.
