# Supplementary material for: Uncovering an alternate pathway of antibiotic resistance in spore-forming bacteria
Source: Nat Commun. 2026 Jul 15;17:8674. doi: 10.1038/s41467-026-75594-5 (PMC13490422; doi:10.1038/s41467-026-75594-5)
Supplement: Supplementary file 1 [file 41467_2026_75594_MOESM11_ESM.pdf]

nature portfolio

Corresponding author(s): Dena Lyras

Last updated by author(s): Jun 9, 2026

Reporting Summary

Nature Portfolio wishes to improve the reproducibility of the work that we publish. This form provides structure for consistency and transparency in reporting. For further information on Nature Portfolio policies, see our Editorial Policies and the Editorial Policy Checklist.

Statistics

For all statistical analyses, confirm that the following items are present in the figure legend, table legend, main text, or Methods section.

n/a	Confirmed	
☐	☒	The exact sample size (*n*) for each experimental group/condition, given as a discrete number and unit of measurement
☐	☒	A statement on whether measurements were taken from distinct samples or whether the same sample was measured repeatedly
☐	☒	The statistical test(s) used AND whether they are one- or two-sided<br>*Only common tests should be described solely by name; describe more complex techniques in the Methods section.*
☒	☐	A description of all covariates tested
☐	☒	A description of any assumptions or corrections, such as tests of normality and adjustment for multiple comparisons
☐	☒	A full description of the statistical parameters including central tendency (e.g. means) or other basic estimates (e.g. regression coefficient) AND variation (e.g. standard deviation) or associated estimates of uncertainty (e.g. confidence intervals)
☐	☒	For null hypothesis testing, the test statistic (e.g. *F*, *t*, *r*) with confidence intervals, effect sizes, degrees of freedom and *P* value noted<br>*Give P values as exact values whenever suitable.*
☐	☒	For Bayesian analysis, information on the choice of priors and Markov chain Monte Carlo settings
☒	☐	For hierarchical and complex designs, identification of the appropriate level for tests and full reporting of outcomes
☒	☐	Estimates of effect sizes (e.g. Cohen's *d*, Pearson's *r*), indicating how they were calculated

Our web collection on statistics for biologists contains articles on many of the points above.

Software and code

Policy information about availability of computer code

Data collection	Data for fluorescence polarization assays was collected using a spectrofluorimeter (PHERAstar FSX, BMG).<br>Raman spectra were collected using the WITec confocal CRM alpha 300 Raman microscope, solid-state laser operating at 532 nm, 600 grooves per mm grating and a CCD detector. Spectra were recorded using a dry Olympus MPLAN (100×/0.90 NA) objective.<br>For mass spec analysis, samples were analysed with a Vanquish Neo HPLC coupled to an Orbitrap Astral mass spectrometer (using a NanoSpray Flex source with a 50 cm μPACTM HPLC column (Thermo Scientific). For data independent acquisition experiments on the Orbitrap Astral MS, MS1 spectra were collected at a resolving power of 240,000 over m/z 380-980. The MS1 normalized AGC target was set to 500% with a maximum injection time of 5 ms. DIA MS2 scans were acquired in the Astral analyser over a 380-980 m/z range with a normalized AGC target of 800% and a maximum injection time of 3 ms and a HCD collision energy setting of 25% and a default charge state of +2. Window placement optimization was turned on. Isolation widths of 2 Th and active gradient lengths of 60 min.
Data analysis	PRSIM version 10 was used to analyse data in this study.<br>For C. difficile CdmecA distribution, SRST2 v0.2.0 [https://github.com/katholt/srst2] was used paired-end reads of 10,330 publicly available C. difficile genomes All CdmecA-positive genomes were assembled with SPAdes (v3.15.5) [https://github.com/ablab/spades] and annotated with Prokka v1.14.5 [https://github.com/tseemann/prokka]. Pan-genome analysis was performed on all assembled genomes using Panaroo (v1.1.0) [https://github.com/gtonkinhill/Panaroo] with default settings and alleles from CdmecA-positive genomes extracted and aligned to reference CdmecA strain JGS6133.<br><br>For phylogenetic analysis of MecA in spore-formers, nucleotide sequences were aligned using MUSCLE as implemented in the MSA R package (v1.32.0). Maximum likelihood tree was constructed with the Phangorn R package (v2.11.1). Model testing was performed in Phangorn and resulting GTR4+G4+I model chosen according to Bayesian information criterion (BIC) value. Bootstrap testing was performed with standard

Phangorn implementation based on 1000 replications and rooted against M. scirui. Final tree was prepared with iTOL (v6) and Adobe Illustrator. Amino acid multiple sequence alignments were performed using Clustal Omega implemented in the MSA R Package (v1.32.0) and visualised in iTOL (v6) with the chemistry colour scheme from the R ggmsa package.

Protein structures were predicted using either AplphaFold3 or AlphaFold2 via ColabFold v1.5.5 online server using default settings. Full-length sequences of CdSpoVD and CdSpoVE model a CdSpoVD:CdSpoVE complex with AlphaFold3. Full length CdMecA was also modelled using the AlphaFold3 server. All models were evaluated based on pLDDT, pTM, ipTM, and PAE scores and compared to other known bPBP-SEDS structures. MecA homologues from spore-formers were modeled using AlphaFold3 and were superimposed against CdMecA. Models were visulaised in ChimeraX. AlphaFold2 structure files were imported into ChimeraX along with published crystal structures of S. aureus MecA, MecR and MecI (Protein Data Bank). Structures were superimposed using MatchMaker tool with default settings.

An Ultimate 3000 Ultra High-Performance Chromatography (UHPLC; Dionex/Thermo Fisher Scientific) system coupled with a high-resolution Q Exactive Focus mass spectrometer (Thermo Fisher Scientific) was used for LC/HRMS analysis. For PG structural analysis LC-MS datasets were deconvoluted with the Byos® software v3.11 (Protein Metrics). Two sequential searches were carried out with PGFinder v0.0.4, with default settings

For protein abundance data analysis raw files were processed with Spectronaut v19.0 (240606.62635), using DirectDIA. Spectra were searched against JGS6133 (3,644 entries) and in-built universal contaminants (381 entries) and iRT peptides (1 entry). JGS6133 FASTA file was mapped for homology to UniProt reference proteome C. difficile 630 using pHMMER (HMMER v3.4) 5 for gene level analysis. Protein level abundance values were imported into the Monash Proteomics Analyst Suites DIA-Analyst tool (v.0.8.5) 49,53 for statistical analysis and visualisation.

For manuscripts utilizing custom algorithms or software that are central to the research but not yet described in published literature, software must be made available to editors and reviewers. We strongly encourage code deposition in a community repository (e.g. GitHub). See the Nature Portfolio guidelines for submitting code & software for further information.

Data

Policy information about availability of data

All manuscripts must include a data availability statement. This statement should provide the following information, where applicable:

- Accession codes, unique identifiers, or web links for publicly available datasets
- A description of any restrictions on data availability
- For clinical datasets or third party data, please ensure that the statement adheres to our policy

The data that support the findings of this study are available from the corresponding author upon request. Raw proteomics data (DIA-MS) files generated in this study for quantitative analysis and their Spectronaut-derived protein intensities have been deposited in the ProteomeXchange Consortium through the PRIDE partner repository with identifier PXD054316 65. Source data are provided with this paper.

Research involving human participants, their data, or biological material

Policy information about studies with human participants or human data. See also policy information about sex, gender (identity/presentation), and sexual orientation and race, ethnicity and racism.

Reporting on sex and gender	N/A
Reporting on race, ethnicity, or other socially relevant groupings	N/A
Population characteristics	N/A
Recruitment	N/A
Ethics oversight	N/A

Note that full information on the approval of the study protocol must also be provided in the manuscript.

Field-specific reporting

Please select the one below that is the best fit for your research. If you are not sure, read the appropriate sections before making your selection.

☒ Life sciences ☐ Behavioural & social sciences ☐ Ecological, evolutionary & environmental sciences

For a reference copy of the document with all sections, see nature.com/documents/nr-reporting-summary-flat.pdf

Life sciences study design

All studies must disclose on these points even when the disclosure is negative.

Sample size	Sample sizes of 3-7 for in vitro assays sufficient for robust analysis based on our previous assays.
Data exclusions	No data pertaining to any sample shown in this study was excluded.
Replication	All assays were independently replicated at least three times unless otherwise indicated. Similar results were obtained across independent experiments, and all replication attempts were successful. For digital PCR(dPCR), the biological sample was taken from the sporulation assay, which was performed in triplicate. A single representative biological sample was used for dPCR due to time and resource constraints. Binding assays were performed in biological and technical duplicate.
Randomization	Not relevant to study. Groups were defined according to antibiotic dose with control such as wild-type untreated cultures used for sporulation assay.
Blinding	Blinding was not relevant to this study. For sporulation assays spore numbers were determined for each group and compared to an untreated control. For other assays comparisions were made between SpoVD and MecA spores.

Reporting for specific materials, systems and methods

We require information from authors about some types of materials, experimental systems and methods used in many studies. Here, indicate whether each material, system or method listed is relevant to your study. If you are not sure if a list item applies to your research, read the appropriate section before selecting a response.

Materials & experimental systems

n/a	Involved in the study	
☒	☐	Antibodies
☒	☐	Eukaryotic cell lines
☒	☐	Palaeontology and archaeology
☒	☐	Animals and other organisms
☒	☐	Clinical data
☒	☐	Dual use research of concern
☒	☐	Plants

Methods

n/a	Involved in the study	
☒	☐	ChIP-seq
☒	☐	Flow cytometry
☒	☐	MRI-based neuroimaging

Plants

Seed stocks	N/A
Novel plant genotypes	N/A
Authentication	N/A
