# Supplementary material for: Uncovering an alternate pathway of antibiotic resistance in spore-forming bacteria
Source: Nat Commun. 2026 Jul 15;17:8674. doi: 10.1038/s41467-026-75594-5 (PMC13490422; doi:10.1038/s41467-026-75594-5)
Supplement: Supplementary file 2 [file 41467_2026_75594_MOESM12_ESM.pdf]

nature portfolio

Peer Review File

Uncovering an alternate pathway of antibiotic resistance in spore-forming bacteria

Corresponding Author: Professor Dena Lyras

This file contains all reviewer reports in order by version, followed by all author rebuttals in order by version.

Version 0:

Reviewer comments:

Reviewer #1

(Remarks to the Author)
The manuscript by Srikhanta et al. describes the discovery of an MRSA mecA-related gene in C. difficile, which the authors suggest plays a antibiotic resistance role in C. difficile and possibly other spore-forming bacteria. The manuscript builds on the communicating author's previous work that cephamycin can inhibit sporulation in C. difficile by targeting the conserved spore-specific protein SpoVD, penicillin-binding protein. The author's discovery of mecA-related gene was guided by the observation that cephamycins lacked anti-sporulation activity against certain C. difficile. Here, the authors investigated the genetic and mechanistic basis underlying the clade-specific difference in the anti-sporulation effect of cephamycins. They identified CdMecA in Clade 4-5 and C-III that can functionally compensate for SpoVD inhibition by cephamycins to restore sporulation in the presence of cephamycin. The findings explained the lack of cephamycin anti-sporulation effect across isolates from different clades. The authors showed that ceftobiprole combined with cefotetan retained ability to inhibit sporulation, in otherwise cephamycin-resistant strains. While the authors suggest this has therapeutic potential, it seems unlikely that two beta-lactams will be given to control CDI to exacerbate dysbiosis, including disrupting firmicute population. No animal studies support this assertation by the authors. Overall, while the study provides interesting insights into a new clade-specific resistance pathway associated with sporulation in C. difficile, several aspects of the experimental rigor and data interpretation raise concerns. Major comments are below:

1) The study identified CdMecA in Clade 4-5 and C-III, this provides valuable insight into the genomic diversity of C. difficile. Clade 5 (ribotype 078) is associated with disease, and Clade 4 and cryptic clade C-III are rarely associated with disease. The current clinical significance remains unclear.
2) Figure 1d: It appears that introducing CdMecA into M7404 cells may not fully prevent the action of cefotetan. The impact in terms of log reduction is similar to that observed in figure 1a for M7404 exposed to cefotetan alone.
a. In this experiment, and maybe strain background, it appears CdMecA is not the only factor driving the phenotype.
b. It would be helpful if authors could discuss this possibility of the above, and provide additional data to clarify the extent to which CdMecA functionally compensates in the M7404 background.
c. Knowing the PBP profiles in M7404 would also be useful.
3) While TargeTron-based was used to inactivate cdmecR and cdmecI, two key control experiments are missing (see below, a, b); in the absence of these experiments, it is difficult to conclude with confidence that CdmecA is under regulatory control of MecR.
a. No complementation studies were not performed.
b. No expression studies(qPCR) to assess polar effects
4) In several experiments, key control data appears to be missing, this makes it difficult to fully interpret the results. Also, statistical comparison between key experimental conditions is often missing. For example, figure 1c is missing the complement untreated group. Figure 1d, no statical analysis between +MecA untreated and treated group was done. Other figures include: Figure 4b (SpoVD complement untreated) extended figure 1b and 1c (Untreated complement), extended figure 6c (spoVD complement UT), extended figure 1d (complement strains).
5) Line 127 to 134: the analysis is largely descriptive and lacks deeper interpretation. It would strengthen the manuscript if the authors could discuss the evolutionary or functional implications of this distribution. Also, it is intriguing that CdmecA is highly prevalent in more environmentally relevant clades but not clinically common clades. Could the authors comment on this?
6) There is concern regarding clinical utility of using a combination of cefotetan and ceftobiprole in terms of damage to the microbiota. Animal efficacy and microbiome experiments will be needed to support the claim for therapeutic utility by the

authors.
7) At several places, the rationale behind key experimental choices is not clearly described. For example, it is often unclear why specific strains or clade representatives were selected for experiments. Why was only JSG6133 selected for most of experiments? Were the results similar with other clade representatives?
a. For example, how was the region of the CdMecA selected for the truncated recombinant form (line 114-115)? Providing the explanation will help readers to understand the experimental design and findings.
8) The impact is primarily assessed through phenotypic readouts, such as vegetative cells, spores or measuring PBPs in the membrane fraction of lysates. However, no supplementary studies were performed to examine expression at transcriptional level or broader protein level. This assessment would provide important mechanistic insights to help support whether the observed phenotype results from changes in expression and/or activity.
9) While it could be said that CdSpoVD is required for the activity of CdMecA, the experiments are not conclusive enough to support the model in figure 1g.
10) It is not clear that the MecA acts mechansically like PBP2a.

Minor comments

11) Figure resolution issues compromise data interpretation e.g., Figure 4 and extended figure 1c
12) Table S1 needs more information on the two isolates.
13) C. perfringens MICs should also be done for the complemented strain.
14) Ceftaroline another inhibitor of PBP2a could be included as a control in the study
15) The effect of CdMecA on MICs to other beta-lactams in C. difficile (e.g., oxacillin or ampicillin) and surrogate organisms should be executed to confirm the MecA homology is functionally similar to MRSA PBP2a as might be suggested by the paper.
16) Line 114-118: the target engagement assay should be done also using ceftobiprole to indicate it is amenable to inhibition
17) Throughout the study should specify cefotetan is being used specifically as the cephamycin as opposed to broadly state cefomycin.
18) Experiments stated in line 200-208 should be re-written to improve clarity.
19) Animal experiments are needed to validate the clinical relevance of the mechanism.

Reviewer #2

(Remarks to the Author)
This manuscript by Srikhanta et al describes an alternate penicillin-binding protein that confers antibiotic resistance during sporulation of C. difficile and potentially other spore-forming pathogens. This is of significant import as antibiotics are the first line treatment for C. dif and the persistence of spores is a primary reason for frequent relapses in patients. The use of an additional antibiotic that blocks sporulation offered a possibly effective improvement in treatment, but this resistance mechanism could thwart this approach.
This resistance mechanism is a clearly orthologous genetic locus to that found in resistant S. aureus strains. The authors show that it is present in many strains of C. diff as well as in some strains of numerous other spore-formers.
The work has been completed with excellent methods and proper controls. The conclusions are therefore well supported in almost all cases. The work examines the system completely using genetic, physiological, and biochemical approaches. This work will be well cited and forms a basis for much future work and potentially for direct clinical application.
One observation is that the essentiality of SpoVD and/or MecA for cortex synthesis is highly suggestive of the cortex PG requiring 4-3 crosslinks that are produced by PBPs, as opposed to 3-3 cross-links produced by Ldts. Another publication has suggested that C. difficile spore cortex PG contains uniquely 3-3 crosslinks. These authors could note that their data suggest the contrary.(https://doi.org/10.1016/j.isci.2025.112227)
I have a few concerns with the data analysis and the presentation of conclusions.
1. Line 41: Should this say, “C. difficile strains” rather than “C. difficile species”?
2. Line 42: "hijack" is an odd term to use here. This word generally means to take over by force and often with negative intent. This would seem to be a positive event that doesn’t fully take over unless it is absolutely required.
3. Line 44: The results in this paper are exciting and important, but it is not clear how this "reshapes our understanding of antibiotic resistance." It seems to be a direct repetition of a well-studied antibiotic resistance mechanism, simply placed into the new context of the sporulation process. "reshapes our understanding of antibiotic resistance" could be restated.
4. Figure 2, panel A: I am not convinced that the right-hand part of this panel representing the amino acid alignment provides any useful information or is interpretable by the reader. It takes a lot of space here. A full alignment showing amino acids in Supplementary material could be more informative for those desiring that depth of inquiry.
5. Figure 3: The 2-fold difference in DPA between the strains (Panel D) is easily believable. This is hard to reconcile with the Raman difference (Panel E). If 80% of WT spores have NO DPA, as suggested by the Raman profile, then the difference in panel D should be greater. I suspect that:
A) Other differences between the WT and CdMecA+ spores result in an alteration of the Raman spectrogram, masking the detection of DPA in the WT spores;
B) The spores used in panel D and panel E are different, with those in Panel E having somehow lost their dehydration and DPA; OR
C) The Raman limit of detection is poor, resulting in lack off detection in many spores (least likely.)
How can the authors explain the difference between the Panel D and E results? This should be addressed in the manuscript around lines 177-182.
6. Line 667: Soluble muropeptides cannot be recovered by centrifugation. The insoluble spore debris can be removed by

centrifugation, leaving the muropeptides in the supernatant.
7. In Data Set 2 about peptidoglycan structure, in "Groups and Summary", it is not at all clear what is meant by "Glycan Chains" versus "Monomers". The muropeptides in each group seem to include short sugar backbone fragments, frequently with non-crosslinked peptide side chains. How are the authors differentiating these two groups?

Reviewer #3

(Remarks to the Author)
I co-reviewed this manuscript with one of the reviewers who provided the listed reports. This is part of the Nature Communications initiative to facilitate training in peer review and to provide appropriate recognition for Early Career Researchers who co-review manuscripts.

Reviewer #4

(Remarks to the Author)
This manuscript entitled "Uncovering an alternate pathway of antibiotic resistance in spore-forming bacteria" presents a compelling and well-supported discovery of an alternative pathway of antibiotic resistance in Clostridioides difficile, mediated by the acquisition and activation of a MecA-homologue (CdMecA) that functionally replaces the sporulation-specific PBP SpoVD. The authors convincingly show that CdMecA enables sporulation under cephamycin pressure, produces phenotypically distinct spores with increased resilience, and relies on SpoVE/SpoVD interactions to function. They further extend this mechanism to Clostridium perfringens and propose a dual-antibiotic strategy to overcome resistance.
This is highly original work of broad interest. Several points, however, require clarification, improved justification, or additional data presentation.

Major Comments
- The manuscript states that cefotetan does not efficiently bind CdMecA based on competition fluorescence-polarization assays using CdMecAΔ1–34. While this is an important result supporting the central model, the corresponding experimental data are, unless I missed them, not shown anywhere in the manuscript. This is a major issue, as the strength of the conclusion ("cefotetan failed to displace Boc-FL from CdMecA even at 4000 µM") cannot be evaluated without the presentation of these results. In addition, according to the Methods section, an essential control was performed: the ability of cefotetan to bind SpoVD, a known cephamycin-sensitive PBP. However, this positive control is not reported in the Results section. Demonstrating that cefotetan can displace Boc-FL from SpoVD is crucial to confirm that the assay is functioning properly and that the lack of displacement observed for CdMecA is meaningful.
-The analysis of MecA prevalence across spore-formers is not fully convincing. The reported candidates show only 26–35.6% nucleotide identity and 30.5–48.6% amino-acid identity to CdMecA, values that might be considered as too low to confidently infer homology or functional equivalence. To further strengthen this analysis, the authors could perform comparative AlphaFold2 structural predictions as in Extended data figure 2B. Structural conservation of the MecA domain and transpeptidase fold would support the classification of these proteins as true MecA homologues.
- In addition, the manuscript does not provide any functional validation showing that these distant MecA orthologues behave as cdMecA and enable sporulation in the presence of cefotetan. To strengthen their conclusions, the authors should consider possible functional validation strategies. For example, comparing sporulation levels in the presence or absence of cefotetan in a Clostridium perfringens or Bacillus subtilis strain carrying a putative MecA homologue versus a closely related non-MecA strain. Alternatively, the authors could express a mecA candidate in a C. difficile strain lacking mecA or in the C. difficile mecA knockout strain to determine whether it restores cefotetan-associated sporulation resistance.
- The regulatory aspects of the mec locus require further explanation. First, unlike S. aureus MecI, which represses mecA, disruption of CdmecI does not increase MecA production in the absence of antibiotics (Extended Data Fig. 2f). This unexpected result should be discussed. Does CdMecI not function as a repressor? Could other regulatory mechanisms mask its effect? Second, the authors report that CdmecI and CdmecR are frequently present in strains lacking CdmecA. The biological significance of this is unclear. Are these regulatory genes remnants of a formerly complete mec locus? Do they regulate another gene in these strains? It would greatly strengthen the manuscript to examine in more detail the genomic context of mecI/mecR in several mecA-negative strains to determine whether another gene or operon could plausibly be under the control of this system.
- In Fig. 5, dual treatment with cefotetan and ceftobiprole "abolished sporulation," whereas cefotetan alone in the mecA mutant only strongly reduces, but does not eliminate, spore production. The manuscript would benefit from clarification of why a complete suppression of sporulation is observed in one case but only partial suppression in the other, e.g., differences in antibiotic concentration, timing, culture density, or quantification methods.

Minor comments
- The authors report that sporulation in strains belonging to Clades 4, 5, and C-III is not inhibited by cefotetan, and later note that CdmecA is absent from all C-III isolates. This observation implies that CdmecA alone cannot account for the cefotetan-insensitive sporulation phenotype across all lineages. While it is entirely plausible that additional, lineage-specific mechanisms exist, this should be explicitly acknowledged in the text to avoid over-attributing this phenotype to MecA.
- At line 111, the authors conclude that CdMecA's role is "specific to sporulation, not vegetative cell survival." This is misleading, given that the manuscript later notes (line 271) that CdMecA also confers β-lactam resistance in vegetative cells (as shown elsewhere). I recommend rephrasing this conclusion more precisely to indicate that the sporulation-specific role is observed in the context of cephamycin exposure, and that CdMecA has broader functions under different β-lactam conditions.

- Line 119 states that CdmecA is “part of an operon” with mecR and mecI. However, mecA is oriented divergently relative to mecR/mecI, which is inconsistent with a single operon. This sentence should be revised to accurately reflect the transcriptional organization.
- Including the mecA mutant treated with cefotetan as a control in the spore-resilience experiments in Fig. 3 strengthens the interpretation that increased resistance is associated with MecA-dependent sporulation rather than with the presence of the antibiotic. However, Fig. 1 shows that this mutant produces extremely few spores under cephamycin exposure (~4×10^2 spores/mL). It is therefore unclear how sufficient material was obtained for purification and downstream assays. The authors should clarify whether spores used in Fig. 3 were harvested under identical conditions as those quantified in Fig. 1.
- Extended Data Fig. 4 uses several acronyms that may be unfamiliar to non-specialists. These should be defined directly in the figure legend.
- Supplementary Tables 7 and 8 are not referenced in the main text. All supplementary materials should be explicitly cited at least once to clarify their relevance and guide readers.

Version 1:

Reviewer comments:

Reviewer #1

(Remarks to the Author)
In the reviewer's opinion, the authors have conscientiously responded to critiques raised. I would recommend the authors consider adding to the discussion, only relevant information that can be obtained from their response to critique 1 "Taken together, these findings suggest that although CdMecA is not currently associated with increased disease burden, its presence in emerging and environmentally prevalent clades supports the need for continued genomic surveillance. Monitoring these lineages is important to detect early shifts in pathogenicity or resistance that may impact clinical and public health outcomes."

Reviewer #2

(Remarks to the Author)
The authors have adequately addressed my concerns from the initial review.

Reviewer #4

(Remarks to the Author)
The authors have thoroughly and satisfactorily addressed all of my previous concerns. The revised manuscript is substantially strengthened, and the additional data, clarifications, and textual revisions fully resolve the points I raised.

I have no further comments. I am satisfied with this new version of the manuscript and with the work carried out by the authors.

I support publication of the manuscript in its revised form.

Open Access This Peer Review File is licensed under a Creative Commons Attribution 4.0 International License, which permits use, sharing, adaptation, distribution and reproduction in any medium or format, as long as you give appropriate credit to the original author(s) and the source, provide a link to the Creative Commons license, and indicate if changes were made.
In cases where reviewers are anonymous, credit should be given to 'Anonymous Referee' and the source.
The images or other third party material in this Peer Review File are included in the article's Creative Commons license, unless indicated otherwise in a credit line to the material. If material is not included in the article's Creative Commons license and your intended use is not permitted by statutory regulation or exceeds the permitted use, you will need to obtain permission directly from the copyright holder.
To view a copy of this license, visit https://creativecommons.org/licenses/by/4.0/

Point-by-point response to reviewer comments

NCOMMS-25-88923-T: **Uncovering an alternate pathway of antibiotic resistance in spore-forming bacteria**

We sincerely thank the reviewers for their thoughtful and constructive evaluation of our work. We have addressed all comments in detail below and revised the manuscript accordingly. The suggested changes have materially strengthened the clarity, interpretation, and overall quality of the manuscript, and we appreciate the opportunity to revise it in response to the feedback.

Note that line numbers refer to the document showing track changes. Reviewer comments are reproduced in full in black text and our responses are in blue text.

REVIEWER COMMENTS AND RESPONSES:

Reviewer #1 (Remarks to the Author):

The manuscript by Srikhanta et al. describes the discovery of an MRSA mecA-related gene in C. difficile, which the authors suggest plays a antibiotic resistance role in C. difficile and possibly other spore-forming bacteria. The manuscript builds on the communicating author's previous work that cephamycin can inhibit sporulation in C. difficile by targeting the conserved spore-specific protein SpoVD, penicillin-binding protein. The author's discovery of mecA-related gene was guided by the observation that cephamycins lacked anti-sporulation activity against certain C. difficile. Here, the authors investigated the genetic and mechanistic basis underlying the clade-specific difference in the anti-sporulation effect of cephamycins. They identified CdMecA in Clade 4-5 and C-III that can functionally compensate for SpoVD inhibition by cephamycins to restore sporulation in the presence of cephamycin. The findings explained the lack of cephamycin anti-sporulation effect across isolates from different clades. The authors showed that ceftobiprole combined with cefotetan retained ability to inhibit sporulation, in otherwise cephamycin-resistant strains. While the authors suggest this has therapeutic potential, it seems unlikely that two beta-lactams will be given to control CDI to exacerbate dysbiosis, including disrupting firmicute population. No animal studies support this assertion by the authors. Overall, while the study provides interesting insights into a new clade-specific resistance pathway associated with sporulation in C. difficile, several aspects of the experimental rigor and data interpretation raise concerns. Major comments are below:

1) The study identified CdMecA in Clade 4-5 and C-III, this provides valuable insight into the genomic diversity of C. difficile. Clade 5 (ribotype 078) is associated with disease, and Clade 4 and cryptic clade C-III are rarely associated with disease. The current clinical significance remains unclear.

We agree that the current clinical significance of *Cd*MecA in Clade 4, Clade 5, and cryptic clade C-III remains incompletely defined. However, *C. difficile* epidemiology is highly dynamic and influenced by antibiotic selective pressures across One Health environments. The emergence of MecA is therefore likely linked to antimicrobial exposure, and its acquisition and persistence may contribute to resistance phenotypes that could become clinically relevant over time.

Importantly, disease association is evolving across clades traditionally considered of low clinical significance. For example, Clade 4 strains (ST37/RT017) have been increasingly reported in clinical settings (PMID: 29553322; PMID: 29467194), and notably represented 35.8% of *Cd*MecA-positive isolates in our survey (see response to Question 5). This demonstrates that clades historically regarded as infrequent causes of disease can emerge as clinically important under changing selective pressures.

While strains from cryptic clade C-III have not yet been directly linked to human disease, their strong representation in environmental reservoirs (PMID: 35166655) suggests a potential role in community-associated transmission and future emergence. Environmental prevalence provides opportunities for genetic exchange and adaptation that may precede clinical relevance.

Taken together, these findings suggest that although *Cd*MecA is not currently associated with increased disease burden, its presence in emerging and environmentally prevalent clades supports the need for continued genomic surveillance. Monitoring these lineages is important to detect early shifts in pathogenicity or resistance that may impact clinical and public health outcomes.

2) Figure 1d: It appears that introducing CdMecA into M7404 cells may not fully prevent the action of cefotetan. The impact in terms of log reduction is similar to that observed in figure 1a for M7404 exposed to cefotetan alone.

We appreciate the reviewer's observation and agree that, when visually comparing Figures 1a and 1d, the overall log reduction following cefotetan exposure appears similar. However, this comparison spans across experiments performed at different times, which introduces inherent biological and technical variability. In *C.*

difficile, sporulation efficiency is notoriously difficult to synchronise, and additional variability may arise from differences in cefotetan batches, uptake efficiency, and experimental timing.

Importantly, the MecA+ strain was generated by introducing the construct *in trans* into M7404, and therefore complete inhibition of cefotetan-mediated effects is not expected. Instead, only partial protection against sporulation inhibition is anticipated under these conditions.

When considering Figure 1d in isolation, where wild-type and MecA+ M7404 strains were assayed contemporaneously using the same cefotetan batch, a clear and statistically significant protective effect of *Cd*MecA is observed. Specifically, cefotetan-treated M7404 MecA+ cultures produced approximately 1,000-fold more spores compared to cefotetan-treated wild-type M7404, indicating that *Cd*MecA mitigates, but does not fully abrogate, cefotetan-induced sporulation inhibition.

To address this point explicitly, we have revised the manuscript to state that *Cd*MecA confers *partial protection* against cefotetan-mediated inhibition of sporulation (line 110). In addition, we have strengthened the robustness of this result by including additional experimental replicates in Figure 1d, which further support the observed protective effect.

a. In this experiment, and maybe strain background, it appears CdMecA is not the only factor driving the phenotype.

We agree that *Cd*MecA is unlikely to be the sole determinant of the observed phenotype. Additional factors, including introduction of the construct *in trans* rather than chromosomal integration, strain-specific background effects, and experimental variability in cefotetan activity, are likely to contribute to the outcome.

Nevertheless, a consistent and measurable contribution of *Cd*MecA is evident in Figure 1d. Specifically, following cefotetan treatment, the M7404 MecA$^+$ strain produces approximately a 10^3-fold higher spore count compared to the corresponding wild-type control. This indicates that while *Cd*MecA does not fully account for the phenotype, it confers a reproducible and statistically significant protective effect under these experimental conditions.

Overall, these data support a model in which *Cd*MecA acts as a contributing, rather than exclusive, factor in modulating cefotetan-mediated inhibition of sporulation.

b. It would be helpful if authors could discuss this possibility of the above, and provide additional data to clarify the extent to which CdMecA functionally compensates in the M7404 background.

We thank the reviewer for highlighting the importance of discussing additional contributing factors and for suggesting PBP profiling as a means to clarify the extent of functional compensation by *Cd*MecA in the M7404 background. We agree that this analysis provides valuable mechanistic insight.

To address this point, we have now examined PBP profiles in wild-type M7404 and M7404 MecA$^+$ strains under both untreated and cefotetan-treated conditions (new Extended Data Fig. 1). Under untreated conditions, the PBP profiles of wild-type and MecA$^+$ strains were highly similar, indicating that introduction of *Cd*MecA does not broadly alter PBP composition in the absence of antibiotic pressure.

Following cefotetan exposure, both strains exhibited comparable overall PBP patterns, including a pronounced reduction in SpoVD signal, consistent with cefotetan-mediated inhibition of SpoVD. Notably, an additional PBP band corresponding to *Cd*MecA was detected exclusively in the MecA$^+$ strain under cefotetan treatment. This confirms that *Cd*MecA is expressed and stable in the M7404 background during antibiotic exposure.

Together, these data demonstrate that while *Cd*MecA does not restore sporulation to SpoVD levels, its presence coincident with SpoVD inhibition supports a model of functional compensation rather than complete replacement. This is consistent with the partial rescue of sporulation observed in Figure 1d and indicates that *Cd*MecA contributes measurably to maintaining sporulation capacity under cefotetan stress. We have incorporated this discussion (lines 325-326) into the revised manuscript and added the PBP profiling data as Extended Data Figure 1e in the Supplementary Materials.

c. Knowing the PBP profiles in M7404 would also be useful.

Please see response above.

3) While TargeTron-based was used to inactivate CdmecR and CdmecI, two key control experiments are missing (see below, a, b); in the absence of these experiments, it is difficult to conclude with confidence that CdmecA is under regulatory control of MecR.

We thank the reviewer for this important comment and agree that appropriate controls are essential to assess whether *Cd*MecA is under regulatory control of the MecR-MecI system. To address these concerns, we

performed both complementation experiments and expression analyses to rule out polar effects, as detailed below.

a. No complementation studies were not performed.

We agree that complementation is necessary to support conclusions drawn from insertional inactivation. Accordingly, we performed *in trans* complementation of both the *mecI* and *mecR* mutants and assessed sporulation under untreated and cefotetan-treated conditions (Extended Data Fig. 2f).

For the *mecI* mutant, sporulation assays revealed no statistically significant differences among the wild-type strain, the *mecI* mutant, and the complemented *mecI* strain under either condition. This is consistent with *mecI* acting as a repressor whose inactivation does not impair sporulation capacity and supports its regulatory, rather than structural role.

In contrast, complementation of the *mecR* mutant resulted in a clear partial restoration of sporulation. Specifically, the complemented *mecR* strain exhibited an approximately 10^3-fold increase in spore formation compared to the *mecR* mutant, which failed to sporulate under cefotetan treatment (Extended Data Fig. 2f). These data demonstrate that the sporulation defect observed in the *mecR* mutant is attributable to loss of MecR function and is at least partially reversible.

b. No expression studies(qPCR) to assess polar effects

To determine whether the observed phenotypes could be explained by transcriptional polarity arising from TargeTron insertion, we performed digital PCR analysis across the *mec* gene complex (Extended Data Fig. 2g). These analyses revealed no evidence of polar effects in either mutant background.

Disruption of *mecR* did not reduce *mecI* transcript levels, and insertional inactivation of *mecI* did not diminish *mecR* transcription under either untreated or antibiotic-stressed conditions. Importantly, *mecA* transcription was not reduced in either mutant; instead, *mecA* expression was elevated in the *mecI* mutant, consistent with derepression upon loss of MecI.

Together, these expression data support regulatory control of *mecA* by the MecR-MecI system rather than indirect effects caused by transcriptional polarity. When combined with the complementation results, they provide strong evidence that *Cd*MecA expression and function are modulated by MecR-MecI-dependent regulation.

4) In several experiments, key control data appears to be missing, this makes it difficult to fully interpret the results. Also, statistical comparison between key experimental conditions is often missing. For example, figure 1c is missing the complement untreated group. Figure 1d, no statical analysis between +MecA untreated and treated group was done. Other figures include Figure 4b (SpoVD complement untreated) extended figure 1b and 1c (Untreated complement), extended figure 6c (spoVD complement UT), extended figure 1d (complement strains).

We thank the reviewer for this careful assessment and apologise for the omissions and incomplete statistical analyses in the original submission. We agree that inclusion of appropriate control groups and rigorous statistical comparisons are essential for accurate interpretation of the data. In response to this comment, we have revised the manuscript to include the missing controls and completed the relevant statistical analyses, as outlined below.

Added control data:

- Figure 1c: Spore counts for the untreated *mecA* mutant complemented strain have been added.
- Extended Data Figure 1c (formerly Extended Data Figure 1b): Vegetative cell counts for the untreated *mecA* complement strain are now included.
- Figure 4b: Spore counts for the untreated *spoVD* mutant complemented strain have been added.
- Extended Data Figure 9e (formerly Extended Data Figure 6c): Vegetative cell counts for the untreated *spoVD* mutant complemented strain have now been included.

Statistical analyses:

- Figure 1d: Statistical comparisons have now been performed across all relevant groups, including untreated versus cefotetan-treated M7404 MecA$^+$ strains. These analyses confirm a statistically significant, partial protective effect of *Cd*MecA under cefotetan exposure.

Clarification of specific figures and experimental intent:

- Extended Data Figure 1b (formerly Extended Data Figure 1c; PBP profiling): This experiment was designed specifically to confirm the absence (i.e. lack of induction) of *Cd*MecA in the *CdmecA* mutant

background, thereby validating the genetic context of the sporulation assays. It was not intended to quantitatively mirror the sporulation phenotypes, and we have clarified this purpose in the revised Extended Data Figure 1 legend.

- Figure 1d (M7404 *mecA* sporulation assay): Complemented strains were not included in this experiment as the assay was designed to assess the effect of introducing *mecA* into a strain that does not naturally encode it. In this context, *mecA* was introduced *in trans* into the M7404 background, and comparison to a complemented deletion strain was therefore not applicable. This rationale has now been clarified in the manuscript line 108).

Together, these revisions address the reviewer's concerns regarding missing controls and statistical comparisons, and they substantially strengthen the robustness and interpretability of the experimental conclusions. We are grateful to the reviewer for highlighting these points, which have improved the overall quality and clarity of the manuscript.

5) Line 127 to 134: the analysis is largely descriptive and lacks deeper interpretation. It would strengthen the manuscript if the authors could discuss the evolutionary or functional implications of this distribution. Also, it is intriguing that CdmecA is highly prevalent in more environmentally relevant clades but not clinically common clades. Could the authors comment on this?

We thank the reviewer for this thoughtful and constructive comment and agree that deeper interpretation of the distribution of *CdmecA* strengthens the manuscript. In response, we have expanded both the Results and Discussion to address the evolutionary and functional implications of the observed clade- and lineage-specific prevalence.

Although *CdmecA* might be intuitively expected to associate primarily with environmentally adapted clades, our analysis instead shows that the complete *mecAIR* locus is most prevalent in several well-established human epidemic *C. difficile* lineages. Excluding rare sequence types, the highest prevalences were observed in STs 3 (46.9%), 4 (57.9%), 11 (56.2%), 37 (35.8%), and 81 (100%), corresponding to ribotypes 001, 014/020, 078, 017, and 369, respectively. Notably, ribotypes 014/020, 017, and 078 are well recognised for their capacity to harbour diverse accessory antimicrobial resistance determinants, largely acquired *via* horizontal gene transfer from other members of the gut microbiota in antibiotic-rich environments (PMID: 28123380; co-author Dan Knight, 35316173; co-author Korakrit Imwattana, 30862754). We therefore suggest that the acquisition and maintenance of CdmecA likely occurred through similar processes, but its contribution has remained largely unrecognised due to *C. difficile*'s intrinsic cephalosporin resistance mediated by alternative mechanisms.

This pattern contrasts with ST1 (ribotype 027), a globally successful epidemic lineage in which *CdmecA* prevalence is extremely low (0.2%). Unlike the lineages noted above, ST1 primarily relies on point mutations in DNA gyrase subunits to confer fluoroquinolone resistance and carries relatively few accessory resistance genes (PMID: 34793295; co-author Korakrit Imwattana). Furthermore, the emergence of ST1 is comparatively recent (early 2000s), affording less evolutionary time for the accumulation of additional resistance loci. We also note that the rise of ST1 occurred during a period when clindamycin use had declined due to concerns over *C. difficile* infection, potentially reducing selective pressure for acquisition or retention of auxiliary resistance determinants such as *CdmecA*.

Importantly, although *CdmecA* is less frequent in historically dominant clinical clades such as ST1, its enrichment in lineages known for genetic plasticity and accessory gene acquisition suggests it may represent a latent adaptive trait. This is particularly relevant given the prevalence of *CdmecA* in clades with strong environmental and zoonotic links, where sustained antibiotic exposure may promote both retention and onward dissemination into clinically relevant populations.

To reflect these interpretations, we have added the following to the Results section (lines 152-153):

"Within these clades, the prevalence was highest in STs 3 (46.9%), 4 (57.9%), 11 (56.2%), 37 (35.8%), and 81 (100.0%), all of which are known epidemic lineages in humans."

We have also revised the Discussion (lines 347-350) to emphasise the functional and evolutionary context of *CdmecA* acquisition:

"Notably, *Cd*MecA was found in ~10% of surveyed *C. difficile* genomes, predominantly in Clades 4 and 5, especially STs 11, 37, and 81, all known to harbour other accessory resistance genes acquired from colonic bacteria. These lineages are likely to have acquired CdMecA through similar mechanisms."

Together, these additions provide a clearer evolutionary framework for interpreting the uneven distribution of *CdmecA* and highlight its potential relevance to future shifts in antimicrobial resistance and lineage emergence.

6) There is concern regarding clinical utility of using a combination of cefotetan and ceftobiprole in terms of damage to the microbiota. Animal efficacy and microbiome experiments will be needed to support the claim for therapeutic utility by the authors.

We appreciate the reviewer's concern regarding the potential impact of combining cefotetan and ceftobiprole on the host microbiota and agree that comprehensive animal efficacy and microbiome studies would be required to support any claims of therapeutic utility.

We wish to clarify that the cefotetan-ceftobiprole combination experiments were performed strictly as proof-of-principle to demonstrate that simultaneous, targeted inhibition of *Cd*SpoVD and *Cd*MecA is mechanistically feasible in *C. difficile* strains carrying *CdmecA*. This intent is explicitly stated in the manuscript (lines 379-381):

"These findings provide proof-of-principle that targeted inhibition of PBP activity could form the basis for rational anti-sporulation strategies, with ceftobiprole offering a viable path forward."

We do not propose this antibiotic combination as a clinically deployable therapy in its current form. We fully agree that any translational advancement would require animal efficacy studies, alongside microbiome analyses, ideally using derivatives or alternative compounds with improved specificity for *C. difficile* to minimise collateral microbiota damage. Such investigations are beyond the scope of the present study and would necessarily precede any consideration of therapeutic application.

Accordingly, we have been careful not to make claims regarding clinical utility in the absence of *in vivo* data. Our conclusions are intentionally framed around mechanistic insight and conceptual feasibility rather than immediate therapeutic implementation, and we believe this approach is consistent with both the scope of the study and the principles of the 3Rs (replacement, reduction, and refinement) guiding responsible use of animals in research.

7) At several places, the rationale behind key experimental choices is not clearly described.

a. For example, it is often unclear why specific strains or clade representatives were selected for experiments. Why was only JSG6133 selected for most of experiments? Were the results similar with other clade representatives?

We thank the reviewer for highlighting the need to more clearly articulate the rationale behind our experimental strain selection. We agree that this context is important for appropriate interpretation of the results, and we have clarified this point in the revised manuscript.

C. difficile strains vary substantially in their amenability to genetic manipulation. To ensure that our observations were not strain- or clade-specific, we initially performed sporulation assays and PBP profiling on representative strains from Clades 4, 5, and cryptic clade C-III. These experiments consistently showed comparable *Cd*MecA-mediated attenuation of the anti-sporulation effect of cefotetan across all three clades, indicating that the underlying phenotype was conserved.

Based on these similar outcomes, we selected the Clade 5 strain JGS6133 as the primary experimental representative for subsequent mechanistic studies. This strain was chosen because it is significantly more genetically tractable than other clade representatives, enabling efficient mutant construction and reliable *in trans* plasmid complementation. Focusing on a single, tractable background allowed us to perform the necessary genetic, transcriptional, and biochemical analyses with sufficient depth and reproducibility.

In addition, *C. difficile* M7404, which lacks *CdmecA*, was included as a comparator strain. This strain provides an informative negative control, as we have previously demonstrated that cephamycins markedly reduce sporulation in this genetic background, allowing clear attribution of phenotypic rescue to the presence of *Cd*MecA.

To address the reviewer's concern directly, we emphasise that JGS6133 was not selected because it exhibited a unique phenotype, but rather because it recapitulated the responses observed across multiple clades while offering substantial practical advantages for downstream experimentation. This rationale is now explicitly stated in the revised manuscript (lines 104-105).

b. For example, how was the region of the CdMecA selected for the truncated recombinant form (line 114-115)? Providing the explanation will help readers to understand the experimental design and findings.

We thank the reviewer for raising this point and agree that explicitly explaining the rationale for the truncated *Cd*MecA construct strengthens the clarity of the experimental design.

The recombinant *Cd*MecA construct was designed to remove the predicted N-terminal transmembrane helix, generating a soluble form of the protein suitable for biochemical analysis. Full-length *Cd*MecA is membrane associated, which complicates recombinant expression, purification, and downstream biophysical assays. Truncation of the transmembrane region is therefore a standard and necessary strategy to obtain a stable,

soluble protein while preserving the catalytically and functionally relevant domains required for ligand-binding studies.

Accordingly, we expressed a *Cd*MecA variant lacking residues 1-34 (*Cd*MecA$_{\Delta 1\text{-}34}$), corresponding to the predicted transmembrane helix, and used this recombinant protein in fluorescence polarization assays to directly assess PBP–ligand interactions. This design allowed us to interrogate the biochemical properties of *Cd*MecA in a controlled, interpretable manner without altering its predicted functional core.

We have now clarified this rationale in the main text (lines 123-124), where the following statement has been added:

"To test this, we produced a truncated recombinant form of *Cd*MecA (*Cd*MecA$_{\Delta 1\text{-}34}$), removing a transmembrane helix to produce a soluble form of the protein, and performed fluorescence polarization assays."

8) The impact is primarily assessed through phenotypic readouts, such as vegetative cells, spores or measuring PBPs in the membrane fraction of lysates. However, no supplementary studies were performed to examine expression at transcriptional level or broader protein level. This assessment would provide important mechanistic insights to help support whether the observed phenotype results from changes in expression and/or activity.

We thank the reviewer for this constructive comment and agree that integrating transcriptional analysis with phenotypic readouts provides important mechanistic insight.

In response, we have expanded the study to include direct assessment of gene expression at the transcriptional level.

Specifically, we performed digital PCR on RNA isolated during sporulation from wild-type, *CdmecI*, and *CdmecR* mutant strains under both untreated and cefotetan-treated conditions. Transcripts for *CdmecA*, *CdmecI*, and *CdmecR* were quantified (Extended Data Fig. 2g). These data demonstrate that *CdmecA* expression is inducible in response to cefotetan and is differentially regulated depending on the regulatory background.

Disruption of *CdmecI* resulted in elevated *CdmecA* transcription under both untreated and antibiotic-exposed conditions, consistent with relief of transcriptional repression (Extended Data Fig. 2g). In contrast, loss of *CdmecR* leads to dysregulation of cefotetan-induced *CdmecA* expression, indicating post-transcriptional limitation (Extended Data Fig. 2g and 2e). The Results section has been updated accordingly to reflect these findings (lines 137-141).

Importantly, these transcriptional data align with our functional assays. Sporulation assays and PBP profiling of membrane fractions show that disruption of *CdmecR* abolishes cefotetan-induced *Cd*MecA production and sporulation rescue, whereas disruption of *CdmecI* does not impair these phenotypes.

While we acknowledge that global proteomic analyses could provide additional resolution, we believe that the combined transcriptional data, PBP profiling, and phenotypic assays establish a clear mechanistic link between MecR-MecI regulation, *CdmecA* expression, and the resulting sporulation phenotype. These additions substantially strengthen the causal interpretation of the experimental findings.

9) While it could be said that CdSpoVD is required for the activity of CdMecA, the experiments are not conclusive enough to support the model in figure 1g.

We thank the reviewer for this important and constructive comment and agree that the current data do not yet provide definitive proof for the mechanistic model proposed in Figure 1g. We fully acknowledge that additional biochemical experiments, such as *in vitro* peptidoglycan synthesis assays (e.g. PMID: 28553948), would be required to conclusively validate this model. These experiments are beyond the scope of the present study. To reflect this limitation explicitly, we have revised the manuscript to temper our conclusions and now state:

"However, further experimental validation is required to substantiate this model." (line 305)

That said, we believe our data provide supportive evidence for a functional interdependence between *Cd*SpoVD and *Cd*MecA during spore peptidoglycan synthesis, which motivated the proposed model. Specifically, our conclusions are based on the following observations:

1. Functional dependence during sporulation: Sporulation proceeds in the presence of cefotetan when *Cd*SpoVD is catalytically inactive yet present (Fig. 4), indicating that *Cd*MecA activity alone is insufficient in the absence of *Cd*SpoVD and suggesting a requirement for *Cd*SpoVD in this process.
2. Physical interaction: *Cd*MecA and *Cd*SpoVD were found to associate with one another, as demonstrated by co-immunoprecipitation, blue native PAGE, and bacterial two-hybrid analyses (Fig. 4), supporting the notion of a functional complex rather than independent activity.

To further clarify the tentative nature of this model, we have revised Figure 4g accordingly: “cephamycins” has been replaced with “cefotetan” for specificity, and a question mark has been added to *Cd*SpoVE to explicitly indicate that its proposed transglycosylase role remains hypothetical.

Taken together, while we agree that the data do not yet conclusively establish the mechanistic model depicted in Figure 1g (Fig. 4g), they are consistent with a requirement for *Cd*SpoVD in *Cd*MecA-mediated spore peptidoglycan synthesis. We have therefore reframed the model as a working hypothesis supported by genetic, functional, and interaction data, and we clearly delineate its current limitations to avoid over-interpretation.

10) It is not clear that the MecA acts mechansically like PBP2a.

We appreciate the reviewer’s comment and agree that it is important to clearly distinguish the extent to which *Cd*MecA is mechanistically analogous to PBP2a.

*Cd*MecA and PBP2a do share key functional features: both are class B penicillin-binding proteins and function as transpeptidases that retain activity in the presence of specific β-lactam antibiotics. This ability to sustain transpeptidation under β-lactam pressure is their principal shared mechanistic characteristic and is what distinguishes them from most other class B PBPs.

However, we agree that *Cd*MecA should not be interpreted as operating identically to PBP2a. The two proteins differ substantially in their biological context, interaction partners, and resistance profiles. PBP2a in *Staphylococcus aureus* functionally replaces the transpeptidase activity of the bifunctional class A PBP2 during vegetative growth under methicillin exposure, relying on the glycosyltransferase activity of native PBP2 to support cell wall synthesis. In contrast, *Cd*MecA substitutes for the transpeptidase activity of SpoVD, a class B PBP specifically required for peptidoglycan synthesis during sporulation. Consistent with this distinct role, *Cd*MecA operates in the presence of cephamycins rather than anti-staphylococcal β-lactams and, as shown here (Fig. 4d–f), directly interacts with SpoVD, highlighting a mechanistic partnership different from that of PBP2a.

If the reviewer’s concern relates to the detailed molecular mechanism underlying β-lactam resistance, such as the well-characterised allosteric regulation described for PBP2a, we agree that this represents an important distinction. Elucidating whether *Cd*MecA employs similar or divergent molecular strategies will require dedicated structural and biochemical analyses, which are beyond the scope of the present study and will be the focus of future work.

To address this point explicitly and avoid over-interpretation, we have revised the manuscript to clearly articulate both the similarities and differences between CdMecA and PBP2a (lines **258-259**). The revised text now reads:

“PBP2a (MecA) from *S. aureus* and *Cd*MecA are class B PBPs and both function as transpeptidases in the presence of specific β-lactams. PBP2a in *S. aureus* requires the native class A bifunctional PBP2 glycosyltransferase to synthesise vegetative cell peptidoglycan under β-lactam pressure.”

We believe this clarification better reflects the conceptual relationship between *Cd*MecA and PBP2a while accurately delimiting the mechanistic conclusions supported by our data.

Minor comments

11) Figure resolution issues compromise data interpretation e.g., Figure 4 and extended figure 1c

We apologise for the low resolution of Figure 4 and Extended Data Figure 1c. This issue has now been rectified.

12) Table S1 needs more information on the two isolates.

Strain JGS6133 was previously described (Ref. 10) and was listed in Table S1; the toxin type for this strain has now been included. Strains MA0129 and A3491 are from our in-house collection (originally obtained from Prof. Tom Riley, University of Western Australia). Additional information, including ribotypes and toxin types, have now been added to Table S1 for both these strains.

13) C. perfringens MICs should also be done for the complemented strain.

MICs were performed to determine the sub-inhibitory concentration of cefotetan to use in the sporulation assays (Figure 2b) which was listed in Table S1 (0.078 ug/mL). However, we did not add the MIC value (0.156 ug/mL). This has now been added to Table S1.

14) Ceftaroline another inhibitor of PBP2a could be included as a control in the study

We thank the reviewer for this constructive suggestion and agree that inclusion of an additional PBP2a inhibitor strengthens the study. In response, we have now performed sporulation assays using ceftaroline,

another well-characterised inhibitor of PBP2a. Co-treatment with ceftaroline and cefotetan resulted in complete abolition of sporulation, mirroring the effect observed with ceftobiprole in combination with cefotetan. These data are now included in the Supplementary Materials (new Extended Data Fig. 11) and described in the revised manuscript (lines 312-319).

While both ceftaroline and ceftobiprole provide useful mechanistic validation, we elected to focus the main analysis on ceftobiprole for biological and translational reasons. Ceftaroline, similar to ceftriaxone, has been reported to induce simulated *C. difficile* infection in a human gut model (PMID: 23557928), indicating a more substantial impact on the gut microbiota. In contrast, ceftobiprole has been shown to have minimal disruption of gut microbial communities (PMID: 27659735), making it a more appropriate comparator for proof-of-principle studies aimed at rational anti-sporulation strategies.

We believe that inclusion of ceftaroline as a supplementary control further supports our conclusions while maintaining a clear focus on compounds with more favourable microbiota profiles.

15) The effect of CdMecA on MICs to other beta-lactams in C. difficile (e.g., oxacillin or ampicillin) and surrogate organisms should be executed to confirm the MecA homology is functionally similar to MRSA PBP2a as might be suggested by the paper.

We thank the reviewer for this thoughtful suggestion and agree that assessing the effect of *Cd*MecA on susceptibility to other β-lactams is important for clarifying its functional relationship to MRSA PBP2a.

To address this point, we examined the impact of *Cd*MecA on β-lactam susceptibility by comparing MICs for multiple β-lactams, including imipenem, oxacillin, and methicillin, between the JGS6133 wild-type strain and its *mecA* mutant. No differences in MICs were observed between these strains. These findings indicate that, in this genetic background, *Cd*MecA alone does not confer enhanced resistance to these β-lactams during vegetative growth.

As discussed in the manuscript (lines 344-345), prior studies have shown that carbapenem resistance in RT017 isolates requires additional mutations in *pbp1* and *pbp3* (PMID: 29553322). These mutations are absent in the JGS6133 background used here. This highlights that, although *Cd*MecA is homologous to MRSA PBP2a, its functional contributions to β-lactam resistance are likely context dependent and may require complementary genetic changes to manifest at the MIC level.

While testing *Cd*MecA function in surrogate organisms could further elucidate mechanistic similarities to MRSA PBP2a, such experiments fall outside the scope of the present study. We therefore note this as a limitation and emphasise that our conclusions focus on *Cd*MecA's role in sporulation-associated peptidoglycan synthesis rather than broad β-lactam resistance phenotypes.

We believe these additional data and clarifications help appropriately frame the functional distinctions between *Cd*MecA and MRSA PBP2a while avoiding over-interpretation.

16) Line 114-118: the target engagement assay should be done also using ceftobiprole to indicate it is amenable to inhibition

We thank the reviewer for this thoughtful suggestion and agree that assessing target engagement with ceftobiprole would further strengthen mechanistic understanding of *Cd*MecA inhibition.

While we did not perform fluorescence polarization-based target engagement assays specifically with ceftobiprole, our penicillin-binding protein (PBP) profiling experiments directly demonstrate that ceftobiprole binds *Cd*MecA. As shown in Figure 5b, both BocFL and ceftobiprole label *Cd*MecA, confirming that the protein is accessible to β-lactam binding and therefore amenable to inhibition by ceftobiprole under the experimental conditions used.

These binding data provide functional evidence of target engagement and complement the phenotypic and genetic observations presented in the study. We agree, however, that more detailed quantitative binding and inhibition assays, directly comparing ceftobiprole and other β-lactams, would provide additional mechanistic insight into *Cd*MecA-ligand interactions.

Such analyses form part of our ongoing work aimed at defining the molecular basis of MecA mediated β-lactam resistance and will be reported in a dedicated future study. We have therefore limited the current manuscript to establishing proof-of-principle target accessibility and functional relevance, without over-extending mechanistic claims beyond the data presented.

17) Throughout the study should specify cefotetan is being used specifically as the cephamycin as opposed to broadly state cefomycin.

Where the experiment was performed with cefotetan we have stated this and where it is a broader response we have used cephamycin. Cefotetan is used as the example cephamycin. We have changed cephamycin to cefotetan in line 113.

18) Experiments stated in line 200-208 should be re-written to improve clarity.

We thank the reviewer for highlighting the need for improved clarity in this section and agree that the original description would benefit from clearer framing and interpretation.

To address this, we have revised lines 200-208 (lines 228-237) to more explicitly describe the rationale, approach, and key findings of the experiment. The revised text now reads:

"To examine if the spore proteomes differed between *Cd*SpoVD and *Cd*MecA spores, quantitative proteomic analysis was performed. This analysis revealed significant differences in protein abundance between the two spore types (Supplementary Extended Data 5). *Cd*SpoVD spores exhibited increased abundance of proteins associated with β-lactam resistance (MecI; 4.22-fold), putative surface proteins (LytC_18; 2.35-fold and LytC_24; 3.15-fold), toxins (TcdA; 1.89-fold and TcdB; 1.76-fold), and a phosphotransferase system component (PTS sugar transporter subunit IIB; 2.49-fold). In contrast, *Cd*MecA spores showed increased abundance of the β-lactam resistance protein MecA (4.94-fold) and the carbohydrate transporter PbuX (2.15-fold). The reciprocal enrichment of *Cd*MecI in *Cd*SpoVD spores and *Cd*MecA in *Cd*MecA spores independently supports their respective roles in the cephamycin response and highlights distinct functional profiles between these spore populations."

We believe this revised wording more clearly conveys both the purpose of the experiment and the biological significance of the observed proteomic differences, improving readability and interpretability for the reader.

19) Animal experiments are needed to validate the clinical relevance of the mechanism.

We agree with the reviewer that *in vivo* models are ultimately important for validating the clinical relevance of the MecA mechanism. The primary objective of the present study, however, is to define the role and regulation of *Cd*MecA at the molecular and mechanistic levels. Accordingly, our conclusions are intentionally restricted to mechanistic insights derived from multiple, independent *in vitro* approaches, including genetic, biochemical, transcriptional, and phenotypic analyses.

We have been careful not to overstate the clinical or translational implications of these findings in the absence of animal data. Instead, we frame our results as establishing a mechanistic foundation upon which future *in vivo* studies can build. We acknowledge that the lack of animal model validation represents a limitation of the current work and explicitly note that such studies will be an important direction for future investigation. However, these experiments fall beyond the scope of this manuscript.

We believe that, within these defined boundaries, the data presented provide a robust and coherent mechanistic framework for understanding *Cd*MecA function and justify further investigation in relevant animal models.

Reviewer #2 (Remarks to the Author):

This manuscript by Srikhanta et al describes an alternate penicillin-binding protein that confers antibiotic resistance during sporulation of C. difficile and potentially other spore-forming pathogens. This is of significant import as antibiotics are the first line treatment for C. dif and the persistence of spores is a primary reason for frequent relapses in patients. The use of an additional antibiotic that blocks sporulation offered a possibly effective improvement in treatment, but this resistance mechanism could thwart this approach.

This resistance mechanism is a clearly orthologous genetic locus to that found in resistant S. aureus strains. The authors show that it is present in many strains of C. diff as well as in some strains of numerous other spore-formers.

The work has been completed with excellent methods and proper controls. The conclusions are therefore well supported in almost all cases. The work examines the system completely using genetic, physiological, and biochemical approaches. This work will be well cited and forms a basis for much future work and potentially for direct clinical application.

One observation is that the essentiality of SpoVD and/or MecA for cortex synthesis is highly suggestive of the cortex PG requiring 4-3 crosslinks that are produced by PBPs, as opposed to 3-3 cross-links produced by Ldts. Another publication has suggested that C. difficile spore cortex PG contains uniquely 3-3 crosslinks. These authors could note that their data suggest the contrary (https://doi.org/10.1016/j.isci.2025.112227)

We thank the reviewer for this constructive and supportive assessment and agree that the relationship between SpoVD/MecA activity and cortex peptidoglycan (PG) architecture is an important point to consider.

Regarding the nature of cortex PG crosslinks, we note that multiple studies, including an independent report (PMID: 30266804), have identified a predominance of 3-3 crosslinks in *C. difficile* spores, consistent with our findings. The absence of detectable 4-3 crosslinks in the cited study may reflect differences in analytical methods, as the specific muropeptide species analysed were not detailed. To avoid over-interpretation and potential confusion, we have chosen not to emphasise this comparison in the manuscript.

Importantly, we have repeated and expanded our PG analysis using an updated pipeline (PGFinder v1.4.0) with a broader search space. This more comprehensive analysis confirmed our original conclusions. As now stated in the revised text (lines 223-226), cefotetan exposure resulted in minimal structural changes, with no significant difference in overall crosslinking or the proportion of 4-3 crosslinks. Only a single low abundance trimeric muropeptide showed a substantial change, which may influence spore phenotype but cannot be directly attributed to *Cd*MecA.

We believe this cautious framing accurately reflects the data while acknowledging existing literature, and we appreciate the reviewer's suggestion, which prompted a more thorough re-analysis and clearer presentation.

I have a few concerns with the data analysis and the presentation of conclusions.

1. Line 41: Should this say, "*C. difficile* strains" rather than "*C. difficile* species"?

Line 41 has been corrected to say "*C. difficile* strains"

2. Line 42: "hijack" is an odd term to use here. This word generally means to take over by force and often with negative intent. This would seem to be a positive event that doesn't fully take over unless it is absolutely required.

Line 42 now says "Since SpoVD is conserved, MecA may broadly co-opt sporulation"

3. Line 44: The results in this paper are exciting and important, but it is not clear how this "reshapes our understanding of antibiotic resistance." It seems to be a direct repetition of a well-studied antibiotic resistance mechanism, simply placed into the new context of the sporulation process. "reshapes our understanding of antibiotic resistance" could be restated.

Old line 44 (line 45**)** now says "reshaping our understanding of antibiotic resistance within the context of sporulation"

4. Figure 2, panel A: I am not convinced that the right-hand part of this panel representing the amino acid alignment provides any useful information or is interpretable by the reader. It takes a lot of space here. A full alignment showing amino acids in Supplementary material could be more informative for those desiring that depth of inquiry.

We agree with the reviewer's suggestion. Panel A has now been split in two with the left-hand section retained in the main manuscript (Fig 2a) and the right-hand amino acid alignment moved to supplementary data (new Extended Data Fig 4).

5. Figure 3: The 2-fold difference in DPA between the strains (Panel D) is easily believable. This is hard to reconcile with the Raman difference (Panel E). If 80% of WT spores have NO DPA, as suggested by the Raman profile, then the difference in panel D should be greater. I suspect that:

A) Other differences between the WT and CdMecA+ spores result in an alteration of the Raman spectrogram, masking the detection of DPA in the WT spores;

B) The spores used in panel D and panel E are different, with those in Panel E having somehow lost their dehydration and DPA; OR

C) The Raman limit of detection is poor, resulting in lack off detection in many spores (least likely)

How can the authors explain the difference between the Panel D and E results? This should be addressed in the manuscript around lines 177-182.

We thank the reviewer for this careful observation and agree that the results in Figure 3 panels D and E should not be interpreted as directly quantitative comparisons.

Panel D measures total Ca-DPA content averaged across the entire spore population, whereas Panel E shows Raman spectra from individual spores and is intended as a qualitative, single-spore assessment. Interpreting the Raman data quantitatively can therefore give the misleading impression that most WT spores lack DPA. In reality, Raman signal intensity does not scale linearly with bulk DPA content.

Several factors explain the apparent discrepancy. First, Ca-DPA exhibits strong (pre-)resonance enhancement under 532-nm excitation, leading to pronounced band presence/absence rather than proportional intensity changes. Second, Raman signal intensity is highly sensitive to focal volume, spore

orientation, background subtraction, and strain-dependent differences in spore coat composition, all of which can mask DPA detection in individual WT spores despite retained bulk DPA. Finally, Panels D and E were performed on independent biological preparations generated at different times using the same protocol, which may contribute to variability in spore maturation and dehydration.

To clarify this point for readers, we have revised the manuscript (lines 206-207) to explicitly state that Raman spectroscopy was used as a qualitative single spore validation rather than a quantitative measure, and to avoid over-interpretation of the proportion of DPA-negative spores.

6. Line 667: Soluble muropeptides cannot be recovered by centrifugation. The insoluble spore debris can be removed by centrifugation, leaving the muropeptides in the supernatant.

This has been modified to say “Muropeptides were recovered in supernatants following centrifugation” (line 761).

7. In Data Set 2 about peptidoglycan structure, in "Groups and Summary", it is not at all clear what is meant by "Glycan Chains" versus "Monomers". The muropeptides in each group seem to include short sugar backbone fragments, frequently with non-crosslinked peptide side chains. How are the authors differentiating these two groups?

We thank the reviewer for raising this point and agree that the distinction between these categories was not sufficiently clear.

In Data Set 2, “glycan chains” refer to peptidoglycan fragments composed of disaccharides or longer glycan backbones (including tetrasaccharides), with or without a single attached peptide stem. These fragments arise from cleavage by an N-acetylmuramoyl-L-alanine amidase and therefore lack cross-linked peptide stems. In contrast, “monomers” are defined as disaccharide–peptides that contain at least one amino acid in the peptide stem.

We have now clarified these definitions directly in Data Set 2 (new Supplementary Extended Data 4) to avoid ambiguity, specifying: *glycan chains (disaccharides, tetrasaccharides, and tetrasaccharide peptides)* and *monomers (disaccharide peptides)*.

Reviewer #3 (Remarks to the Author):

I co-reviewed this manuscript with one of the reviewers who provided the listed reports. This is part of the Nature Communications initiative to facilitate training in peer review and to provide appropriate recognition for Early Career Researchers who co-review manuscripts.

Reviewer #4 (Remarks to the Author):

This manuscript entitled “Uncovering an alternate pathway of antibiotic resistance in spore-forming bacteria” presents a compelling and well-supported discovery of an alternative pathway of antibiotic resistance in Clostridioides difficile, mediated by the acquisition and activation of a MecA-homologue (CdMecA) that functionally replaces the sporulation-specific PBP SpoVD. The authors convincingly show that CdMecA enables sporulation under cephamycin pressure, produces phenotypically distinct spores with increased resilience, and relies on SpoVE/SpoVD interactions to function. They further extend this mechanism to Clostridium perfringens and propose a dual-antibiotic strategy to overcome resistance.

This is highly original work of broad interest. Several points, however, require clarification, improved justification, or additional data presentation.

Major Comments

- The manuscript states that cefotetan does not efficiently bind CdMecA based on competition fluorescence-polarization assays using CdMecAΔ1–34. While this is an important result supporting the central model, the corresponding experimental data are, unless I missed them, not shown anywhere in the manuscript. This is a major issue, as the strength of the conclusion (“cefotetan failed to displace Boc-FL from CdMecA even at 4000 μM”) cannot be evaluated without the presentation of these results. In addition, according to the Methods section, an essential control was performed: the ability of cefotetan to bind SpoVD, a known cephamycin-sensitive PBP. However, this positive control is not reported in the Results section. Demonstrating that cefotetan can displace Boc-FL from SpoVD is crucial to confirm that the assay is functioning properly and that the lack of displacement observed for CdMecA is meaningful.

We apologise to the reviewer for this oversight and agree that presentation of these data is essential to support our conclusions.

We have now included the fluorescence-polarization competition data demonstrating that cefotetan fails to displace Boc-FL from *Cd*MecA$_{\Delta 1\text{-}34}$, even at concentrations up to 4000 µM (new Extended Data Fig. 3a). This addition allows direct evaluation of the binding claim stated in the manuscript.

Regarding the required positive control, the ability of cefotetan to bind *Cd*SpoVD has been previously reported (PMID: 31406330; PMID: 31406331). For completeness and clarity, we have now repeated this assay and included the results alongside the *Cd*MecA data in the Supplementary Information (Extended Data Fig. 3b). As expected, cefotetan efficiently displaced Boc-FL from *Cd*SpoVD with nanomolar affinity (IC_{50} = 26.5 ± 10 nM), confirming that the assay is functioning correctly and validating the lack of displacement observed for *Cd*MecA.

These results are now explicitly described in the Results section (lines 127-130), strengthening the experimental support for our central conclusion that *Cd*MecA is refractory to cefotetan binding under these conditions.

-The analysis of MecA prevalence across spore-formers is not fully convincing. The reported candidates show only 26–35.6% nucleotide identity and 30.5–48.6% amino-acid identity to CdMecA, values that might be considered as too low to confidently infer homology or functional equivalence. To further strengthen this analysis, the authors could perform comparative AlphaFold2 structural predictions as in Extended data figure 2B. Structural conservation of the MecA domain and transpeptidase fold would support the classification of these proteins as true MecA homologues.

We thank the reviewer for this helpful suggestion and agree that sequence identity alone may be insufficient to confidently infer homology or functional equivalence.

In response, we performed comparative AlphaFold3 structural modelling of MecA candidates from the identified pathogenic and non-pathogenic spore-forming species and aligned both the MecA regulatory domain and the transpeptidase domain against the *C. difficile* MecA AlphaFold3 model. Despite modest sequence identity (26-35.6% nucleotide; 30.5-48.6% amino acid), these analyses revealed highly conserved three-dimensional folds across all models. The average RMSD was 1.9 Å for the MecA domain and 1.6 Å for the transpeptidase domain, indicating strong structural conservation.

These results provide independent structural support that the identified proteins are MecA homologues, reinforcing the prevalence analysis across spore-formers. We have added this information to the manuscript (lines 168-171), with representative structures shown in Extended Data Fig. 5.

- In addition, the manuscript does not provide any functional validation showing that these distant MecA orthologues behave as CdMecA and enable sporulation in the presence of cefotetan. To strengthen their conclusions, the authors should consider possible functional validation strategies. For example, comparing sporulation levels in the presence or absence of cefotetan in a Clostridium perfringens or Bacillus subtilis strain carrying a putative MecA homologue versus a closely related non-MecA strain. Alternatively, the authors could express a mecA candidate in a C. difficile strain lacking mecA or in the C. difficile mecA knockout strain to determine whether it restores cefotetan-associated sporulation resistance.

We agree that functional validation is important and note that such experiments have already been performed in *C. difficile*. Specifically, we expressed a *mecA* construct in trans in two independent *C. difficile* backgrounds lacking endogenous *mecA*: (i) strain M7404, which naturally lacks *mecA* (Fig. 1d), and (ii) the JGS6133 Δ*mecA* mutant (Fig. 1c). In both cases, introduction of *mecA* resulted in a partial restoration of sporulation in the presence of cefotetan, demonstrating that MecA is functionally capable of mitigating cefotetan-mediated inhibition of sporulation.

The rescue is partial, as expected, because *mecA* was supplied *in trans* rather than restored chromosomally. Nevertheless, these data provide direct functional evidence that MecA activity is sufficient to confer cefotetan-associated sporulation tolerance in a *C. difficile* background.

While extending this analysis to heterologous spore-formers (e.g. *Clostridium perfringens* or *Bacillus subtilis*) would be informative, such experiments introduce additional complexity due to species-specific sporulation pathways and are beyond the scope of the current study. Together, the existing complementation data support the functional relevance of MecA homologues in the context tested.

- The regulatory aspects of the mec locus require further explanation. First, unlike S. aureus MecI, which represses mecA, disruption of CdmecI does not increase MecA production in the absence of antibiotics (Extended Data Fig. 2f). This unexpected result should be discussed. Does CdMecI not function as a repressor? Could other regulatory mechanisms mask its effect? Second, the authors report that CdmecI and CdmecR are frequently present in strains lacking CdmecA. The biological significance of this is unclear. Are these regulatory genes remnants of a formerly complete mec locus? Do they regulate another gene in these strains? It would greatly strengthen the manuscript to examine in more detail the genomic context of

mecI/mecR in several mecA-negative strains to determine whether another gene or operon could plausibly be under the control of this system.

We thank the reviewer for these important points and agree that the regulatory aspects of the *mec* locus warrant clarification.

First, we acknowledge that inactivation of *CdmecI* did not lead to increased *Cd*MecA production in the absence of antibiotics (new Extended Data Fig. 2f). While this contrasts with the canonical *Staphylococcus aureus* model, it is not entirely unexpected. In *S. aureus*, MecI and the closely related β-lactam repressor BlaI are functionally interchangeable and can cross-repress cognate promoters (PMID: 11698375). Notably, *C. difficile* strain JGS6133 encodes at least seven annotated MecI/BlaI-like repressors. It is possible that in the absence of *Cd*MecI, repression of *CdmecA* may be maintained by one or more alternative repressors, masking the effect of *CdmecI* disruption.

Second, we agree that the frequent presence of *CdmecI* and *CdmecR* in strains lacking *CdmecA* is intriguing. While this raises the possibility that these regulators are remnants of a formerly complete *mec* locus or may control alternative targets, detailed analysis of their genomic context and regulatory scope lies beyond the focus of the current manuscript. Our study specifically aimed to define the regulatory system in the context of *CdmecA* function. We note this open question as an important avenue for future research and indicate that it forms the basis of ongoing follow-up work in our group examining the *Cdmec* system in diverse *C. difficile* backgrounds.

We have revised the manuscript to better frame these observations, acknowledge the limitations, and avoid over-interpretation while highlighting key directions for future investigation.

- In Fig. 5, dual treatment with cefotetan and ceftobiprole "abolished sporulation," whereas cefotetan alone in the mecA mutant only strongly reduces, but does not eliminate, spore production. The manuscript would benefit from clarification of why a complete suppression of sporulation is observed in one case but only partial suppression in the other, e.g., differences in antibiotic concentration, timing, culture density, or quantification methods.

We thank the reviewer for raising this important point and agree that clarification is warranted.

The complete suppression of sporulation observed with dual treatment (cefotetan + ceftobiprole) likely reflects additive or synergistic inhibition of distinct but complementary steps in the spore peptidoglycan synthesis pathway. Cefotetan inhibits SpoVD dependent transpeptidation, while ceftobiprole targets *Cd*MecA; together, this simultaneously blocks both compensatory activities. In contrast, treatment of the *mecA* mutant with cefotetan alone strongly reduces, but does not fully eliminate, sporulation, likely due to residual or alternative low level pathways supporting minimal spore formation.

This interpretation is supported by additional data showing that cefotetan combined with ceftaroline similarly abolishes sporulation (new Extended Data Fig. 11), indicating that complete suppression is a reproducible outcome of dual PBP inhibition rather than an experimental artefact. All experiments were performed using the same antibiotic concentrations, timing, culture conditions, and quantification methods, ruling out methodological differences as the cause.

We have clarified this rationale in the manuscript to explain why dual treatment produces complete inhibition whereas single agent treatment in the *CdmecA* mutant does not (lines 312-319).

Minor comments

- The authors report that sporulation in strains belonging to Clades 4, 5, and C-III is not inhibited by cefotetan, and later note that CdmecA is absent from all C-III isolates. This observation implies that CdmecA alone cannot account for the cefotetan-insensitive sporulation phenotype across all lineages. While it is entirely plausible that additional, lineage-specific mechanisms exist, this should be explicitly acknowledged in the text to avoid over-attributing this phenotype to MecA.

We thank the reviewer for raising this important point and agree that clarity is essential to avoid over attribution of cefotetan insensitive sporulation to *Cd*MecA alone.

To clarify, the C-III strain used in our sporulation assays (Fig. 1a,b) does encode *CdmecA*, and the observed cefotetan-insensitive sporulation in this case is therefore consistent with a role for *Cd*MecA. The C-III isolates listed in Supplementary Table 4 that lack *CdmecA* were not the strains examined experimentally in Fig. 1a,b; those isolates were identified through genomic surveys only and do not impact the interpretation of the sporulation data presented. The experimentally tested C-III strain has an unknown ST/RT and was therefore not included in the prevalence analysis shown in Supplementary Table 4.

That said, we agree with the reviewer's broader point that *Cd*MecA alone may not account for cefotetan insensitive sporulation across all lineages and that additional, lineage specific mechanisms are plausible. We have now explicitly acknowledged this in the revised manuscript to make clear that while *Cd*MecA is sufficient in the strains tested here, other mechanisms likely contribute to cefotetan tolerance in *mecA* negative lineages.

We appreciate the reviewer highlighting this nuance and have revised the text accordingly to avoid over generalisation.

- At line 111, the authors conclude that CdMecA's role is "specific to sporulation, not vegetative cell survival." This is misleading, given that the manuscript later notes (line 271) that CdMecA also confers β-lactam resistance in vegetative cells (as shown elsewhere). I recommend rephrasing this conclusion more precisely to indicate that the sporulation-specific role is observed in the context of cephamycin exposure, and that CdMecA has broader functions under different β-lactam conditions.

We thank the reviewer for their suggestions. Line 116 now sates: "…confirming that *Cd*MecA's role in the context of cephamycin exposure, is specific to sporulation, not vegetative cell survival. We have not included any additional commentary about *Cd*MecA having broader functions on vegetative cells under different β-lactam conditions as this section (lines 102-112) is related specifically to cephamycin-mediated sporulation inhibition.

- Line 119 states that CdmecA is "part of an operon" with mecR and mecI. However, mecA is oriented divergently relative to mecR/mecI, which is inconsistent with a single operon. This sentence should be revised to accurately reflect the transcriptional organization.

We agree that the use of the term "operon" was incorrect. The text (see line 131-132) has been revised to state "Like *S. aureus mecA*, *CdmecA* is encoded in a *mec* gene complex comprised of *CdmecA* and convergently transcribed regulatory genes *CdmecR* (antibiotic sensor) and *CdmecI* (antibiotic repressor) (Extended Data Fig. 2a). The term "mec operons" was also replaced with "mec gene complex" in the Extended Data Fig. 2 figure legend.

- Including the mecA mutant treated with cefotetan as a control in the spore-resilience experiments in Fig. 3 strengthens the interpretation that increased resistance is associated with MecA-dependent sporulation rather than with the presence of the antibiotic. However, Fig. 1 shows that this mutant produces extremely few spores under cephamycin exposure (~4×10^2 spores/mL). It is therefore unclear how sufficient material was obtained for purification and downstream assays. The authors should clarify whether spores used in Fig. 3 were harvested under identical conditions as those quantified in Fig. 1.

The spores generated for the resilience experiments (Fig 3) were isolated using a different published protocol optimised for spore purification which differs from the sporulation assay protocol (Fig 1). The spore purification protocol involves growing strains on plates for several days (with and without antibiotics). Cefotetan-treated *CdmecA* mutant cultures produced fewer spores per plate compared to the untreated *CdmecA* mutant. To obtain sufficient material we increased the number of plates while keeping growth conditions otherwise identical. This ensured comparable quantities of purified spores across all groups used in Fig 3 assays.

- Extended Data Fig. 4 uses several acronyms that may be unfamiliar to non-specialists. These should be defined directly in the figure legend.

The following has been added to extended data 4 (new supplementary extended data Fig 7) figure legend.

Note: g, GlcNAc; m, MurNAc; g(-Ac), GlcN; A, alanine; E, Glutamic acid; J, meso-diaminopimelic acid; M, methionine; G, glycine; "=" indicates a cross link between two disaccharide peptide.

- Supplementary Tables 7 and 8 are not referenced in the main text. All supplementary materials should be explicitly cited at least once to clarify their relevance and guide readers.

We thank the reviewer for identifying that the supplementary tables were not referenced in the main text. These have now been included in line 219 "…composition using LC-MS and PGFinder (New Supplementary Extended data 2-3)".

Reviewer’s Comments #2

Reviewer #1 (Remarks to the Author):

In the reviewer's opinion, the authors have conscientiously responded to critiques raised. I would recommend the authors consider adding to the discussion, only relevant information that can be obtained from their response to critique 1 "Taken together, these findings suggest that although CdMecA is not currently associated with increased disease burden, its presence in emerging and environmentally prevalent clades supports the need for continued genomic surveillance. Monitoring these lineages is important to detect early shifts in pathogenicity or resistance that may impact clinical and public health outcomes."

The following sentence has been added to the discussion “These findings suggest that although *Cd*MecA is not currently associated with increased disease burden, its presence in emerging and environmentally prevalent clades supports the need for continued genomic surveillance. Monitoring these lineages is important to detect early shifts in pathogenicity or resistance that may impact clinical and public health outcomes.”

Reviewer #2 (Remarks to the Author):

The authors have adequately addressed my concerns from the initial review.

Reviewer #4 (Remarks to the Author):

The authors have thoroughly and satisfactorily addressed all of my previous concerns. The revised manuscript is substantially strengthened, and the additional data, clarifications, and textual revisions fully resolve the points I raised.

I have no further comments. I am satisfied with this new version of the manuscript and with the work carried out by the authors.

I support publication of the manuscript in its revised form.
