# Supplementary material for: Uncovering an alternate pathway of antibiotic resistance in spore-forming bacteria
Source: Nat Commun. 2026 Jul 15;17:8674. doi: 10.1038/s41467-026-75594-5 (PMC13490422; doi:10.1038/s41467-026-75594-5)
Supplement: Supplementary file 3 [file 41467_2026_75594_MOESM1_ESM.pdf]

Uncovering an alternate pathway of antibiotic resistance in spore-forming bacteria.

Yogitha N. Srikhanta[1,2*], Clara E. Bate[1,2*], Desirel Ng[1,2], Sarah A. Revitt-Mills[1,2], Georgia-Rose Gilmore[1,2], Galain C. Williams[1,2], Sophie L. Day[1,3], Stéphane Mesnage[4], Kamila Kochan[2,5], Shailab Shrestha[6,7], Aimee Shen[6], Daniel R. Knight[8,9], Korakrit Imwattana[9,10], Thomas V. Riley[8,9], Irene Alevizos[1,2], Kimberley Bourke[1], Milena M. Awad[1,2], Caroline A. Evans[11], Ghizal Siddiqui[12], Joel R. Steele[13], David L. Steer[13], Joshua P. Morrow[12], Darren J. Creek[12], Chaille Webb[1], Sheena McGowan[1], and Dena Lyras[1,2#]

[1]Infection Program, Monash Biomedicine Discovery Institute and Department of Microbiology, Monash University, Clayton, Victoria 3800, Australia.

[2]Centre to Impact AMR, Monash University, Melbourne, VIC 3800, Australia

[3]Barwon Health University Hospital, Bellerine St, Geelong, Victoria 3220, Australia.

[4]Molecular Microbiology, School of Biosciences, University of Sheffield, S10 2TN Sheffield, UK.

[5]School of Chemistry, Faculty of Science, Monash University, Clayton, Victoria 3800, Australia.

[6]Tufts University School of Medicine, Department of Molecular Biology and Microbiology, 136 Harrison Avenue, Boston, MA, 02111, USA.

[7]Program in Molecular Microbiology, Tufts University Graduate School of Biomedical Sciences, Boston, MA, 02111, USA.

[8]Department of Microbiology, PathWest Laboratory Medicine, Queen Elizabeth II Medical Centre, Western Australia, Australia.

[9]School of Biomedical Sciences, The University of Western Australia, Western Australia, Australia.

[10]Department of Microbiology, Faculty of Medicine Siriraj Hospital, Mahidol University, Thailand.

[11]School of Chemical, Materials and Biological Engineering, University of Sheffield, Sheffield, UK.

[12]Drug Delivery, Disposition and Dynamics, Monash Institute of Pharmaceutical Sciences, Monash University, Parkville, VIC 3052, Australia.

[13]Monash Proteomics & Metabolomics Platform, Department of Biochemistry and Molecular Biology, Biomedicine Discovery Institute, Monash University, Clayton, VIC, 3800, Australia.

[*]These authors contributed equally to the manuscript

[#]Correspondence and requests for materials should be addressed to: Dena Lyras (Email: Dena.Lyras@monash.edu)

Supplementary Notes 1

CdmecA is regulated by *CdmecI* and *CdmecR*.

SamecA is encoded within a mobile gene complex known as SSC*mec,* adjacent to the regulatory genes *mecI* (transcriptional repressor preventing *Sa*MecA expression in the absence of β-lactams) and *mecR1* (antibiotic sensor responsible for sensing β-lactams) (Supplementary Fig. 2a; [1]). Comparison of the *mecA*-encoding regions from *S. aureus* strain N315 and *C. difficile* JGS6133 determined that *CdmecA* is encoded within a similar gene complex, alongside *CdmecR* and *CdmecI* (Supplementary Fig. 2a). AlphaFold2 models of *Cd*MecI and *Cd*MecR demonstrate conserved folds similar to the *S. aureus* homologues (Supplementary Fig. 2c, Supplementary Table 2), suggesting that they function in a similar manner. The presence of these regulatory elements suggests that *Cd*MecA regulation functions in a β-lactam inducible manner similar to *S. aureus* [2,3]. To confirm this hypothesis, we constructed JGS6133 *CdmecR* and *CdmecI* mutants and examined sporulation in the presence and absence of cefotetan. The *CdmecR* mutant strain was unable to sporulate in the presence of cefotetan while the absence of *mecI* had no effect on sporulation in the presence of cefotetan (Supplementary Fig. 2d). Vegetative cell numbers were unaffected (Supplementary Fig. 2d). PBP profiling showed that *CdmecA* was absent in the cefotetan-treated *CdmecR* mutant membrane proteome but present in the cefotetan-treated *CdmecI* mutant (Supplementary Fig. 2e). These findings suggest that *CdmecR* induces *Cd*MecA expression as disruption impaired cefotetan-induced *Cd*MecA expression, leading to abolished sporulation, while *CdmecI* represses *Cd*MecA expression as disruption did not alter *Cd*MecA expression or sporulation.

Supplementary Notes 2

***CdmecA*, *CdmecI* and *CdmecR* are distributed across *C. difficile* species.**

The distribution of *CdmecAIR* across *C. difficile* species was then examined, by screening a characterised global collection of 10,330 *C. difficile* genomes encompassing 270 multilocus sequence types (STs) spanning all Clades [4]. *CdmecA* was present in 9.8% (1,017/10,330) of sequenced genomes and in 17.0% (46/270) of STs (Supplementary Data 1, Supplementary Table 4). The distribution of *CdmecA* varied considerably (Clade 5 (55.5%), Clade 4 (40.8%), Clade 1 (4.7%), and Clades 2, 3, C-I, C-II, and C-III (0-0.2%) (Supplementary Table 4). Notably, *CdmecA* is found in every sequenced genome belonging to 22 different STs in 3 Clades (Supplementary Table 5, Supplementary Data 1). Of the isolates carrying *CdmecA*, only two isolates were found not to encode *CdmecI* and *CdmecR* (Supplementary Data 1). *CdmecI* and *CdmecR* were found amongst all Clades, even in the absence of *CdmecA* (86.8%; Supplementary Table 4, Supplementary Data 1).

Supplementary Notes 3

MecA is found amongst a diverse range of pathogen, gut and environmental spore-formers.

The phylogeny of *mecA* from species across four different spore-forming orders was reconstructed through a maximum likelihood phylogenetic tree, rooted against the non-spore forming *Mammaliicoccus sciuri.* The resulting tree (Fig. 2a) contained three major branch points that largely recapitulated the taxonomic classification of the analysed species, with some notable exceptions. Interestingly, *Clostridium perfringens mecA* was placed amongst the *Bacillaceae*, in a small clade that included *Bacillus safensis* and *Nialla circulans,* indicative of *mecA* horizontal gene transfer between these isolates. Given the placement of *C. perfringens* within the *Bacillaecae* clade, we suggest *C. perfringens* was likely the recipient of this transfer event. Notably, the Eubacteriales order did not form a monophyletic clade as observed with the *Bacillaecae*. Instead, *Clostridium disporicum, Paeniclostridum sordellii*, and *Clostridium sporogenes* formed a smaller, well-supported cluster with *Tauribacter* spp., which are members of the Erysipelotrichales. This suggests horizontal gene transfer has occurred amongst these isolates, though the direction of transfer was less evident. The resulting tree was also considered in context with a multiple sequence alignment of encoded MecA proteins, which indicated high levels of residue conservation across the MecA and transpeptidase domains, except for *Mahella australensis,* which included a unique insertion of 10 amino acids within the transpeptidase domain (Supplementary Figure 5). Collectively, these findings indicate that MecA is present across diverse spore-forming bacterial species and in some instances may have undergone horizontal gene transfer between disparate species.

Supplementary Notes 4

Raman Spectroscopy shows differences in chemical properties between *Cd*MecA and *Cd*SpoVD spores.

Raman spectra (Fig. 3e) acquired from 52 intact *Cd*SpoVD spores exhibited similar profiles, with multiple bands. Some of these, e.g. at 1652 cm^{-1} (amide I, proteins), are present in most biological materials, including spores and vegetative cells. For *Cd*MecA spores, in addition to 'typical' protein and lipid bands, the spectra contained distinct bands at 661, 826, 1017, 1396, 1443, and 1573 cm^{-1}, attributed to Calcium Dipicolinate (Ca-DPA). The Ca-DPA specific bands were found in 43/52 *Cd*MecA cells, and only in 1/52 *Cd*SpoVD cells.

Supplementary Notes 5

Comparative analysis of *Cd*SpoVD and *Cd*MecA peptidoglycan structure.

Eighty-three structures were identified across both spore types, revealing an unprecedented level of complexity (Supplementary Data 2). No major difference was observed in the crosslinking index between *Cd*SpoVD and *Cd*MecA spores, which remained low (3.85% ± 0.41% and 3.90% ± 0.43%, respectively). One muropeptide showed a 5-fold change in abundance (Supplementary Figure 7).

Supplementary Notes 6

Western Blot and Coomassie stain of His-tagged SpoVD confirms no non-specific binding to Strep-Tactin resin.

His-tagged recombinant SpoVD was incubated with Strep-Tactin before being washed and elution buffer applied. Samples were taken from the protein prior to incubation with Strep-Tactin (input) and the protein eluted (elution). These samples were run on SDS-PAGE gels and then used for Western blot or Coomassie stained (Supplementary Fig. 9f-g). Coomassie stained gels and Western blot shows SpoVD was only found in the input sample and therefore shows that there is no non-specific binding of SpoVD to Strep-Tactin. From this we can conclude that Strep-II-tagged MecA can pull out His-tagged SpoVD.

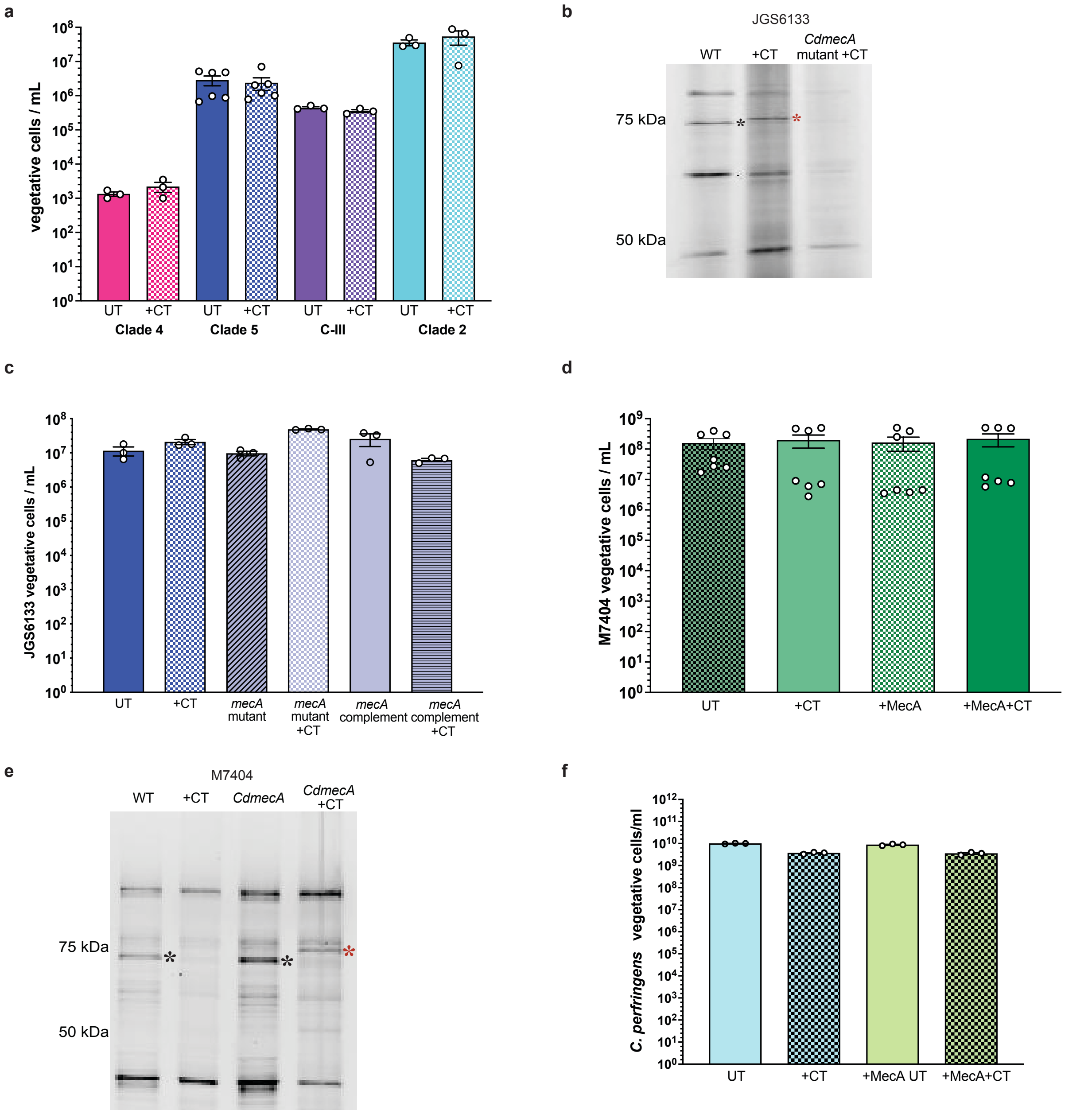

Supplementary Figure 1. Effect of cefotetan on *C. difficile* MecA^{+} and *C. perfringens* MecA^{+} vegetative cell numbers and confirmation of *CdmecA* absence in the JGS6133 *CdmecA* mutant. a, Using sporulation assays, vegetative cell numbers (vegetative cells/mL) were determined for cultures from strains MA0129 (clade 4), JGS6133 (clade 5), and A3491 (clade III) which were either untreated or cefotetan-treated. M7404 (clade 2) was used as a control. **b,** To

confirm that absence of MecA in the *mecA* mutant, membrane proteins from sporulating wild-type JGS6133 untreated or cefotetan-treated and cefotetan-treated *CdmecA* mutant cultures, were isolated, incubated with Boc–FL and separated by SDS–PAGE. Labelled PBPs were visualized using a FluorImager. Black asterisks indicate *Cd*SpoVD which is visualized in untreated cultures but not visualized in the cefotetan-treated wild-type and *CdmecA* mutant cultures (*Cd*SpoVD bound to cefotetan and therefore can't bind to Boc-FL), red asterisks indicate *Cd*MecA which is visualized in cefotetan-treated wild-type cultures but not in untreated wild-type (absence of cefotetan to induce *Cd*MecA expression) and cefotetan-treated *CdmecA* mutant cultures (absence of *Cd*MecA thus not induced by cefotetan). **c,** Vegetative cell numbers of sporulating JGS6133 untreated and cefotetan-treated wild-type, *CdmecA* mutant and *CdmecA* complemented cultures were determined. **d,** Vegetative cell numbers of sporulating M7404 untreated and cefotetan-treated wild-type, *CdmecA* mutant and *CdmecA* complemented were determined. **e,** Membrane proteins from sporulating wild-type M7404 and MecA^{+} cultures, left untreated or treated with cefotetan were isolated, incubated with Boc–FL and separated by SDS–PAGE. Labelled PBPs were visualized using a FluorImager. Black asterisks indicate *Cd*SpoVD which is visualized in untreated cultures but not visualized in the cefotetan-treated wild-type and MecA^{+} cultures, red asterisks indicate *Cd*MecA which is visualized in cefotetan-treated wildtype and MecA^{+} cultures but not in untreated wild-type and MecA^{+} cultures. **f,** Vegetative cell numbers of sporulating *C. perfringens* DLL6642 untreated and cefotetan-treated wild-type cultures were compared to untreated and cefotetan-treated +MecA cultures. UT=untreated; +CT=cefotetan-treated; +MecA=*CdmecAIR*$^{+}$. For sporulation assays, results are the average of three-seven independent biological replicates. Error bars represent s.e.m.; P values were determined by using a Kruskal-Wallis test with Dunn's

pairwise comparisons or by one-way ANOVA. No statistical differences in vegetative cell counts were seen between any of the samples. Source data are provided as a Source Data file.

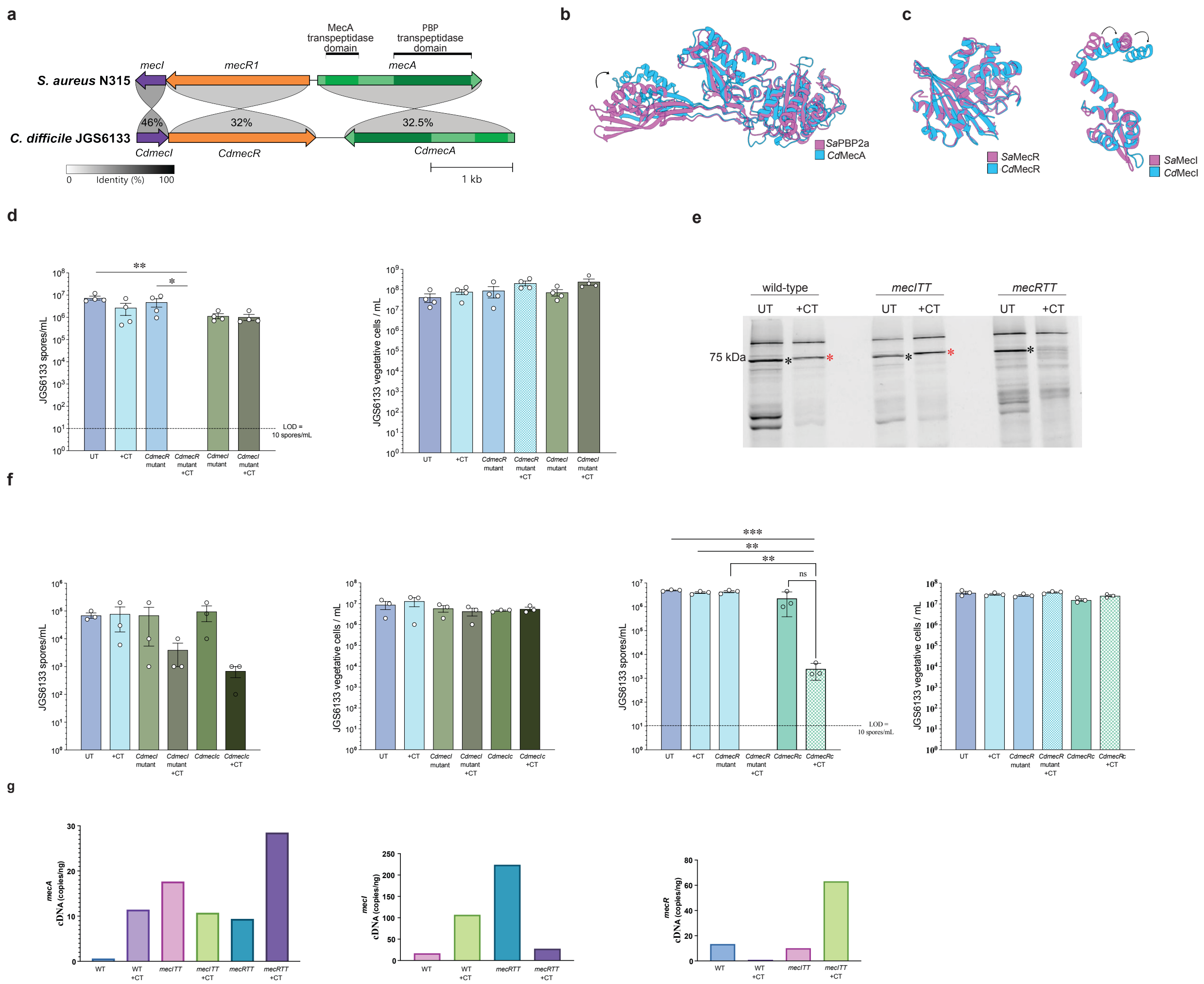

Supplementary Figure 2. Identification of *Cd*MecA and its regulatory elements *Cd*MecR and *Cd*MecI and the effect of *Cd*MecR and *Cd*MecI on *C. difficile* sporulation. a, Schematic representation of the *S. aureus* and *C. difficile mec* gene complex. *S. aureus*, *SamecA* (green arrow) is located on a mobile genetic element SCC*mec*, alongside regulatory genes *SamecI* and *SamecR1* (purple and orange arrows respectively). This genetic organisation is comparable to *C. difficile*, which also possesses homologues of *CdmecI* and *CdmecR* adjacent to *CdmecA*, which share 45%, 32%, and 33.5% amino acid identity with *SamecI, SamecR1* and *SamecA* respectively. Image produced using Clinker [5]. **b,** *Cd*MecA is predicted to be structurally similar to *S. aureus* PBP2a.

Superimpositions of AlphaFold2 [6] derived models of *C. difficile* JGS6133 MecA38-692 with experimentally determined X-ray crystal structures of *Sa*MecA (PDB ID:1VQQ [7]). *Cd*MecA (blue) and *Sa*MecA (pink). Black arrow indicates area where the AlphaFold prediction differs from the published crystal structures. **c,** *Cd*MecI and *Cd*MecR are predicted to be structurally similar to *S. aureus* homologues. Superimpositions of AlphaFold2 [6] derived models of *C. difficile* JGS6133 MecR246-599 and MecI with experimentally determined X-ray crystal structures of *S. aureus* MecR sensor domain (PDB ID: 6O9S [8]) and MecI (PDB ID: 1SD6 [9]). *C. difficile* coloured blue and *S. aureus* coloured pink. Black arrows indicate areas where the AlphaFold prediction differs from published crystal structures. **d,** JGS6133 sporulation assays were performed comparing spore numbers between untreated and cefotetan-treated wild-type, *CdmecR* mutant and *CdmecI* mutant cultures. No significant difference was seen between untreated and cefotetan-treated wild-type and *CdmecI* mutant spore numbers (*P*=0.69). Sporulation was completely abolished in the cefotetan-treated *CdmecR* mutant (*P*=0.029). Vegetative cell counts were determined. No statistical difference in vegetative cell counts was seen between any of the samples. **e,** Sporulating JGS6133, *CdmecI* mutant and *CdmecR* mutant cultures were left untreated or cefotetan-treated before isolation of membrane proteins. Isolated proteins were incubated with Boc–FL and separated by SDS–PAGE. Labelled PBPs were visualized using a FluorImager. Black asterisks indicate *Cd*SpovD which is visualized in all untreated cultures but not visualized in cefotetan-treated cultures (*Cd*SpoVD bound to cefotetan and therefore can't bind to Boc-FL), red asterisks indicate *Cd*MecA which is visualized in cefotetan-treated cultures but not in untreated cultures (no cefotetan present to induce expression of *Cd*MecA) and cefotetan-treated *CdmecR* mutant cultures. **f,** JGS6133 *mecI* and *mecR* mutant complementation sporulation assays were performed comparing spore numbers between untreated and cefotetan-treated wild-type vector control,

CdmecI mutant vector control, *CdmecI* mutant complemented, *CdmecR* mutant vector control and *CdmecR* mutant complemented cultures. No significant differences were seen between untreated and cefotetan-treated wild-type, *CdmecI* mutant and *CdmecI* mutant complemented spore numbers (*P*=0.6327->0.999). A significant difference was seen between untreated wild-type and cefotetan-treated *CdmecR* mutant complemented (*P*=0.0008), cefotetan-treated wild-type and cefotetan-treated *CdmecR* mutant complemented (*P*=0.0040) and untreated *mecR* mutant and cefotetan-treated *CdmecR* mutant complemented (*P*=0.0020). No significance was seen between the other cultures including untreated and cefotean-treated *CdmecR* mutant complemented cultures (*P*=0.0933). For sporulation assays, results are the average of three-four independent biological replicates Vegetative cell counts were also determined, with no statistical difference seen between any of the samples. UT=untreated, +CT=cefotetan-treated. For sporulation assays, results are the average of three-four independent biological replicates. Error bars represent s.e.m.; P values were determined by using a Kruskal-Wallis test with Dunn's pairwise comparisons or one-way ANOVA. Limit of detection (LOD) shown as a horizontal dotted line. **g,** Digital (dPCR) analysis of *C. difficile* JGS6133 wildtype (WT), *mecI* mutant (*mecITT*) and *mecR* mutant (*mecRTT*) strains after 24 h growth with and without cefotetan. dPCR was performed on a representative biological samples (n=1) using cDNA produced after RNA extraction and appropriate primers to detect copies/ng of *mecA, mecI* and *mecR*. Source data are provided as a Source Data file.

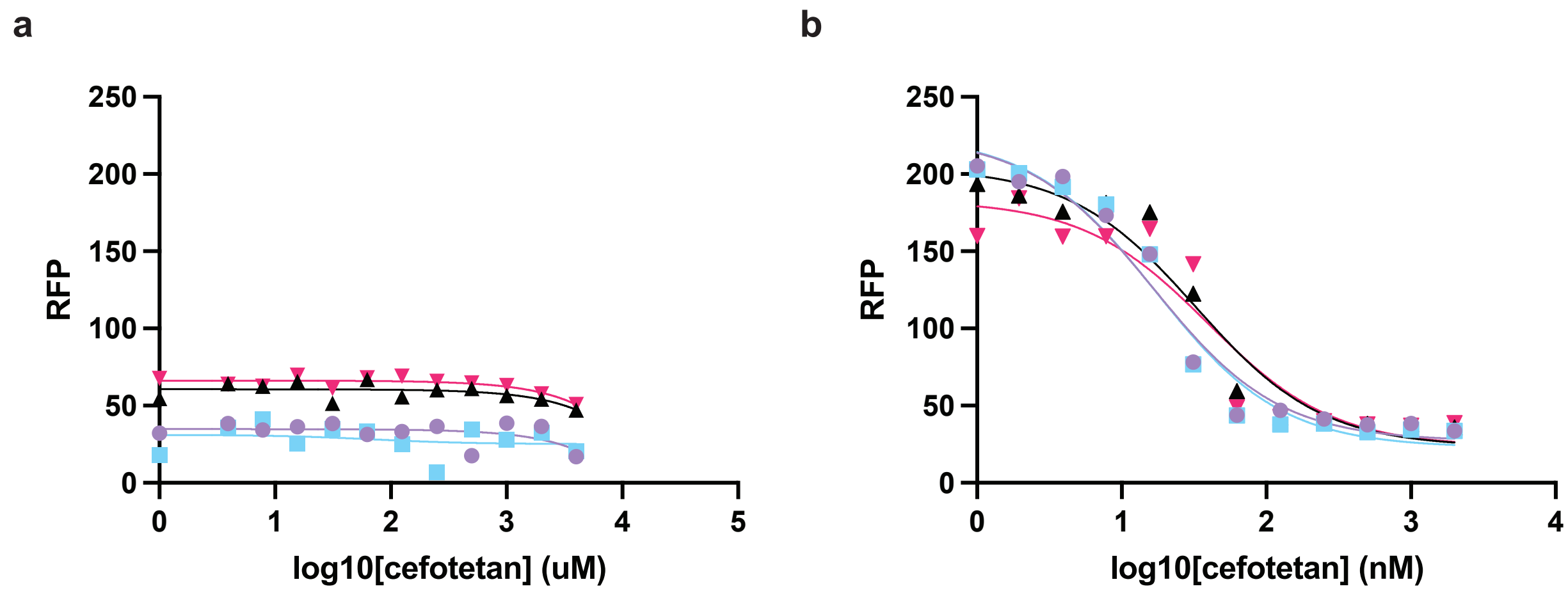

Supplementary Figure 3. Cefotetan binds *Cd*SpoVD with nanomolar affinity but not *Cd*MecA. Fluorescence polarisation assays of cefotetan binding **a,** *Cd*MecA and **b,** *Cd*SpoVD. Each assay was performed in biological and technical duplicate. Source data are provided as a Source Data file.

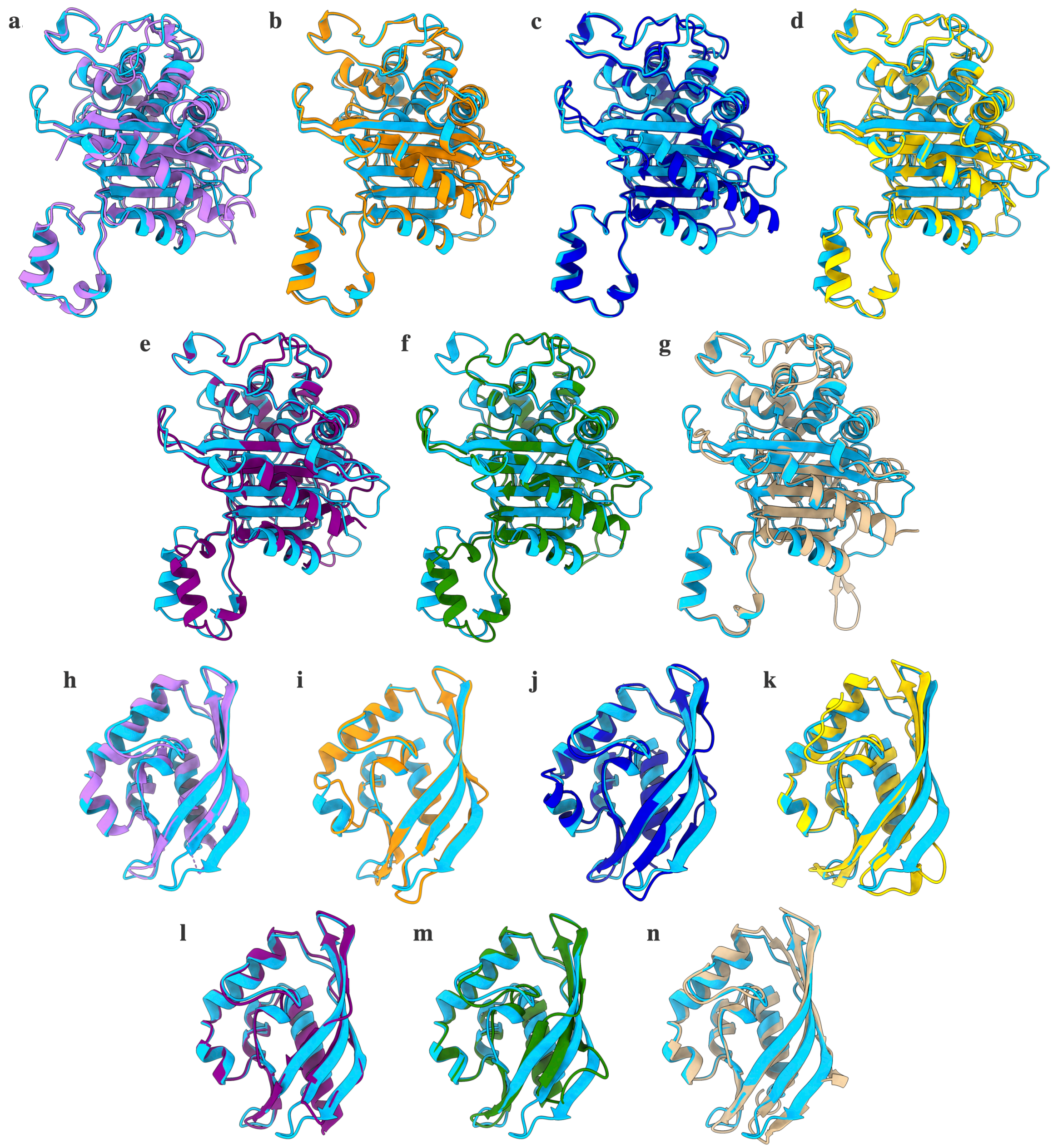

Supplementary Figure 4. AlphaFold3 predictions of representative MecA proteins from spore-former pathogens, gut microbes, and environmental isolates. Alignment of the **a–g, transpeptidase domain** and **h–n,** MecA domain of *Cd*MecA (blue) with *S. aureus* PBP2a (purple, PDB: 1MWR), or MecA proteins from *B. wrexlae* (orange), *N. circulans* (dark blue), *B. amyloliquefaciens* (yellow), *T. bilis* (dark purple), *P. sordellii* (green), and *M. australensis* (beige).

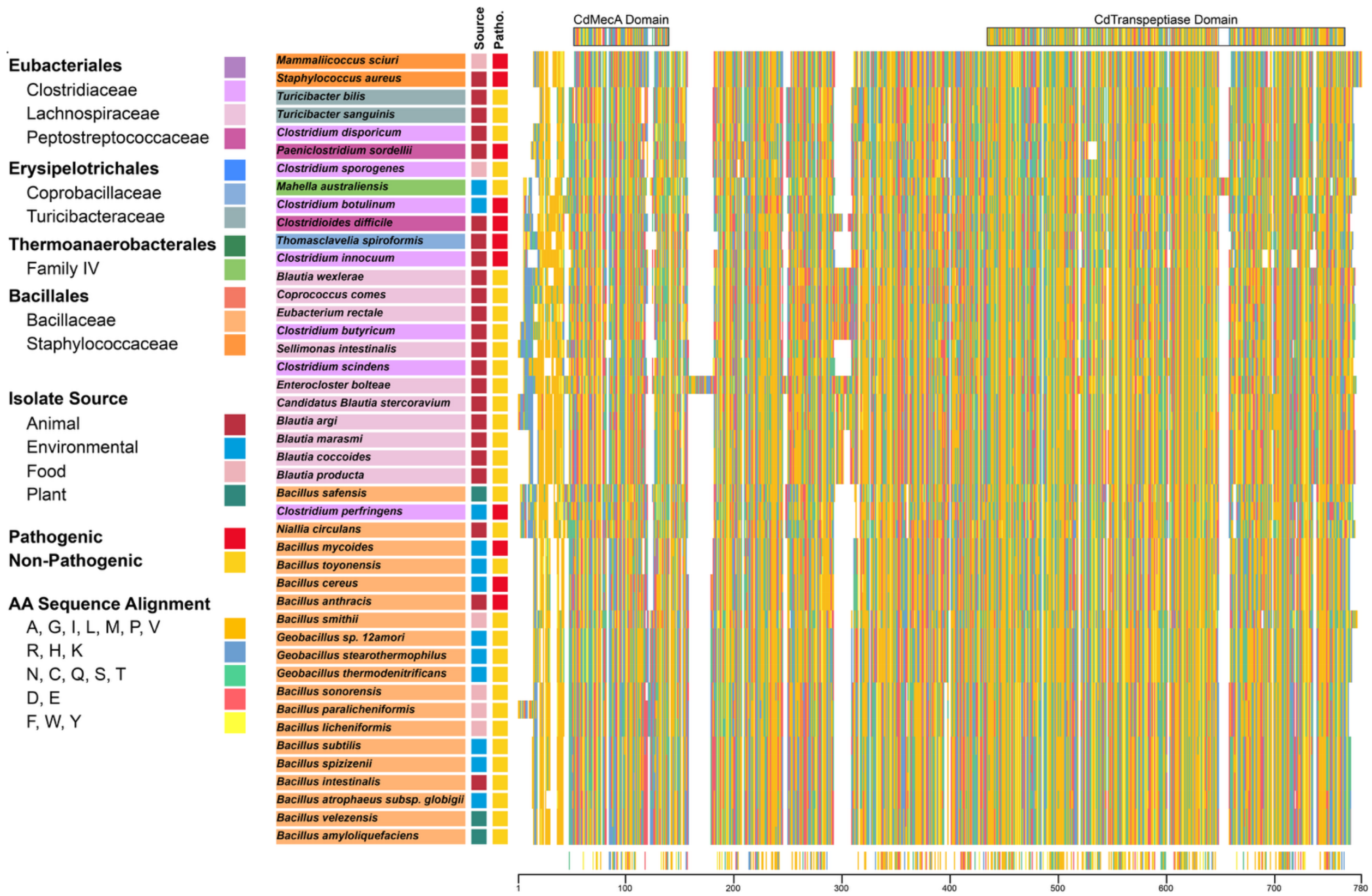

Supplementary Figure 5. Multiple sequence alignment of MecA across spore and non-spore forming clades. Visualisation of a clustal omega amino acid multiple sequence alignment of MecA in iTOL V6. Outlined boxes represent the MecA and transpeptidase domains from *C. difficile*. Residues below the multiple sequence alignment represent the 50% consensus residue. Source data are provided as a Source Data file.

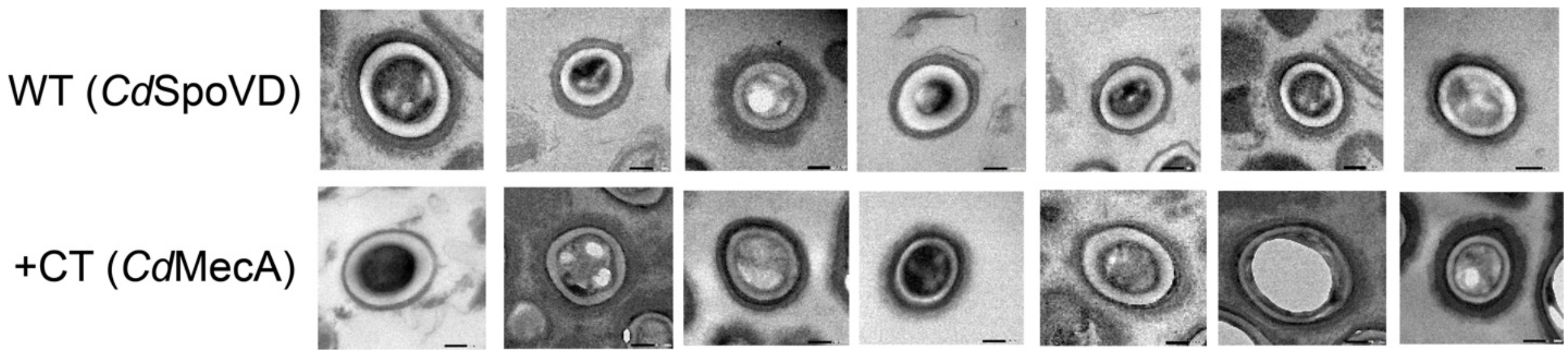

Supplementary Figure 6. Representative TEM images of wild-type *Cd*SpoVD and cefotetan-induced (+CT) *Cd*MecA spores. Black line represents 200 nm.

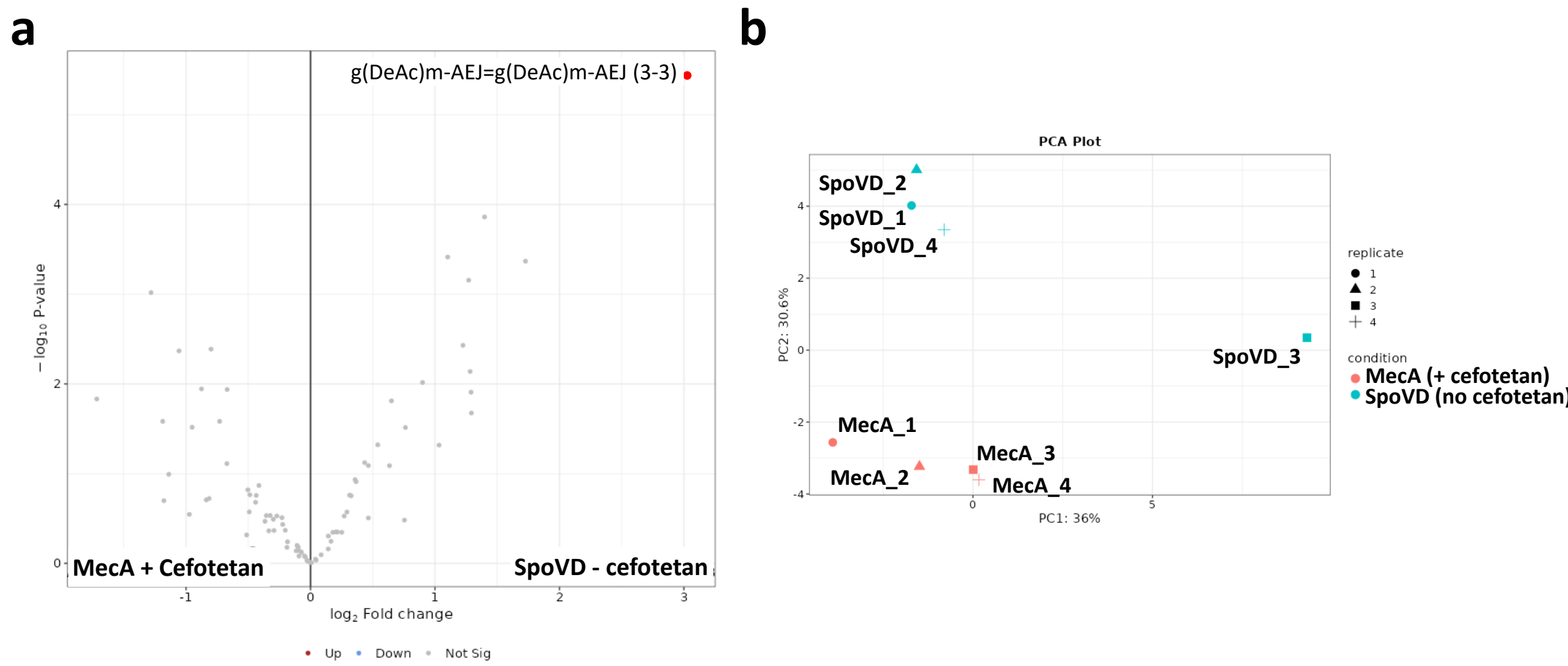

Supplementary Figure 7. Comparative analysis of *Cd*MecA (+cefotetan) and *Cd*SpoVD (no cefotetan) PG composition. a, Volcano plot, where each dot represents an individual muropeptide, plotted against the significance (*P* value<0.01) and difference (log_2 fold change ≥ 2.322). Muropeptides showing a significantly different abundance between two conditions are highlighted by a dark black dot. Note: g, GlcNAc; m, MurNAc; g(-Ac), GlcN; A, alanine; E, Glutamic acid; J, meso-diaminopimelic acid; M, methionine; G, glycine; "=" indicates a cross link between two disaccharide peptide. **b,** PCA plot shows clear separation of the groups from each other suggesting altered peptidoglycan fragment between sample sets.

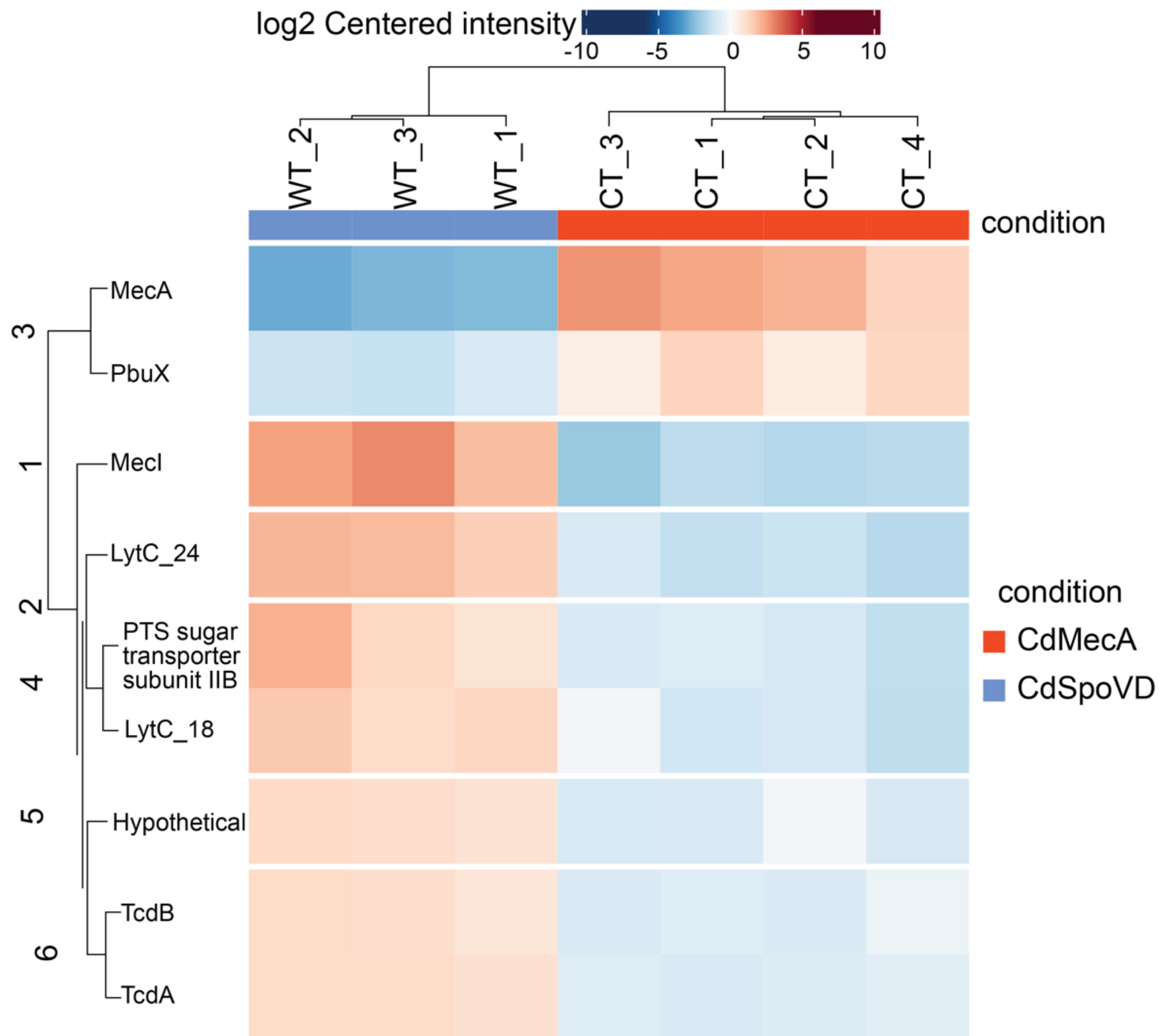

Supplementary Figure 8. Hierarchical clustering analysis of significantly dysregulated *Cd*MecA and *Cd*SpoVD proteins. *Cd*MecA spores (red) and *Cd*SpoVD spores (blue). N=3-to-4, Log_2 fold change ≥ 0.75 and an FDR adjust $P \leq 0.05$.

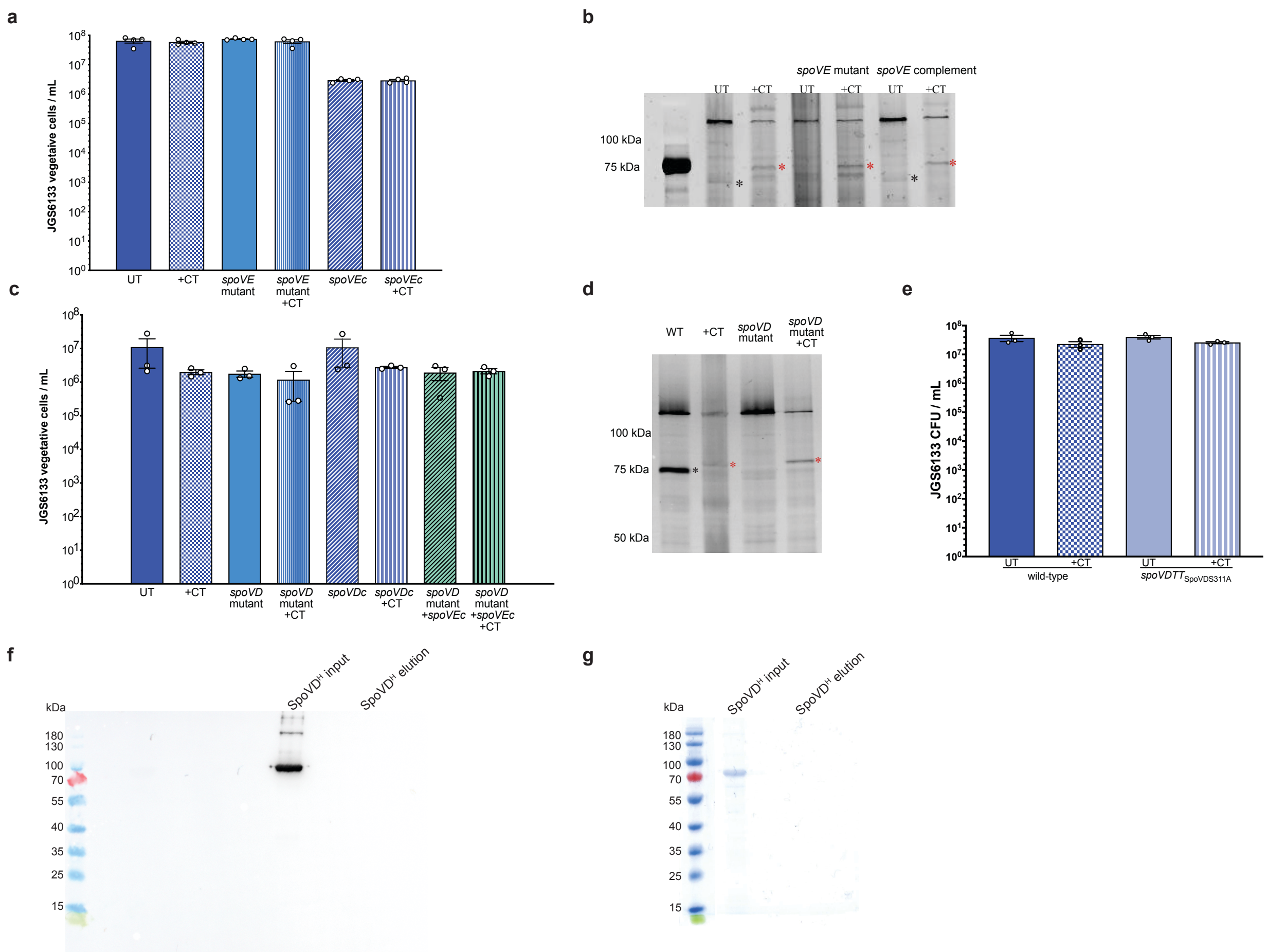

Supplementary Figure 9. Cefotetan has no impact on JGS6133 *CdspoVE* mutant, *CdspoVD* mutant and *CdspoVDTT*$_{\mathbf{SpoVDS311A}}$ strain total viable cell growth, confirmation of *Cd*MecA induction in cefotetan-treated *CdspoVE* and *CdspoVD* mutant PBP profiles and Western Blot and Coomassie stain of His-tagged SpoVD input and elution controls. a, Total viable counts of sporulating JGS6133 untreated and cefotetan-treated wild-type, *CdspoVE* mutant and *CdspoVE* complemented cultures were determined. **b,** Membrane proteins from sporulating JGS6133 wild-type and *CdspoVE* mutant cultures left untreated or treated with cefotetan were isolated, incubated with Boc–FL and separated by SDS–PAGE. Labelled PBPs were visualized using a FluorImager. Black asterisks indicate *Cd*SpoVD which is present in untreated cultures but not in cefotetan-

treated and *CdspoVE* mutant and *in trans* complemented cultures, red asterisks indicate *Cd*MecA which is present in cefotetan-treated cultures but not in untreated and *CdspoVE* mutant and *in trans* complemented cultures. **c,** Total viable counts of sporulating untreated and cefotetan-treated JGS6133 wild-type, *CdspoVD* mutant, *CdspoVD* complemented, and *CdspoVD*+*CdspoVEc* cultures were determined. **d,** Membrane proteins from sporulating wild-type JGS6133 and JGS6133 *CdspoVD* mutant cultures were left untreated or treated with cefotetan, were isolated, incubated with Boc–FL and separated by SDS–PAGE. Labelled PBPs were visualized using a FluorImager. Black asterisk indicates *Cd*SpoVD which can be visualized in the untreated culture but not visualized in the untreated *CdspoVD* mutant culture (absence of *Cd*SpoVD and therefore can't bind to Boc-FL) or the cefotetan-treated wild-type (*Cd*SpoVD bound to cefotetan and therefore can't bind to Boc-FL) and *CdspoVD* mutant culture (absence of *Cd*SpoVD and therefore can't bind to Boc-FL), red asterisks indicate *Cd*MecA which is present in cefotetan-treated treated cultures. **e,** Total viable counts of sporulating JGS6133 cultures of untreated and cefotetan-treated wild-type and untreated and cefotetan-treated *CdspoVDTT*$_{\mathrm{SpoVDS311A}}$ were determined. UT=untreated; +CT=cefotetan-treated; *spoVE*c=*spoVE* complemented; *spoVD*c=*spoVD* complemented. For sporulation assays, results are the average of three-four independent biological replicates. Error bars represent s.e.m.; P values were determined using a Kruskal-Wallis test with Dunn's pairwise comparisons. No statistical difference in total viable counts were seen between the samples for all viable cells count sporulation assays. **f,** Western Blot and **g,** Coomassie stain of recombinant SpoVD input and elution fractions from Strep-Tactin. Source data are provided as a Source Data file.

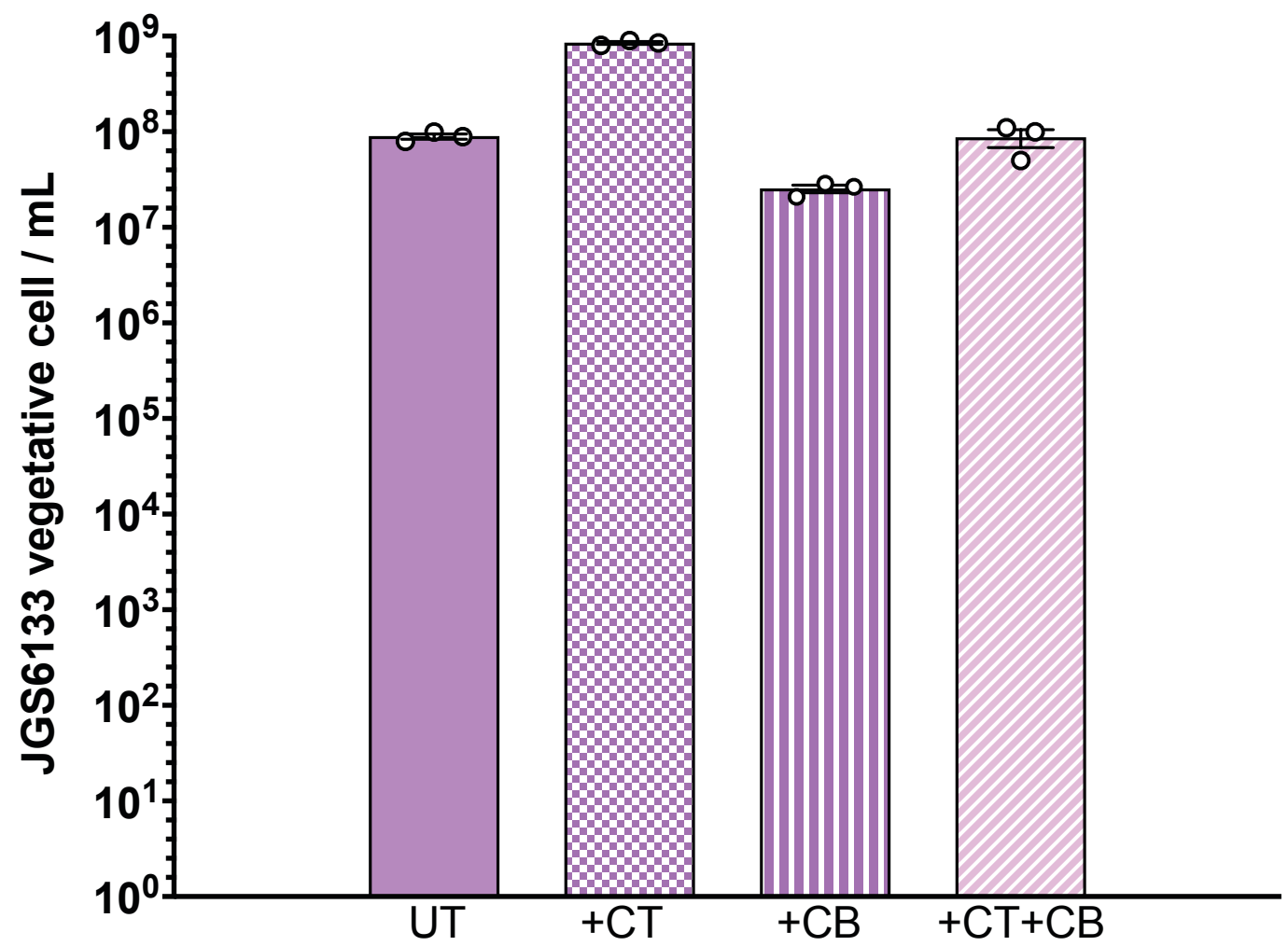

Supplementary Figure 10. Ceftobiprole has no impact on JGS6133 total viable cell growth. Using sporulation assays JGS6133, vegetative cell numbers (cfu/mL) were determined for cultures from wild-type strains; untreated (UT), cefotetan-treated (+CT), ceftobiprole-treated (+CB) and co-treated with cefotetan and ceftobiprole (+CT+CB). Results are the average of three independent biological replicates. Error bars represent s.e.m.; P values were determined using a Kruskal-Wallis test with Dunn's pairwise comparisons. No statistical difference was seen between all untreated and treated total viable counts. Source data are provided as a Source Data file.

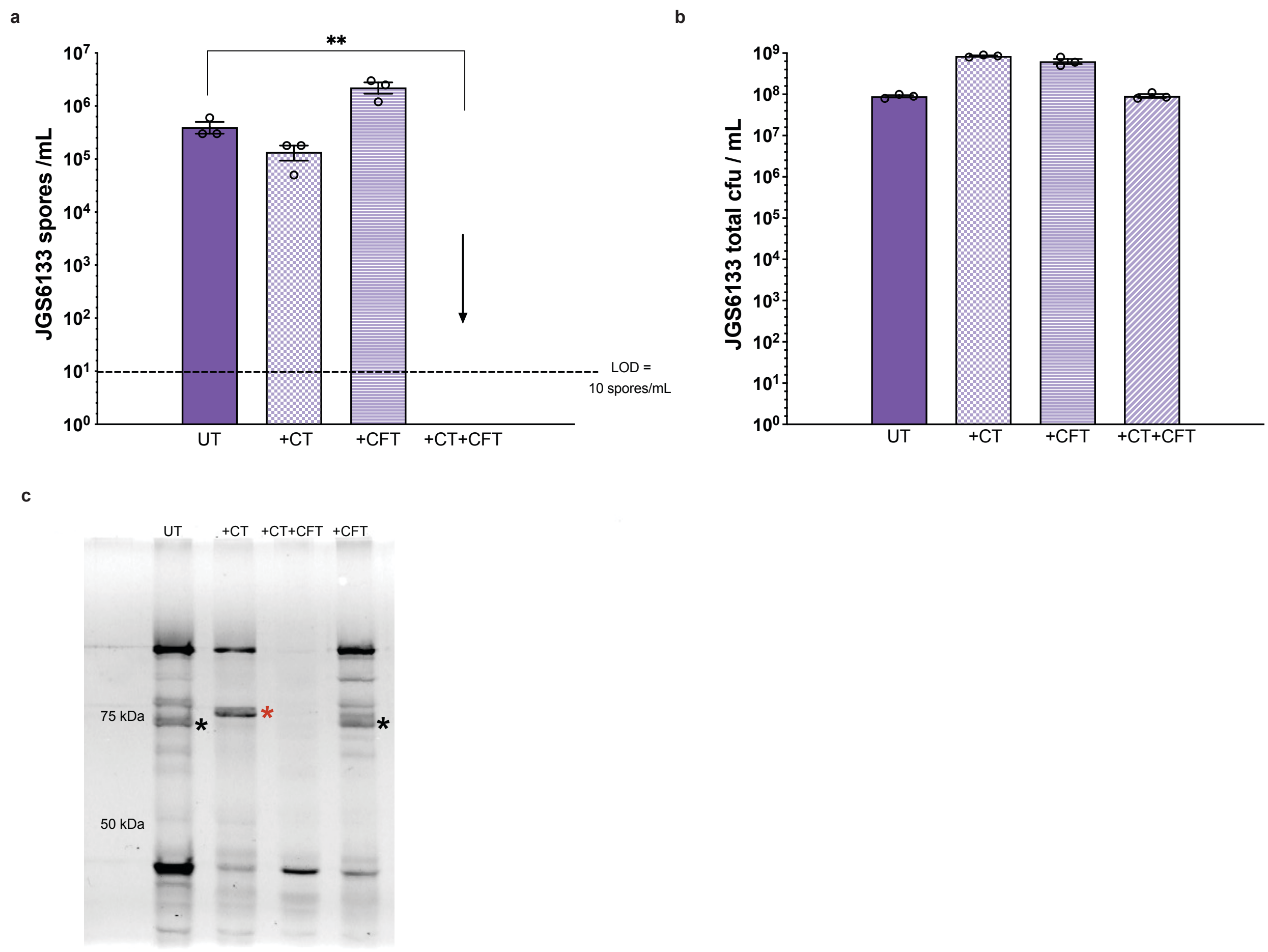

Supplementary Figure 11. Co-treatment with cefotetan and ceftaroline abolishes sporulation in *C. difficile* JGS6133 but does not impact viable cell counts. a, Spore numbers were determined from three independent cultures of strain JGS6133; untreated and cefotetan-treated wild-type, ceftaroline-treated wild-type and wild-type co-treated with cefotetan and ceftaroline. Error bars represent s.e.m, *P* values were determined using a Kruskal-Wallis test with Dunn's pairwise comparisons. Co-administration of cefotetan and ceftobiprole significantly reduced spore numbers compared to untreated cultures (***P*=0.0064). No significant differences in spore numbers were seen between untreated cultures and cultures treated with cefotetan only (*P*=0.464) and ceftaroline only (*P*=0.297). Limit of detection (LOD) shown as a horizontal dotted line. **b,** JGS6133, vegetative cell numbers (cfu/mL) were determined for cultures from wild-type strains;

untreated (UT), cefotetan-treated (+CT), ceftaroline-treated (+CFT) and co-treated with cefotetan and ceftaroline (+CT+CFT). Results are the average of three independent biological replicates. Error bars represent s.e.m.; P values were determined using a Kruskal-Wallis test with Dunn's pairwise comparisons. No statistical difference was seen between all untreated and treated total viable counts. **c,** Membrane proteins from sporulating wild-type JGS6133 untreated cultures (UT), cefotetan-treated cultures (+CT), cultures co-treated with cefotetan and ceftaroline (+CT+CFT) or treated with ceftaroline only (+CFT) were isolated, incubated with Boc–FL and separated by SDS–PAGE. Labelled PBPs were visualized using a FluorImager. The black asterisks indicate *Cd*SpoVD (73 kDa), and the red asterisks indicate *Cd*MecA (76 kDa). Source data are provided as a Source Data file.

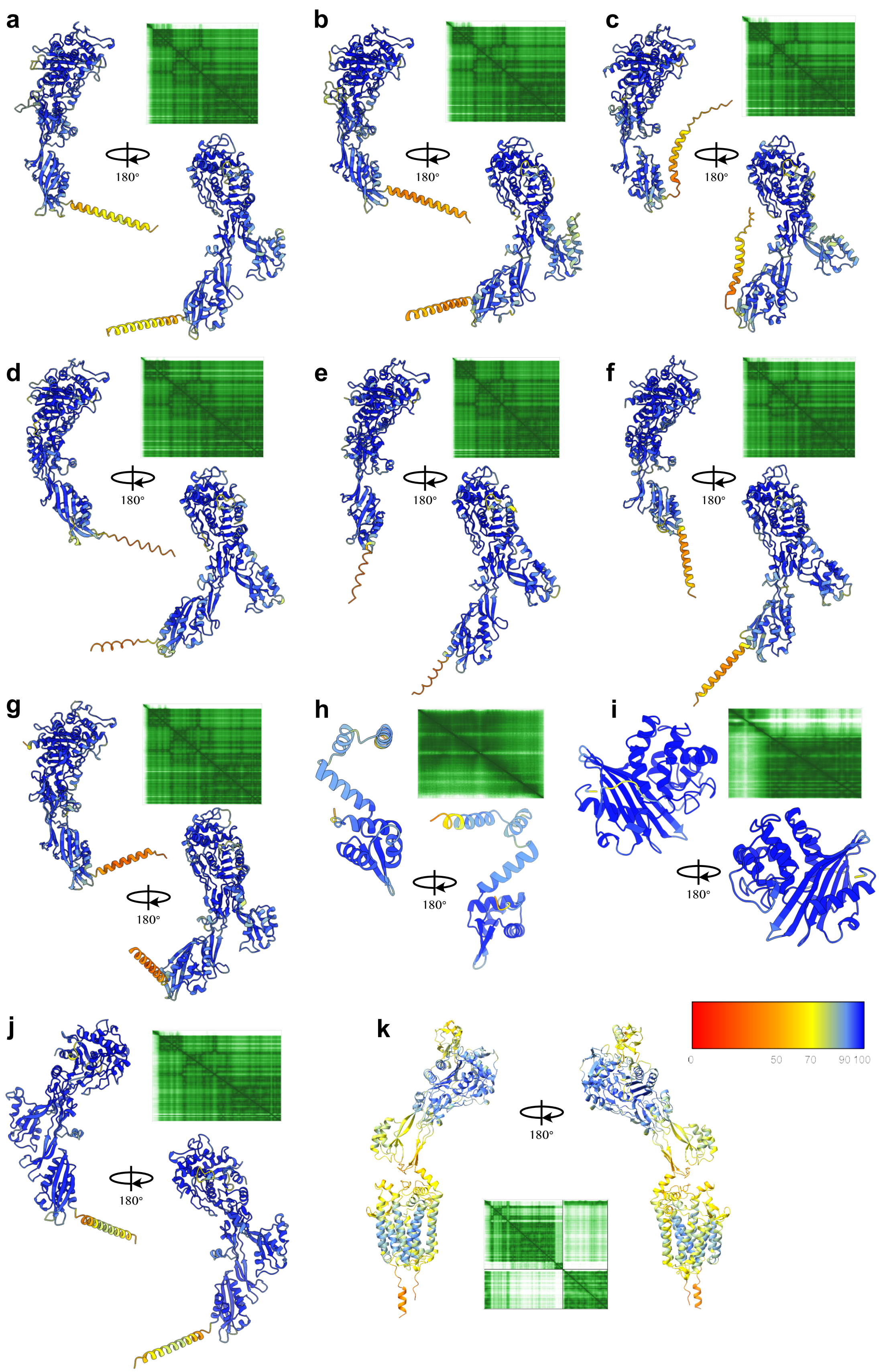
a
b
c
d
e
f
g
h
i
j
k
180°
0
50
70
90 100

Supplementary Figure 12. AlphaFold models coloured by pLDDT scores from two angles with corresponding predicted aligned error. AlphaFold3 models of MecA proteins from *C. difficile* (a), *B. wrexlae* (b), *N. circulans* (c), *B. amyloliquefaciens* (d), *T. bilis* (e), *P. sordellii* (f), and *M. australensis* (g). MecI (h), $MecR_{246\text{-}599}$ (i) MecA (j) and SpoVD:SpoVE complex (k) from *C. difficile.*

Supplementary Table 1. Minimum and sub-minimum inhibitory concentration of cephamycins for *C. difficile* strains MA0129, JGS6133 and A3491.

**Strain**	**Origin**	**Sub minimum inhibitory concentration & Minimum inhibitory concentration (μg/mL)**			
C. difficile strains		Cefoxitin	Cefotetan	Cefmetazole	Ceftobiprole/ Ceftaroline
MA0129	Human clinical isolate, Malaysia; RT017, clade 4. $A^{-}B^{+}CDT^{-}$, Tom Riley University of Western Australia	20<br>30	20<br>30	20<br>30	N/A
JGS6133	Porcine isolate, RT078, clade 5, $A^{+}B^{+}CDT^{+}$ USA, [10]	20<br>30	5<br>10	10<br>20	5, 10<br>10, 20
A3491	Environmental soil isolate, Australia, RT unknown, clade C-III, $A^{-}B^{-}CDT^{-}$, Tom Riley University of Western Australia	20<br>30	20<br>30	20<br>30	N/A
C. perfringens					
DLL6642	Human gut isolate, Monash Health	N/A	0.078<br>0.156	N/A	N/A
E. coli strains					
HB101	Laboratory strain containing pVS520 used as donor in conjugation with *C. difficile*: *thi-1 hsdS20*(rB−, mB−) *supE44 recAB ara-14 leuB5 proA2 lacY1 galK rpsL20 (*Sm^{R}*) xyl-5 mtl-1* [11]				
BL21(DE3)	Protein expression strain *F – ompT hsdSB* (rB- mB-) *gal dcm* (DE3) NEB				

Supplementary Table 2. MecA, MecR and MecI AlphaFold2 confidence scores.

C. difficile JGS6133	Predicted Local Distance Difference Test (pLDDT)	Predicted Template Modelling (pTM)
$MecA_{38-692}$	93.4	0.855
$MecR_{246-599}$	92.4	0.771
MecI	90.4	0.747

Supplementary Table 3. **Minimum inhibitory concentration of cephamycins for JGS6133 (WT), cefotetan-treated JGS6133 (+CT) and JGS6133 *mecA*TT to ascertain *CdmecA* vegetative cell resistance to cephamycins.**

C. difficile	**Minimum inhibitory concentration (µg/mL)**		
	Cefoxitin	Cefotetan	Cefmetazole
JGS6133	100	12.5	25
JGS6133 +CT	100	12.5	12.5
JGS6133 *mecA*TT	100	25	25

Supplementary Table 4. Distribution of *CdmecA* among different Clades.

Clade	N genomes	N *CdmecA* positive	N STs	N STs *CdmecA* positive	N *CdmecI* positive	N *CdmecR* positive
1	6713	317 (4.7%)	154	12 (7.8%)	6516 (97.1%)	6496 (96.8%)
2	1951	3 (0.2%)	41	2 (4.9%)	1851 (99.6%)	1841 (94.4%)
3	237	0 (0.0%)	7	0 (0.0%)	236 (94.9%)	234 (98.7%)
4	557	227 (40.8%)	32	21 (65.6%)	549 (98.6%)	546 (98.0%)
5	847	470 (55.5%)	18	11 (61.1%)	836 (98.7%)	835 (98.6%)
C-I	19	0 (0.0%)	13	0 (0.0%)	14 (73.7%)	1 (5.3%)
C-II	4	0 (0.0%)	3	0 (0.0%)	3 (75%)	1 (25.0%)
C-III	2	0 (0.0%)	2	0 (0.0%)	0 (0.0%)	0 (0.0%)

Supplementary Table 5. ***C. difficile*** **STs with 100% presence of** ***CdmecA*****.**

ST	Clade	No. of genomes
81	4	19
316	5	3
559	4	3
172	1	2
241	4	2
254	4	2
401	4	2
658	4	2
153	1	1
158	4	1
260	4	1
273	4	1
317	5	1
327	4	1
386	5	1
387	5	1
392	5	1
505	1	1
634	4	1
635	4	1
640	1	1
650	4	1

Supplementary Table 6. *C. difficile* plasmids used in this study.

Plasmid	Origin/Source of plasmid
pVS520	Conjugative plasmid: Tra+, Mob+, RP4 plasmid derivative, Tet^R [12]
pMTL9361	*E. coli*-*C. difficile* mobilizable shuttle vector, carries pCD6 replication region Tm^R[13]
pDLL46	pMTL9361 derivative with *Hin*dIII site and single *Bsr*GI site; carrying *lacZα*; Tm^R [14]

Supplementary Table 7. Targetron gene fragments and primers used in this study.

<table>
<tr><td colspan="2">CdMecA</td></tr>
<tr><td colspan="2">AAAAAAGCTTATAATTATCCTTACTACACGAGTCGGTGCGCCCAGATAGGGTGTTAAGTC
AAGTAGTTTAAGGTACTACTCTGTAAGATAACACAGAAAACAGCCAACCTAACCGAAAAG
CGAAAGCTGATACGGGAACAGAGCACGGTTGGAAAGCGATGAGTTACCTAAAGACAATC
GGGTACGACTGAGTCGCAATGTTAATCAGATATAAGGTATAAGTTGTGTTTACTGAACGC
AAGTTTCTAATTTCGGTTTGTAGTCGATAGAGGAAAGTGTCTGAAACCTCTAGTACAAAGA
AAGGTAAGTTAAGCGACTCGACTTATCTGTTATCACCACATTTGTACAATCTG</td></tr>
<tr><td colspan="2">CdSpoVE</td></tr>
<tr><td colspan="2">AAAAAAGCTTATAATTATCCTTAAGATTCGTTGGAGTGCGCCCAGATAGGGTGTTAAGTC
AAGTAGTTTAAGGTACTACTCTGTAAGATAACACAGAAAACAGCCAACCTAACCGAAAAG
CGAAAGCTGATACGGGAACAGAGCACGGTTGGAAAGCGATGAGTTACCTAAAGACAATC
GGGTACGACTGAGTCGCAATGTTAATCAGATATAAGGTATAAGTTGTGTTTACTGAACGC
AAGTTTCTAATTTCGATTAATCTTCGATAGAGGAAAGTGTCTGAAACCTCTAGTACAAAGA
AAGGTAAGTTACATCCAACGACTTATCTGTTATCACCACATTTGTACAATCAATCT</td></tr>
<tr><td colspan="2">CdspoVD</td></tr>
<tr><td colspan="2">See [15]</td></tr>
<tr><td colspan="2">CdmecI</td></tr>
<tr><td colspan="2">AAAAAAGCTTATAATTATCCTTAGTTTTCGGACTCGTGCGCCCAGATAGGGTGTTAAGTCA
AGTAGTTTAAGGTACTACTCTGTAAGATAACACAGAAAACAGCCAACCTAACCGAAAAGC
GAAAGCTGATACGGGAACAGAGCACGGTTGGAAAGCGATGAGTTACCTAAAGACAATCG
GGTACGACTGAGTCGCAATGTTAATCAGATATAAGGTATAAGTTGTGTTTACTGAACGCA
AGTTTCTAATTTCGATTAAAACTCGATAGAGGAAAGTGTCTGAAACCTCTAGTACAAAGA
AAGGTAAGTTATGGAGTCCGACTTATCTGTTATCACCACATTTGTACAATCT</td></tr>
<tr><td colspan="2">CdmecR</td></tr>
<tr><td colspan="2">AAAAAAGCTTATAATTATCCTTAATATCCGATGTAGTGCGCCCAGATAGGGTGTTAAGTCA
AGTAGTTTAAGGTACTACTCTGTAAGATAACACAGAAAACAGCCAACCTAACCGAAAAGC
GAAAGCTGATACGGGAACAGAGCACGGTTGGAAAGCGATGAGTTACCTAAAGACAATCG
GGTACGACTGAGTCGCAATGTTAATCAGATATAAGGTATAAGTTGTGTTTACTGAACGCA
AGTTTCTAATTTCGGTTGATATCCGATAGAGGAAAGTGTCTGAAACCTCTAGTACAAAGA
AAGGTAAGTTACTTACATCGACTTATCTGTTATCACCACATTTGTACAATCT</td></tr>
<tr><td>CdmecAF1</td><td>ATGGCAGTCAAGCGTCATATTTCCAG</td></tr>
<tr><td>CdmecAR1</td><td>TAACCTCTGTATTAGATATTTTAACTCCAGGAA</td></tr>
<tr><td>CdmecRF1</td><td>GAAGAAGAATACGTAGCTACAGAAAGTAGTT</td></tr>
<tr><td>CdmecRR1</td><td>CGTTGATATCTGCCATAATAGATAGTTTAGG</td></tr>
<tr><td>CdmecIF1</td><td>GGAAATATGCTCCTATCAGCACTACTG</td></tr>
<tr><td>CdmecIR1</td><td>CCCCTTAGTCGTCCTTTTATTTAAGATTTC</td></tr>
<tr><td>CdspoVEF1</td><td>CCGCCGTCTAGATAAAGGAGTGGAACACAGACAAGAGTTTG</td></tr>
<tr><td>CdspoVER1</td><td>CCACTTCTCGTAGTTCCAGGGA</td></tr>
<tr><td>CdspoVDF1</td><td>AAAAAAGCTTATAATTATCCTTA [15]</td></tr>
<tr><td>CdspoVDR1</td><td>CAGATTGTACAAATGTGGTGATAACAGATAAGTC [15]</td></tr>
<tr><td>CdmecI_compF_SphI</td><td>GGGACATGCATGCACATTAATAGCTTCTTTTGGGCACCAGTGAA</td></tr>
<tr><td>CdmecA_compR_SacI</td><td>GGGACATGAGCTCTATACAAATAGTGGCAACTCTCCCT</td></tr>
<tr><td>CdspoVE_compF_ AatII</td><td>CCGCCGGACGTCTAAAGGAGTGGAACACAGACAAGAGTTTG</td></tr>
<tr><td>CdspoVE_compR_ SacI</td><td>CGCCGGAGCTCTTAATTAATTTTGACATGCTTTGAGATGTTT</td></tr>
<tr><td>CdmecRF1</td><td>GGAAATATGCTCCTATCAGCAC TACTG</td></tr>
<tr><td>CdmecRR1</td><td>CCCCTTAGTCGTCCTTTTATTTA AGATTTC</td></tr>
<tr><td>CdmecI_comp_F_BamHI</td><td>GTCAGGATCCCTGAGTAAAAATCGTATAAAGAATGGGGC</td></tr>
<tr><td>CdmecI_comp_R _SacI</td><td>GACAGAGCTCGTAGAAGGAGGTATAGTATGAAAAAGTTACCAC</td></tr>
</table>

CdmecR_comp_F_BamHI	AGCGTTAACAGATCTGAGCTCCTTAAATAAAAGGACGACTAAG
CdmecR_comp_R_SacI	GACAAAAAAATCTATAATCCCTAAGGATCCTATAAGTTTTAATAAAACTT
MecAF dPCR	TGGATATACTCTAACCTCTG
MecAR dPCR	GATAAGGAACTACAAGAGTC
MecIF dPCR	TACGTAGCTACAGAAAGTAG
MecIR dPCR	CTCTCAGTTCCTCAATATC
MecRF dPCR	GTAAACTGGTACTTTCAGAC
MecRR dPCR	GATACCACCTGAAACATC

Supplementary Data 1. (separate file)

Supplementary Data 2. (separate file)

Supplementary Data 3. (separate file)

Supplementary Data 4. (separate file)

Supplementary Data 5. (separate file)

Supplementary Data 6. (separate file)

Supplementary Data 7. (separate file)

Supplementary Data 8. (separate file)

Supplementary References

1 Hiramatsu, K., Asada, K., Suzuki, E., Okonogi, K. & Yokota, T. Molecular cloning and nucleotide sequence determination of the regulator region of mecA gene in methicillin-resistant *Staphylococcus aureus* (MRSA). *FEBS Letters* **298**, 133-136 (1992). https://doi.org/https://doi.org/10.1016/0014-5793(92)80039-J

2 Niemeyer, D. M., Pucci, M. J., Thanassi, J. A., Sharma, V. K. & Archer, G. L. Role of mecA transcriptional regulation in the phenotypic expression of methicillin resistance in *Staphylococcus aureus*. *Journal of Bacteriology* **178**, 5464-5471 (1996). https://doi.org/doi:10.1128/jb.178.18.5464-5471.1996

3 Ubukata, K., Nonoguchi, R., Matsuhashi, M. & Konno, M. Expression and inducibility in *Staphylococcus aureus* of the mecA gene, which encodes a methicillin-resistant *S. aureus*-specific penicillin-binding protein. *Journal of Bacteriology* **171**, 2882-2885 (1989). https://doi.org/doi:10.1128/jb.171.5.2882-2885.1989

4 Imwattana, K., Rodríguez, C., Riley, T. V. & Knight, D. R. A species-wide genetic atlas of antimicrobial resistance in *C. difficile*. *Microb Genom* **7**, e000696 (2021). https://doi.org/https://doi.org/10.1099/mgen.0.000696

5 Gilchrist, C. L. M. & Chooi, Y.-H. clinker & clustermap.js: automatic generation of gene cluster comparison figures. *Bioinformatics* **37**, 2473-2475 (2021). https://doi.org/10.1093/bioinformatics/btab007

6 Mirdita, M. *et al.* ColabFold: making protein folding accessible to all. *Nature Methods* **19**, 679-682 (2022). https://doi.org/10.1038/s41592-022-01488-1

7 Lim, D. & Strynadka, N. C. J. Structural basis for the β lactam resistance of PBP2a from methicillin-resistant Staphylococcus aureus. *Nature Structural Biology* **9**, 870-876 (2002). https://doi.org/10.1038/nsb858

8 Alexander, J. A. N. *et al.* Structural analysis of avibactam-mediated activation of the bla and mec divergons in methicillin-resistant *Staphylococcus aureus*. *Journal of Biological Chemistry* **295**, 10870-10884 (2020). https://doi.org/10.1074/jbc.RA120.013029

9 Safo, M. K. *et al.* Crystal Structures of the BlaI Repressor from *Staphylococcus aureus* and Its Complex with DNA: Insights into Transcriptional Regulation of the *bla* and *mec* Operons. *Journal of Bacteriology* **187**, 1833-1844 (2005). https://doi.org/doi:10.1128/jb.187.5.1833-1844.2005

10 Mackin, K. E., Carter, G. P., Howarth, P., Rood, J. I. & Lyras, D. Spo0A differentially regulates toxin production in evolutionarily diverse strains of *Clostridium difficile*. *PLoS One* **8**, e79666 (2013). https://doi.org/10.1371/journal.pone.0079666

11 Boyer, H. W. & Roulland-Dussoix, D. A complementation analysis of the restriction and modification of DNA in *Escherichia coli*. *J Mol Biol* **41**, 459-472 (1969). https://doi.org/10.1016/0022-2836(69)90288-5

12 Palombo, E. A., Yusoff, K., Stanisich, V. A., Krishnapillai, V. & Willetts, N. S. Cloning and genetic analysis of tra cistrons of the Tra 2/Tra 3 region of plasmid RP1. *Plasmid* **22**, 59-69 (1989). https://doi.org/10.1016/0147-619x(89)90036-x

13 Carter, G. P. *et al.* Binary Toxin Production in *Clostridium difficile* is Regulated by CdtR, a LytTR Family Response Regulator. *Journal of Bacteriology* **189**, 7290-7301 (2007). https://doi.org/doi:10.1128/jb.00731-07

14 Lyon, S. A., Hutton, M. L., Rood, J. I., Cheung, J. K. & Lyras, D. CdtR Regulates TcdA and TcdB Production in *Clostridium difficile*. *PLoS Pathog* **12**, e1005758 (2016). https://doi.org/10.1371/journal.ppat.1005758

15 Srikhanta, Y. N. *et al.* Cephamycins inhibit pathogen sporulation and effectively treat recurrent *Clostridioides difficile* infection. *Nature Microbiology* **4**, 2237-2245 (2019). https://doi.org/10.1038/s41564-019-0519-1
