# Supplementary material for: Uncovering an alternate pathway of antibiotic resistance in spore-forming bacteria
Source: Nat Commun. 2026 Jul 15;17:8674. doi: 10.1038/s41467-026-75594-5 (PMC13490422; doi:10.1038/s41467-026-75594-5)
Supplement: Supplementary file 4 [file 41467_2026_75594_MOESM2_ESM.pdf]

Description of Additional Supplementary Files

Supplementary Data 1. *C. difficile* MecAIR screening.

Supplementary Data 2. Database used for the first PGFinder search.

Supplementary Data 3. Database used for the second PGFinder search.

Supplementary Data 4. *Cd*SpoVD and *Cd*MecA Spore PG structural analysis.

Supplementary Data 5. Proteomics *Cd*MecA spores vs *Cd*SpoVD spores.

Supplementary Data 6. Co-IP Mass spec *Cd*MecA-*Cd*SpoVD protein ID report.

Supplementary Data 7. Co-IP Mass spec *Cd*MecA-*Cd*SpoVD analysis.

Supplementary Data 8. AlphaFold2 models were generated in ColabFold using truncated *Cd*MecA and *Cd*MecR sequences to correspond with previously solved *S. aureus* PDB structures.
