# Supplementary material for: Uncovering an alternate pathway of antibiotic resistance in spore-forming bacteria
Source: Nat Commun. 2026 Jul 15;17:8674. doi: 10.1038/s41467-026-75594-5 (PMC13490422; doi:10.1038/s41467-026-75594-5)
Supplement: Supplementary file 5 [file 41467_2026_75594_MOESM8_ESM.pdf]

Report

Protein IDs using nanoLC ESI MS/MS and Byonic (ProteinMetrics)

1) General information

**Project number:**	2024_078
**Client:**	Clara Bate
**Report date:**	**10/07/2024**
**Report prepared and released by:**	David Steer

2) Samples

**Number of samples:**	1
**Sample details:**	SpoVD MecA

3) Sample preparation

**Sample prep SOP:**	PQMS-MBPF-WIN-0138
**Prepared by:**	David Steer

4) Mass spectrometric acquisition

**Nano LC System:**	Dionex Ultimate 3000 RSLCnano
**Mass spectrometer:**	Eclipse (Thermo Scientific)
**Analytical column:**	Acclaim PepMap RSLC (75 µm x 50 cm, nanoViper, C18, 2 µm, 100Å; Thermo Scientific)
**Trap column:**	Acclaim PepMap 100 (100 µm x 2 cm, nanoViper, C18, 5 µm, 100Å; Thermo Scientific)
**Acquisition method:**	ECP_61_HCD_OT_mapping_FAIMS_2CV_V02
**Analysis date:**	9/07/2024

5) Data analysis

**Number of searches:**	1
**Search engine:**	Byonic (ProteinMetrics)
**Data base:**	Uniprot Ecoli incorporating supplied sequence
**Protein FDR cutoff**	1%
**Fixed modification**	Carbamidomethylation
**Variable modification**	Oxidation @ M

6) Attached files

- One Excel spreadsheet for each sample summarizing the search parameters as well as the identified peptides and proteins.

7) Results

Approx. 10 to 20 proteins have been identified in the samples which is expected from gel bands, with about half of these being confident matches to contaminants or other commonly abundant proteins, the remaining matches were less significant including matches of fewer than 3 peptides. Please consult the attached Excel spreadsheet on the proteins tab for the full list of proteins.

The provided sequences were matched in all samples. Below is the coverage map of the top protein match showing the regions of the sequence most significantly matched.

Matched peptides are displayed in green and in the case where a peptide spectrum has been matched more than once these are indicated as green lines below the sequence that was matched. Residues that were matched as modifications are displayed in red

Sample SpoVD MecA:

Sequence match:

>gi|2024078_spovd|Recombinant sequence spovd

Protein Coverage (double click to dock / undock)

Protein Coverage

Sample name	Protein name	Coverage summary	Coverage percent
Currently check...	>gi|2024078_spovd|Recombinant sequence spovd	162 of 660	24.55%

>gi|2024078_spovd|Recombinant sequence spovd

```
         10        20        30        40        50        60        70        80        90        100       110       120
MLRKVKRISKKRLVLVLILACALFFCLVIRTGYLQLMKGNWLSTKALEQQTRDIPIEPKRGTIYDRNMKELAVSVTKYTVWCKPVEVEDKKEAAEKVAEILDEDYKDIYALISKKNMALV
         130       140       150       160       170       180       190       200       210       220       230       240
KVKRWIDDDKASKIRDAKLSGIWVAEDNQRYYPYGNFAPYVLGHTSSDATGISGIEMQYDKKLKGKPGKLIVSTDASGREIPQGMEKYYEPVQGNGLVLSIDEVIQHYTEKAVQKAYELN
         250       260       270       280       290       300       310       320       330       340       350       360
NAKKVTAIAMNPKTGDILALASKPDYDPNDSRTPIYPYYQEELEKYNDKDKIKGYYQMWRNPAVSDTYEPGSTFKLITSSSALEEGVIKDGEKFTCTGSVTVGGRKIKCWRHYRPHGTQE
         370       380       390       400       410       420       430       440       450       460       470       480
FKQAVQNSCNPVFVELGSRLGVGKMYDYIESFGLMDKTGIDLPGEAKGILYNEKNVGPVELATISFGQSISVTPIQLITAISSIANGGDLMQPRVVKSYTDNKGNITETVKPKKVRSVIS
         490       500       510       520       530       540       550       560       570       580       590       600
KETSKKMLEIAESVVTEGGGKIAYIPGYRLGGKTGTAQKVIDGKYAPGKYICSFVGIAPCDDPQIVVLAIVDEPTGVSAFGSTTAGPIVKEIMNDSLKYLGVKPVYKEEEKAEYEKKQVK
         610       620       630       640       650
VPDVRNLKIGDAVKALEDVKLKPDLDADIELPEDTKVKDIFPKPGVKVNEDSSITLYFEN
```

Sequence match:

>gi|2024078_meca|Recombinant sequence meca

Protein Coverage (double click to dock / undock)

Protein Coverage

Sample name	Protein name	Coverage summary	Coverage percent
Currently check...	>gi|2024078_meca|Recombinant sequence meca	114 of 692	16.47%

>gi|2024078_meca|Recombinant sequence meca

```
         10        20        30        40        50        60        70        80        90        100       110       120
MRKNKKARKMIIFGFLMCLLIVIIVFLAKFILDGLNKNKPDEVFKQYMSFANKKQYEKMYDLLDEKSKSENKKEDFVLRNKKIYEGIDAHNISIKINDIKKNKNNDKLINYDSKMDTLAG
         130       140       150       160       170       180       190       200       210       220       230       240
EILFSNEVLLTRDRQKNYKIKWQSSVIFPELEEDNTVRVSKLKGKRGRILDRNGVMIAGQGLASMIGLVPGKMSDNIEDLKKLSTLLNVSVEQIEKKLNASWVKENSMVPIKTIEKIKEN
         250       260       270       280       290       300       310       320       330       340       350       360
PDGTVKEEDKELQESLLSIPGVKISNTEVRVYPFGEKTGHLTGYVQNVNAEILKEKEGKRYNANSIIGKIGLESLLEDRIRGIDGYEIIIADRYGDKKETLVTEPKVDGEDVKLTMDSKL
         370       380       390       400       410       420       430       440       450       460       470       480
QSKLYDQMKNDKGCAVVMNPKTGEVLSLVSSPSYNPNEFILGMSEDRWNELNKNENKPMYNRFKARLCPGSSFKPVTAGIGITTGKINPNENFGHSGLSWQKDSSWGSYKVTTLKDYGNT
         490       500       510       520       530       540       550       560       570       580       590       600
VNMENALIYSDNIYFAKAALKIGEDVLSKELLKLGFDESMPFEFGLSSSKFGTDNKFETEIQLADTGYGQGKLLVNPVHMASIYSAFVNDGDMIKPHLEFKKNLQAEFWKKGVFSKEAVK
         610       620       630       640       650       660       670       680       690
IIRDDLLQVVESPNGTGHSAMIKGIRIAGKTGTAEIKASKDDVTGTELGWFNAFTVDGNNDKQYLAIAMIEDVKSRGGSHYVMPIVKSVFED
```

8) Detailed explanation of the attached files

Excel spreadsheets

Each Excel spreadsheet has 3 tabs: Summary, Spectra and Proteins. The Proteins tab is the most important tab and shows the identified proteins. It is already sorted according to the probability that the protein identification is correct. For a more stringent analysis, MBPF recommends to ignore all proteins, which have only been identified by one peptide (column H: # of unique peptide). The following is an extensive description of the different tabs and columns:

- The Summary tab provides a quick overview of the results showing (i) the number of identified peptides and proteins, (ii) the search parameters, (iii) the Protein Score Plot as well as (iv) the Precursor Mass Errors. Users not familiar with mass spectrometry can ignore this tab.
- The Spectra tab summarizes all ms2 spectra, which were assigned to a peptide or in lay terms, the different peptides that have been identified. This list will contains redundant entries on the peptide level as multiple spectra can be assigned to the same peptide. Please note that all entries in blue represent peptides that match to a reverse decoy protein and are therefore wrong (a decoy database is used to calculate the various FDRs). Inexperienced end users can ignore this tab and/or most columns. Nevertheless, here is a short description of the columns (for more information, visit http://www.proteinmetrics.com or consult MBPF staff)
    - Query #:z: query number and charge state
    - Protein Rank: rank of the protein to which the peptide has been assigned
    - Peptide: peptide sequence. Please note that numbers in brackets correspond to post-translational modifications (PTMs). The most commons ones are carbamidomethylation [+57.021], oxidation [+15.995] as well as acetylation [+XX.XXX].
    - Glycans: indicates if the peptide carries a glycosylation (*if applicable)*
    - Modification: indicates if the peptide carries one of more PTMs. Blank: no PTM
    - Observed m/z: measure mass-to-charge ratio of the precursor (peptide)
    - Z: charge state
    - Observed (M+H): observed precursor mass of the singly charged peptide
    - Calc. mass (M+H): theoretical precursor mass of the singly charged peptide
    - Off-by-x error: [$M_{Observed}$ – $M_{Computed}$], where $M_{Observed}$ is the observed M+H (singly charged) precursor mass and $M_{Computed}$ is the computed M+H precursor mass, and [ ] means closest integer

- Mass error (ppm): 10^6 x ($M_{Observed}$ – $M_{Computed}$) / ($M_{Computed}$). The ppm mass error is computed after correcting for off-by-x errors.
- Starting position: Position within the protein of the N-terminal residue of the peptide.
- Cleavage: Digestion specificity, where Specific means fully specific, Nragged means nonspecific at the N-terminus, Cragged means nonspecific at the C-terminus, and Non means nonspecific at both termini.
- Score: Byonic score, the "raw" indicator of PSM correctness. Byonic scores reflect the absolute quality of the peptide-spectrum match, not the relative quality compared to other candidate peptides. Byonic scores range from 0 to about 1000, with 300 a good score, 400 a very good score, and PSMs with scores over 500 almost sure to be correct.
- Delta: The drop in Byonic score from the top-scoring peptide to the next *distinct* peptide. In this computation, the same peptide with different modifications is not considered distinct.
- Delta Mod: The drop in Byonic score from the top-scoring peptide to the next peptide different in any way, including placement of modifications. DeltaMod gives an indication of whether modifications are confidently localized; DeltaMod over 10.0 means that there is a high likelihood that all modification placements are correct.
- |Log Prob|: The absolute value of the log10 of the posterior error probability (PEP). The PEP takes into account the Byonic score, delta, precursor mass error, digestion specificity, and so forth (10 features in all). For PSMs with non-negligible error probabilities, say error probabilities > 0.0001, and hence |Log Prob| < 4.0, PEPs are in good agreement with "local FDR" measured by the decoy sequences. |Log Prob| incorporates more evidence than Byonic scores, so that sorting by |Log Prob| will almost always give a better ROC curve than sorting by score.
- # of unique peptides: Total number of PSMs for the protein that "owns" this PSM, discounting exact duplicates. The same peptide with the same modification differently placed counts as a distinct PSM.
- Protein name: Name of the protein the peptide has been assigned to
- Protein DB number: Protein number in the database
- Comment: The comment= value is from the TITLE= line in the .mgf. Depending upon what software wrote the .mgf, the TITLE= line sometimes contains the scan number from the raw file.
- Scan #: This is the scan number for the MS/MS spectrum.
- Scan Time: This is the time of the scan, usually reported in seconds (depending on the input data file format). This is usually also considered the retention time (R.T.) in LC-MS experiments.

- The Proteins tab summarizes all identified proteins. Please note that all entries in blue represent reverse decoy proteins and are therefore wrong (a decoy database is used to calculate the various FDRs). Nevertheless, here is a short

description of the columns (for more information, visit http://www.proteinmetrics.com or consult MBPF staff)

- Protein Rank: rank of the protein
- Description: Identified protein
- |Log Prob|: Absolute value of the log base 10 of the protein p-value
- Best |Log Prob| – Largest |Log Prob| of an individual PSM assigned to the protein
- Best Score – Largest Byonic score of a PSM assigned to the protein. For “one-peptide-per-protein” samples, for example topdown proteomics or N-glycopeptide samples, sorting by Best Score may give a better protein list (lower-ranking decoys) than protein |Log Prob|
- Total Intensity – Sum of all fragment peak intensities over all MS/MS spectra
- # of spectra – Total number of PSMs, including duplicate PSMs
- # of unique peptides – Total number of PSMs, discounting duplicates. (The same modification differently placed counts as a distinct PSM.)
- # of unique peptides – Total number of PSMs, discounting duplicates. (The same modification differently placed counts as a distinct PSM.)
- Coverage % – Percent of the protein sequence covered by PSMs
- # AA’s in protein: Protein size in amino acids
- Protein DB number: Protein number in the database
